# *In Silico* Optimization of a Bifunctional Lipase–Polyethylene Terephthalate (PET) Hydrolase for Enhanced PET and Lipid Hydrolysis

**DOI:** 10.1021/acs.jcim.6c00609

**Published:** 2026-05-29

**Authors:** Ana Robles-Martín, Paula Vidal, Rubén Muñoz-Tafalla, Jose L. Gonzalez-Alfonso, David Almendral, Francisco J. Plou, Manuel Ferrer, Laura Fernandez-Lopez, Víctor Guallar

**Affiliations:** † 132144 Barcelona Supercomputing Center (BSC), 08034 Barcelona, Spain; ‡ PhD program in Biotechnology, Faculty of Pharmacy and Food Sciences, 83076 University of Barcelona (UB), 08028 Barcelona, Spain; § Instituto de Catalisis y Petroquimica (ICP), CSIC, 28049 Madrid, Spain; ∥ Institució Catalana de Recerca i Estudis Avançats (ICREA), Barcelona 08010, Spain

## Abstract

Polyethylene terephthalate (PET) and lipid-rich waste pose urgent environmental challenges that need multifunctional biocatalysts. Lip_MRD9_, a lidless lipase with natural PETase activity, exemplifies such a scaffold. Here, we used an integrated approach combining automated *in silico* mutagenesis (AsiteDesign), Protein Energy Landscape Exploration (PELE), and molecular dynamics (MD) simulations, structure-guided rational engineering, and bioprospecting of homologous sequences to improve the dual lipase–PETase activity of Lip_MRD9_. Computational analyses pinpointed mutational hotspots extending the active-site binding region, guiding the creation of variants with better substrate binding and active-site geometry. Rationally engineered mutants such as RA18 and RA19, along with natural homologues, showed significant increases in PET breakdown (up to 2.8-fold) while maintaining or increasing lipase activity. Key design principles included aromatic substitutions that selectively expand subsite -I to boost PET affinity, surface charge adjustments, loop stabilization to promote substrate retention, and structural plasticity in evolutionary homologues that support dual reactivity. A comparison with natural homologues highlights the versatility of this scaffold for multifunctional uses. These findings demonstrate that designing lipase-derived PETases that effectively break down polyester while hydrolyzing lipids is achievable, providing a strong foundation for sustainable recycling of mixed PET–lipid waste streams.

## Graphical abstract

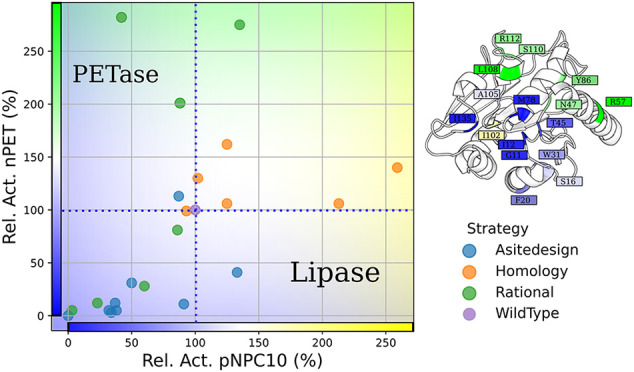

## Introduction

The accumulation of synthetic plastics and lipid-rich waste constitutes a pressing environmental challenge that requires innovative and sustainable solutions.^1^ Biocatalysis provides a compelling alternative to traditional chemical methods by using enzymes that operate under mild conditions, usually near-neutral pH and moderate temperatures (30–70 °C), thus avoiding harsh reagents and lowering energy usage.^2,3^ This strategy yields a lower environmental footprint, reduced carbon emissions, and enhanced economic viability, making enzymatic approaches appealing options for integrated recycling, upcycling processes, and, more broadly, biotransformations.^4^

Among biocatalysts, hydrolases such as cutinases, PETases, and lidless lipases have shown remarkable versatility by catalyzing the degradation of synthetic polymers, including polyethylene terephthalate (PET), polybutylene adipate terephthalate (PBAT), and nylon, as well as natural long triglycerides.^5,6^ This broad substrate scope highlights their potential as scaffolds for multifunctional biocatalytic applications. A recently characterized enzyme, Lip_MRD9_, exemplifies this versatility by belonging to subfamily I.4 of the largest bacterial lipase family (family I) and combining lipase and PETase activities within a single scaffold. Lip_MRD9_ hydrolyzes a wide range of triglycerides, including glyceryl tripropionate (TriC3:0), glyceryl tributyrate (TriC4:0), glyceryl tricaprylate (TriC8:0), glyceryl tricaprate (TriC10:0), glyceryl trilaurate (TriC12:0), and glyceryl trimyristate (TriC14:0), as well as natural oils such as coconut, olive, and palm oils. Also, it degrades PET in both film and nanoparticle formats. Additionally, the enzyme effectively hydrolyzes PET-derived oligomers and soluble intermediates, including TE and ETE, where T and E stand for terephthalic acid and ethylene glycol, respectively.^7^ This versatility has also been observed in other hydrolases, such as *Candida antarctica* lipase B (CALB), which naturally hydrolyzes triacylglycerols but can also catalyze various chemical processes, including the depolymerization of polyesters like polylactic acid (PLA) and the hydrolysis of small PET oligomers, although not PET itself, unlike Lip_MRD9_.

The open active site and inherent broad substrate specificity of Lip_MRD9_ provide significant opportunities for engineering variants with improved substrate binding and active-site structure.^8,9^ This can boost catalytic efficiency and allow for targeted enhancements of either lipase or PETase activity or both at the same time. This is especially relevant because lipases are among the most important industrial biocatalysts, with a global market value of $590.5 million in 2020.^10^ Additionally, the cost of enzymatic recycling using PETases is nearing approximately 4% of the market price of virgin PET per ton (US $25/kg of protein). Collectively, these factors emphasize the potential of enzymes with lipase and PETase activities as a practical foundation for building a bioeconomy based on oil waste and PET plastics.^11–13^

To this end, the compiled structural knowledge of PETases is extremely valuable as they are categorized into distinct families based on differences in active-site architecture and substrate-binding clefts. In fact, structurally, PETases are divided into different families depending on variations in the active-site structure and substrate-binding regions. They are generally classified as either type I or type II enzymes, with type II further subdivided into IIa and IIb subtypes.^14,15^ Type I PETases resemble traditional cutinases and have a relatively narrow substrate-binding cleft dominated by a conserved subsite I, where aromatic residues anchor the PET molecule for catalysis.^16,17^ Conversely, type II PETases include an extended loop that broadens the binding cleft, allowing them to accommodate large PET polymer chains. Subsite II displays more structural diversity and includes regions often labeled IIa, IIb, and possibly IIc, which interact with additional PET components or intermediate degradation products such as mono­(2-hydroxyethyl) terephthalate (MHET).^15^ This understanding is further reinforced by the accumulated knowledge of the reaction mechanisms involved in PET degradation facilitated by benchmark PETases.^18^

In this work, we applied computational structure-guided mutagenesis and sequence-informed screening to engineer Lip_MRD9_ variants with improved catalytic activity against both PET and TOL, focusing on PET. We used a multifaceted approach combining our Monte Carlo code, AsiteDesign, for guided substitutions under native-sequence constraints, rational redesign of catalytic and substrate-recognition residues, and targeted surface remodeling to expand the PET-binding interface.^19^ Additionally, we conducted comparative molecular dynamics (MD) simulations incorporating naturally evolved homologues with superior PET hydrolysis. These insights helped us select and prioritize mutations predicted to improve the substrate affinity and catalytic turnover. Lastly, we bioprospected highly related enzymes, obtaining improved variants and validating the promiscuity of the overall fold. These design and bioprospecting efforts enabled the development of new enzymes with tailored substrate specificities and better reactivity toward both PET and TOL, establishing Lip_MRD9_ as a robust and adaptable scaffold for bifunctional biocatalysts.

## Materials and Methods

### Initial Structural Model and Substrate Preparation

The Lip_MRD9_ and homologous sequences from the same sequence identity cluster were modeled using AlphaFold 2.0,^20^ ensuring high-confidence predictions. To enhance the accuracy of the structural models, residues with a pLDDT score of less than 90 were removed. We note that AlphaFold2 predictions are generally most reliable for well-packed core regions, whereas surface-exposed loops and peripheral segments, precisely the regions involved in engineering PET-binding paths and (in RA19) the designed disulfide, can show higher uncertainty. Accordingly, we treated the AlphaFold2 structures as starting scaffolds rather than as definitive static conformations.

Subsequent protein structure refinement was carried out using Schródinger’s Protein Preparation Wizard,^21^ where hydrogen bond networks and protonation states were optimized using PropKa at pH 7.0. The rational design mutants were generated using MAESTRO from Schródinger. PropKa was applied to compute the p*K*_*a*_ values of titrable residues and the values for histidine and acidic triad residues (Supporting Table 11).^22^

The substrate molecules were a PET tetramer (PT4 or eETETETETEe) with the terminal ethanol groups capped to more accurately represent the polymer environment and a triolein (TOL) molecule. The molecule was minimized using the OPLS2005 force field, and the substrate partial charges were obtained by Jaguar electrostatic potential (ESP) fitting from a DFT­(B3LYP-D3)/cc-pVTZ single-point calculation with implicit solvation (Jaguar SOLV) and were used for MD system setup. The complete per-atom charge sets are provided in the Supporting Tables 13 and 14.^23^

### Substrate Docking with Glide

We performed substrate-receptor docking using Schródinger’s Glide docking protocol with Extra Precision (XP) accuracy with PT4 and TOL and the different enzyme structures.^24^ The docking grid was centered on the oxygen atom of the catalytic serine (S77 in Lip_MRD9_), ensuring an accurate placement of the substrate for potential hydrolysis. We defined a small grid box of 10 Å to capture fine-scale interactions and a large grid box of 50 Å to explore broader binding conformations within the active site due to the big size of both substrates. For each enzyme–substrate system, up to 1000 docked poses were generated. The docked poses were then ranked using an explicit catalytic distance constraint: for every pose, we computed the distance between the nucleophilic Oγ atom of the catalytic serine and the carbonyl carbon of the candidate ester bond(s) in the substrate, and selected the pose minimizing this Ser Oγ–C­(carbonyl) distance. All catalytic distances were computed using MDAnalysis, enabling the identification of highly favorable enzyme–substrate interactions.^25^ The different systems were minimized via the Rosetta FastRelax protocol before simulations.^26^

### Binding Pocket Redesign Using AsiteDesign

The redesign of the active site followed the Monte Carlo AsiteDesign protocol,^19^ an automated computational enzyme engineering strategy that focuses on systematically redesigning residues within the active site to optimize substrate binding and catalytic efficiency. The initial input consisted of a PDB file with the substrate docked in the active site, complemented by ligand-specific parameters generated with Rosetta. The sampling strategy was coupled for both the active site and the substrate, while dynamic side-chain coupling was disabled to maintain a more constrained and physically relevant representation of the protein environment.

For active-site design, a minimization-based approach was applied, optimizing the interaction between the ligand and the surrounding residues. The optimization was performed over three iterative cycles, during which a subset of 35 residues was subjected to mutation and structural refinement. The residues selected for design were V9, H10, G11, I12, Y17, F19, F20, L26, A27, W31, R33, Q35, L36, K44, T45, A75, H76, M78, G79, G80, A81, N82, T101, I102, G103, G104, A105, and N106, L108, I135, V136, V137, S141, and I157. These residues were iteratively modified to enhance the ligand binding affinity while maintaining structural stability.

The substrate was treated as a rigid-body system with active packing enabled. Conformational sampling was performed using a Monte Carlo perturbation method with one perturbation loop and two random torsional perturbations applied. The adaptive sampling procedure consisted of 100 iterative cycles, generating a maximum of 100 final ligand poses. Each round of exploration comprised 25 steps, ensuring sufficient configurational space sampling while maintaining the computational feasibility. To enhance sampling efficiency, simulated annealing was employed, with an exponential temperature decay from an initial high-temperature regime (*kT* = 500) to a low-temperature state (*kT* = 1), thereby promoting convergence toward energetically favorable configurations.

### Computational Exploration of the Enzyme–Substrate Interaction

We used Protein Energy Landscape Exploration (PELE) software to analyze the interactions between the different enzymes and substrates.^27^ PELE is a heuristic Monte Carlo-based approach that models ligand-induced fit mechanisms by iteratively perturbing the enzyme–substrate complex and refining its conformation through energy minimization. The method begins with system perturbation, where different microstates of the ligand are explored through rotational and translational adjustments, while protein flexibility is accounted for using an Anisotropic Network Model (ANM). Next, steric clashes between the substrate and protein side chains are resolved, followed by a truncated Newton minimization using the OPLS2005 force field.^28^ The Metropolis criterion is then applied to determine whether the newly generated state is accepted or rejected. The system was parametrized using the OPLS2005 force field, with an implicit solvent model (VDGBNP) and ionic strength (0.15 M) and Debye length corrections to approximate the effect of water molecules surrounding the enzyme. Ligand flexibility was introduced using a steered translation-rotation perturbation scheme constrained within a 40 Å spherical box centered at the oxygen of the catalytic serine for each enzyme model. Each Monte Carlo (MC) move involved up to 500 steric trials with torsional flexibility regulated by an overlap factor of 0.65 to prevent excessive steric clashes while maintaining realistic conformational changes. Additionally, Adaptive ANM was integrated to explore global protein conformational dynamics, focusing on Cα atom displacements with six principal modes. To prevent excessive structural deviations, backbone constraints were applied to selected Cα atoms every 10 residues. Constraints were implemented with spring constants of 0.5 kcal·mol^–1^·Å^–2^, except for the first and last residues, which had a stronger spring constant of 5.0 kcal·mol^–1^·Å^–2^. To refine the interaction within the active site of the enzymes, we conducted two sequential rounds of simulations. We performed five PELE-independent simulation epochs of each of 100 Monte Carlo steps per iteration. The best Near-Attack Conformation (NAC) poses were selected to perform another 10 PELE-independent simulation epochs with 100 Monte Carlo steps with a previous equilibration phase using the last snapshot method (100 steps). The PELE binding energy was continuously monitored. A temperature scaling factor of 1500 K was applied to enhance the conformational sampling of enzyme–substrate complexes, preventing entrapment in local minima and promoting a broader exploration of the energy landscape. We used parallel execution on 96 processors, resulting in 95 trajectories.

NACs were defined as enzyme–substrate poses meeting the following criteria: (i) an ester carbon-to-serine oxygen distance of ≤ 5 Å, (ii) the catalytic triad remaining in a catalytic conformation lower than 4.5 Å, and (iii) a favorable binding energy threshold. In our PELE analysis, “binding energy” corresponds to the energy-like enzyme–substrate interaction score produced by the PELE all-atom force field energy and is used exclusively as a relative ranking metric across poses and variants. Because this score is not an absolute binding free energy, we did not impose a universal numerical cutoff in kcal·mol^–1^; instead, negative values indicate stabilizing interactions within the PELE all-atom force field energy (more negative = more favorable). These structural parameters were computed by using MDAnalysis.

Catalytic triads were filtered using the PELE interaction energy summary computed from a Boltzmann-weighted distribution to refine pose selection. The probability of each catalytic pose (*i*) was determined by

1 $$P_{i}=\frac{e^{-E_{i}/\left(\mathrm{kT}\right)}}{Q}$$

where *E*_*i*_ represents the total energy of the pose, *kT* is the energy partition constant, and *Q* is the partition function across *N* poses, defined as

2 $$Q=\sum_{i=1}^{N}e^{-E_{i}/\left(\mathrm{kT}\right)}$$

The expected catalytic binding free energy was then computed as

3 $$\left\langle E_{b}\right\rangle =\sum_{i=1}^{N}P_{i}E_{i}$$

This statistical approach ensured that only energetically favorable catalytic configurations were retained for further MD validation.

### Molecular Dynamics Simulations

To evaluate the stability and catalytic efficiency of the enzyme–substrate complexes, molecular dynamics (MD) simulations were performed using GROMACS 2023 with the AMBER99SB-ILDN force field for protein dynamics.^29^ Each system was solvated in a cubic TIP3P water box, ensuring a minimum 1.0 nm distance between the solute and the box edges, and then neutralized by adding the appropriate number of counterions (Na+/Cl−) to cancel the net charge. Additional Na+/Cl– ion pairs were then added to reach a final salt concentration of 0.15 M NaCl. Energy minimization was carried out by using the steepest descent algorithm until the maximum force was reduced below 1000 kJ/mol/nm. The system was equilibrated in two sequential steps: NVT equilibration at 298.15 K for 2000 ps, employing a V-rescale thermostat under constant volume conditions; and NPT equilibration in 10 consecutive 200 ps steps, with gradually decreasing positional restraints (from 1000 kcal/(mol Å^2^) to 5 kcal/(mol Å^2^)), using a Parrinello–Rahman barostat at 1.0 bar. Following equilibration, production MD simulations were conducted under NPT conditions for 200 ns per replica, with a 2 fs integration time step with all bonds involving hydrogen atoms constrained (LINCS). Temperature was maintained at 298.15 K using the Andersen thermostat, and the pressure was kept constant at 1.0 bar using the Parrinello–Rahman barostat. Electrostatic interactions were calculated using the particle-mesh Ewald (PME) method with a real-space cutoff of 1.0 nm. van der Waals interactions were truncated at the same 1.0 nm threshold. Periodic boundary conditions (PBC) were applied in all of the spatial dimensions. For each enzyme–substrate complex, three MD simulations of 200 ns were performed. Simulation trajectories were recorded for postprocessing and analysis using MDAnalysis.

We also computed longer simulations of 1 μs with Lip_MRD9_ wild-type and variants RA13 (L108Y) and RA18 (R57Y;L108Y), positioning the substrate in the docked position in the active site. In addition, three extra 1 μs replicas were performed for RA18 starting from a far-from-active-site configuration, in which the substrate was initially placed 25 Å from the catalytic serine (Supporting Table 12).

Trajectory analysis was performed using MDAnalysis,^25^ focusing on key catalytic distances (Ser–Substrate, Ser–His, His–Asp) throughout the simulations. The best Lip_MRD9_ variants for experimental validation were selected based on the percentage of total simulation time in which the catalytic triad remained in an optimal catalytic conformation lower than 4.5 Å.

### Screening of Lip_MRD9_ Homologous Sequences

The hydrolase Lip_MRD9_ (NCBI accession number: WP_034624255.1) was used as a query to screen for similar sequences in public databases. Searches were first performed in the Marine Metagenomics databases (MarRef and MarDB), from which Lip_MRD9_ was originally identified,^7^ and subsequently extended to the NCBI nonredundant protein sequence (nr) and UniProtKB (Swiss-Prot and TrEMBL) databases. Homologues were selected according to stringent cutoff criteria (percent identity >70%; alignment length >190 amino acids) against the 215-amino-acid lipase Lip_MRD9_. A total of 70 unique nonredundant sequences were retrieved, whose ID and source are shown in Supporting Data 1.

### Production of Lip_MRD9_ Mutants and Homologues

In brief, before gene synthesis, the sequence was analyzed for the presence of a signal peptide using the SignalP-5.0 tool (http://www.cbs.dtu.dk/services/SignalP/); a cleavage Sec/SPI site between positions 34 and 35 was predicted. The gene, excluding the signal peptide, was flanked by *Bam*HI and *Hin*dIII (stop codon) restriction sites and inserted into a pET-45b­(+) expression vector with an ampicillin selection marker (GenScript Biotech, Rijswijk, The Netherlands). This vector was subsequently introduced into *E. coli* BL21 (DE3). This plasmid, which was introduced into *E. coli* BL21 (DE3), supports the expression of N-terminal His6-fusion proteins, with the final amino acid sequence of the synthetic protein being MAHHHHHHVGTGSNDDDDKSPDPM-X (where X corresponds to the original sequence of the target enzyme without the signal peptide). After synthesis, the soluble N-terminal His_6_-tagged protein was produced and purified at >98% purity, as determined by SDS-PAGE using a Mini PROTEAN electrophoresis system (Bio-Rad, Madrid, Spain) at 4 °C after binding to a Ni-NTA His-Bind resin (Merck Life Science S.L.U., Madrid, Spain), and stored at −20 °C until use at a range of concentrations between 0.2 and 19.73 mg mL^–1^ in 40 mM 4-(2-hydroxyethyl)-1-piperazineethanesulfonic acid (HEPES) buffer (pH 7.0) 150 mM NaCl. All experimental details have been described elsewhere.^7^

### PET Degradation Tests and Analysis of Degradation Products

PETase activity was evaluated by monitoring the hydrolysis of submicro- and nanosized PET particles obtained from GoodFellow amorphous PET (Goodfellow Cambridge Ltd., Huntingdon, UK) (nPET_GFa_), produced as previously described.^30^ The physicochemical properties of these particles were as follows: average diameter, 80.47 ± 0.1 nm (range of 43.8 to 190 nm); glass transition temperature (Tg), 73.7 °C; melting temperature (*T*_m_), 245.9 °C; cold crystallization temperature (*T*_c_), 199.6 °C; crystallinity, 14.0%; and cold crystallization enthalpy (Δ*H*_c_), 41.5 J g^–1^. Hydrolysis of nPET_GFa_ was assessed under the following conditions: enzyme concentration, 4 μg; substrate concentration, 1.7 mg mL^–1^; reaction volume, 50 μL; buffer, 20 mM HEPES with 75 mM NaCl, pH 7.0; temperature, 40 °C; incubation time, 4 h. Reactions were carried out in 2 mL Safe-Lock Eppendorfpolypropylene tubes (Eppendorf SE, Hamburg, Germany; ref 0030120094) using a Thermomixer Comfort (Eppendorf AG, Hamburg, Germany) at 950 rpm. All assays, including controls, were performed in triplicate (*n* = 3) and terminated by adding 450 μL of dimethyl sulfoxide (Scharlau, Barcelona, Spain; ref SV165). Hydrolysis products were immediately analyzed by high-performance liquid chromatography (HPLC) using calibration curves with purified standards, as described by Robles-Martín et al.^30^

### Enzymatic Activity Assay Using 4-Nitrophenyl Decanoate (pNPC10)

The activity toward the model ester *p*NPC10 was assessed as described previously by monitoring the production of 4-nitrophenol at 348 nm (pH-independent isosbestic point, ε = 4147 M^–1^ cm^–1^) in 96-well plates (ref 655801, Greiner Bio-One GmbH, Kremsmúnster, Austria),^31^ with small modification. The kinetic measurements for the enzymatic hydrolysis of *p*NPC10 (Merck Life Science S.L.U., Madrid, Spain; ref: N0252) were conducted over a period of 10 min under the following reaction conditions: [Enzyme], 0.02 mg mL^–1^; [*p*NPC10], 1.2 mM (from a stock solution of 40 mM in acetonitrile); total reaction volume, 100 μL; buffer, 20 mM HEPES with 75 mM NaCl, pH 7.0; temperature, 40 °C. Reactions were carried out in 2 mL Safe-Lock Eppendorf polypropylene tubes (Eppendorf SE, Hamburg, Germany; ref 0030120094) using a 96-well U-bottom microplate (Greiner Bio-One, ref 650161). Data sets were collected with a Synergy HT Multi-Mode Microplate reader (with Gen5 2.00 software, Biotek Instruments, Winooski, VT, USA), with absorbance per min values obtained from the best linear fit using Excel 2019. Enzymatic activity was quantified by measuring the increase in absorbance per minute obtained from the best linear fit using Excel 2019. The activity was calculated by determining the absorbance per minute from the generated slopes. All reactions, including control reactions (see ref 31), and measurements were performed in triplicate (*n* = 3).

### Determination of Kinetic Parameters

#### PET Nanoparticles

Kinetic parameters (*K*_*m*_ and *k*_cat_) for PET nanoparticles were determined as previously described.^7,30^ Hydrolysis reactions were performed at 40 °C in 20 mM 4-(2-hydroxyethyl)-1-piperazineethanesulfonic acid (HEPES) buffer containing 75 mM NaCl (pH 7.0), using 0.1 mg mL^–1^ enzyme (native or variants) and substrate concentrations ranging from 0 to 2 g L^–1^ in a final reaction volume of 50 μL. Reactions were carried out in 2 mL Safe-Lock Eppendorf polypropylene tubes (Eppendorf SE, Hamburg, Germany; ref 0030120094) using a Thermomixer Comfort (Eppendorf AG, Hamburg, Germany) at 950 rpm for 4 h. All assays, including control reactions without enzyme, were performed in triplicate (*n* = 3). Reactions were terminated by the addition of 450 μL of dimethyl sulfoxide (DMSO; Scharlau, Barcelona, Spain; ref SV165). Hydrolysis products were immediately quantified by high-performance liquid chromatography (HPLC) using calibration curves prepared with purified standards as previously described.^7,30^ Initial rates were calculated from product formation, and kinetic parameters were obtained by nonlinear regression fitting to the Michaelis–Menten equation.

#### 4-Nitrophenyl Decanoate (pNPC10)

Kinetic parameters (*K*_*m*_ and *k*_cat_) for the model substrate 4-nitrophenyl decanoate (pNPC10) were determined as previously described.^31^ Reactions were carried out at 40 °C in 40 mM HEPES buffer containing 75 mM NaCl (pH 7.0), using 0.02 mg mL^–1^ enzyme (native or variants) and substrate concentrations ranging from 0 to 3 mM (from a 40 mM stock solution in acetonitrile) in a total reaction volume of 100 μL. Kinetic measurements were performed in 96-well flat-bottom microplates (Greiner Bio-One, ref 655801). The release of 4-nitrophenol was monitored at 348 nm (pH-independent isosbestic point; ε = 4147 M^–1^ cm^–1^) using a Synergy HT Multi-Mode Microplate Reader (BioTek Instruments, Winooski, VT, USA) controlled with Gen5 2.00 software. Initial rates were obtained from the best linear fit of absorbance versus time (typically within the first 30 min) using Excel 2019 (Microsoft). Enzymatic activity was calculated from the slopes (Abs/min), and kinetic parameters were determined by nonlinear regression fitting to the Michaelis–Menten model. All reactions, including control reactions without enzyme and substrate blanks, were performed in triplicate (*n* = 3).

## Results

We implemented three complementary strategies to enhance the dual lipase–PETase activity of Lip_MRD9_: (i) automated *in silico* engineering, (ii) structure-guided rational engineering, and (iii) homology-modeling-based bioprospecting of novel enzymes. In each case, a funnel-filtering screening approach was applied based on the presence of NACs identified through Monte Carlo and MD simulations. Triolein (TOL) was selected as the model substrate for lipase activity, as Lip_MRD9_ hydrolyzes it (0.86 ± 0.08 U mg^–1^), while also displaying activity toward triglycerides with acyl chains ranging from C2 (triacetin) to C18:1 (TOL), with specific activities from 0.22 ± 0.08 to 12.91 ± 0.39 U mg^–1^ protein at pH 8.0 and 30 °C. For PETase activity, eETETETETEe or PT4 (following the nomenclature of Schubert and colleagues;^32^), a soluble oligomeric PET fragment, was used as a model substrate, since PET oligomers of this size were detected during the hydrolysis of PET in nanoparticle and film formats, both of which were efficiently degraded by Lip_MRD9_. Specifically, Lip_MRD9_ degraded nPET_GFa_ at 30 °C (from 984 ± 20 μM after 21.5 h to 3903 ± 85 μM after 3 weeks) and at 40 °C (2238 ± 40 μM after 21.5 h), and PET_GFa_ at 30 °C (112 ± 20 μM degradation products after 7 days) and at 40 °C (198 ± 40 μM after 7 days).

### Automated Computational Identification of Active-Site Variants

A diverse library of 200 Lip_MRD9_ variants (100 for each substrate, TOL and PT4, respectively) was generated using the AsiteDesign software, an automated design tool.^19^ This approach incorporated iterative cycles of active-site remodeling and conformational sampling, with the catalytic triad of the lipase consisting of S77, H156, and D133. A total of 35 surface residues forming continuous grooves beyond the active site were selected, reflecting plausible PET-binding paths observed in docking simulations of tetrameric substrates (Figure 1A). The sampling protocol combined minimization-based optimization and Monte Carlo perturbation across 100 cycles, producing up to 100 final ligand poses per mutant. Given the substantial size of the data set, a stringent prioritization scheme was applied to identify the most promising candidates.

**Figure 1:**
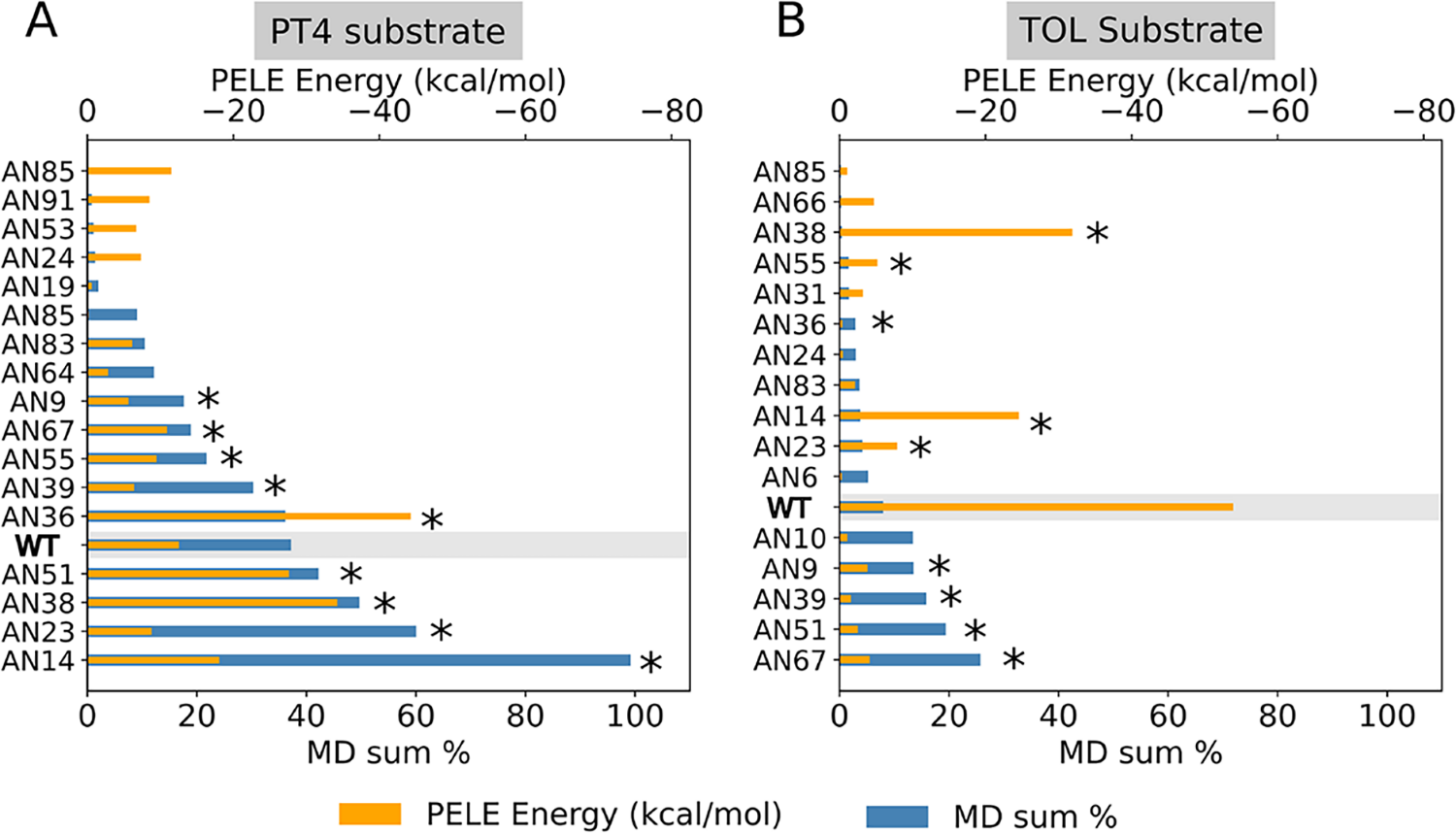
Summary of PELE interaction energies and MD results for Lip_MRD9_ variants generated through AsiteDesign. The wild-type Lip_MRD9_ is highlighted in gray. Bars represent the MD sampling percentage of productive binding poses (blue) and the Boltzmann PELE interaction energy in kcal·mol^–1^ (orange) for each enzyme variant. The asterisks indicate designs sent to experimental validation. (A) PT4 substrate, (B) TOL substrate. More detailed information is presented in Supporting Tables 2 and 1, respectively.

Substrate binding was further evaluated with the Protein Energy Landscape Exploration (PELE) software,^27^ a Monte Carlo procedure combining substrate perturbation with protein structure prediction algorithms. Substrate binding was monitored by computing NACs (see the Materials and Methods section) and the absence of low-energy noncatalytic configurations whose binding energies fell below the minimum catalytic binding energy. For each model, Boltzmann-weighted probabilities and energetics were computed to distinguish catalytically competent from nonproductive poses (see the Materials and Methods section). This rigorous selection reduced the initial pool to 28 candidates.

For these prioritized variants, all-atom MD simulations were conducted to assess further the persistence of catalytically compatible configurations and the dynamic stability of the enzyme–substrate complex. MD trajectories were analyzed to quantify the temporal prevalence of the catalytic conformations. Only those mutants exhibiting the highest temporal occupancy of productive conformations for PT4 substrate combined with low Boltzmann-weighted PELE binding energy were advanced for experimental validation, selecting a total of 9 variants (Figure 1).

### Rational Design Mutants for PETase Activity

Our second strategy involved a targeted rational design approach. Our rationale focused on two principal criteria: (i) identifying residues spatially adjacent to the catalytic triad whose physicochemical properties could modulate substrate orientation, and (ii) targeting positions with high mutation frequency in the AsiteDesign simulations, indicative of their potential contribution to active-site flexibility and substrate accommodation but, instead of the AsiteDesign mutation, introducing a polar or aromatic residue to explore complementary interaction modes with the PET monomers (Supporting Figure 2).

Initially, three residues, L108, I12, and M78, were selected based on their proximity to conserved catalytic residues and their role in shaping the hydrophobic environment of the active site. We discarded positions that were already tested with Asitedesign but showing nonsatisfactory experimental results, and we added other positions belonging to PETase binding subsites I and II (Figure 2B,C,D) analogous to LCC (Leaf and branch compost cutinase, a well-characterized PET-degrading enzyme) subsites positions. These substitutions were designed to reinforce hydrophobic contacts and favor aromatic stacking interactions with the PET chain, while also supplying polar groups to strengthen the hydrogen-bonding network interactions with the substrate. Such interactions were compromised because AsiteDesign tendency to mutate to small hydrophobic amino acids (e.g., Gly, Ala) potentially expands the active-site cavity; we hypothesized that incorporating polar residues capable of establishing favorable interactions with the catalytic residues and the terephthalate moiety could enhance binding and stabilization of the pocket. Conversely, at positions belonging to PETase subsites (e.g., G11, A105, I135, L160), we introduced targeted mutations to tyrosine, serine, asparagine, and phenylalanine, resulting in an extended set of rationally designed variants.

**Figure 2:**
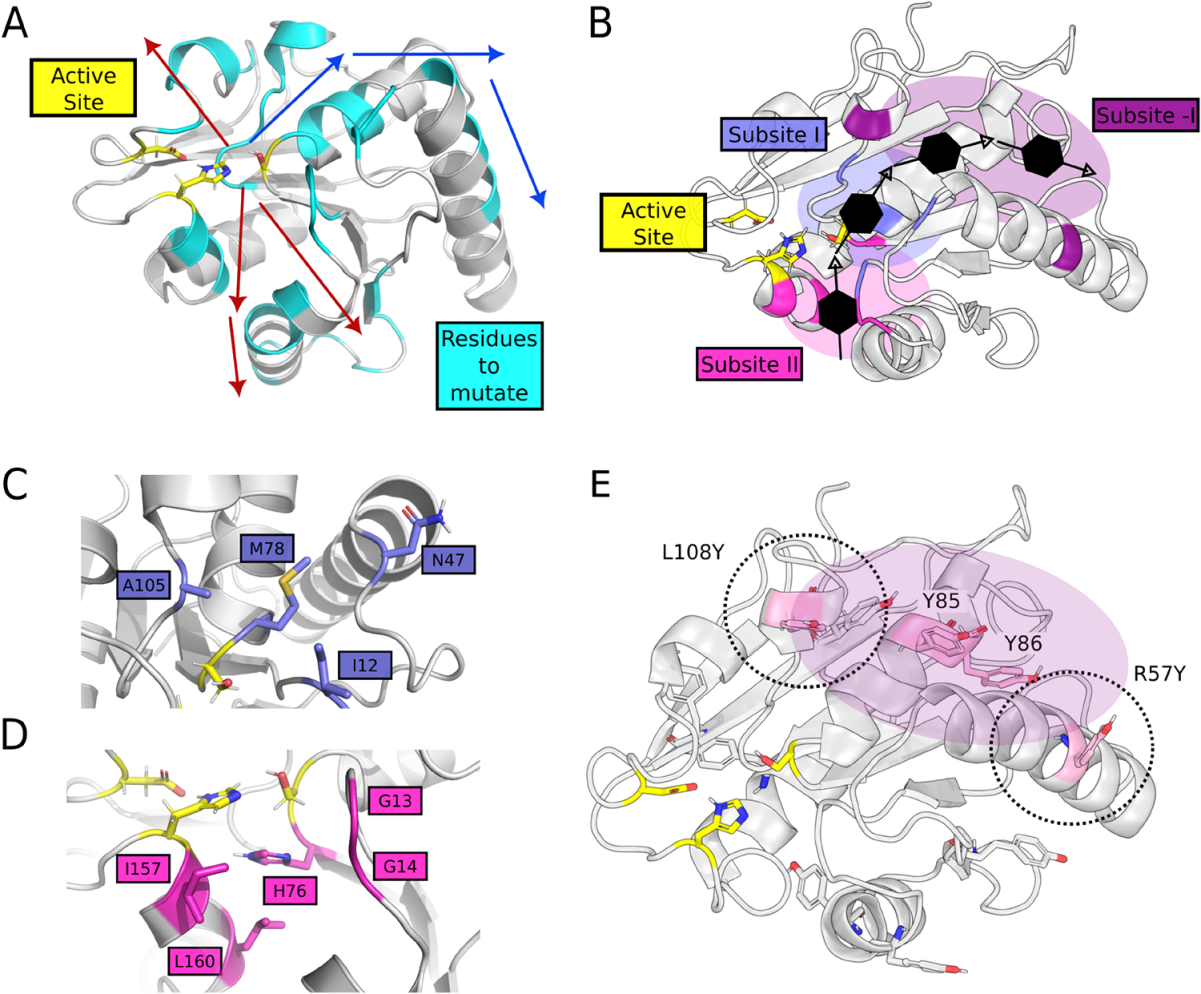
Structural definition of mutational targets and subsites in Lip_MRD9_. (A) Residues selected for AsiteDesign mutagenesis (cyan) are mapped around the active site (yellow sticks), based on proximity and potential substrate long-range interactions. (B) Mapping of substrate-binding subsites in Lip_MRD9_. The black hexagons and arrows represent a suggested path of a PET fragment progressing through the subsite -I (purple) beyond the active site. This structural map facilitates the rationalization of which residues may be mutated to enhance binding. (C) Rotated zoom into Subsite I, showing key residues (N47, I12, M78, A105). (D) Rotated Zoom into Subsite II, highlighting residues (G13, G14, H76, I157, L160) shaping the opposite flank of the pocket. (E) Structural representation of the mutant RA13 (L108Y) double mutant RA18 (R57Y; L108Y). The mutations contribute to the formation of a continuous path of tyrosines together with Y85 and Y86 (highlighted in pink).

Several surface mutations were rationally introduced, aiming at increasing PET recognition and stabilization. The mutation L108Y (RA13) was rationally introduced to extend the aromatic surface at the entrance of subsite -I, creating additional opportunities for π–π interactions with the PET polymer, thereby extending the substrate-binding interface beyond the canonical binding pocket. In addition, previous studies have demonstrated that partial reduction of the electrostatic surface potential, for example by replacing arginine with less polar residues, can facilitate PET accommodation along the protein surface.^33,34^ Thus, we designed the R57Y mutation (RA18) (Figure 2E, circled, right) that simultaneously reduces the local positive charge by replacing arginine with tyrosine and contributes to the formation of a continuous tyrosine track together with wild-type Y85 and Y86 (Figure 2E, highlighted in pink). This arrangement is hypothesized to facilitate extended PET polymer binding beyond the catalytic cleft along subsite -I by providing an aromatic platform for stabilizing the substrate backbone.

Finally, we designed another mutant, RA19 (R47N; K86C; R110 K; L112C), combining substitutions of positive residues (R47 and R110) with less charged polar residues (Asn and Lys, respectively) and introduced cysteine mutations at positions K86 and L112 to promote loop stabilization via disulfide bond formation in subsite -I. This design aimed to reinforce the rigidity of the loop lining subsite -I and modulate the electrostatic landscape to better match the extended PET polymer at the same time. Although both Arg and Lys are positively charged, Lys was chosen to slightly reduce the side-chain length and electrostatic potential without completely removing the positive charge, aiming to moderate the interaction with PET’s carboxyl groups.

Similarly to the AsiteDesign mutants, Boltzmann-weighted probabilities and energetics binding were monitored by computing NACs through PELE simulations. In addition, all-atom MD simulations were conducted to assess the persistence of catalytically compatible configurations and the dynamic stability of the enzyme–substrate complex. Only those mutants exhibiting the highest temporal occupancy of productive conformations combined with low Boltzmann-weighted PELE binding energy were advanced for experimental validation (8 out of 15; see Figure 3). A detailed summary of designing criteria is provided in Table 3, while the catalytic distance distributions and RMSD metrics derived from MD simulations are illustrated in Supporting Figures 1 and 3.

**Figure 3:**
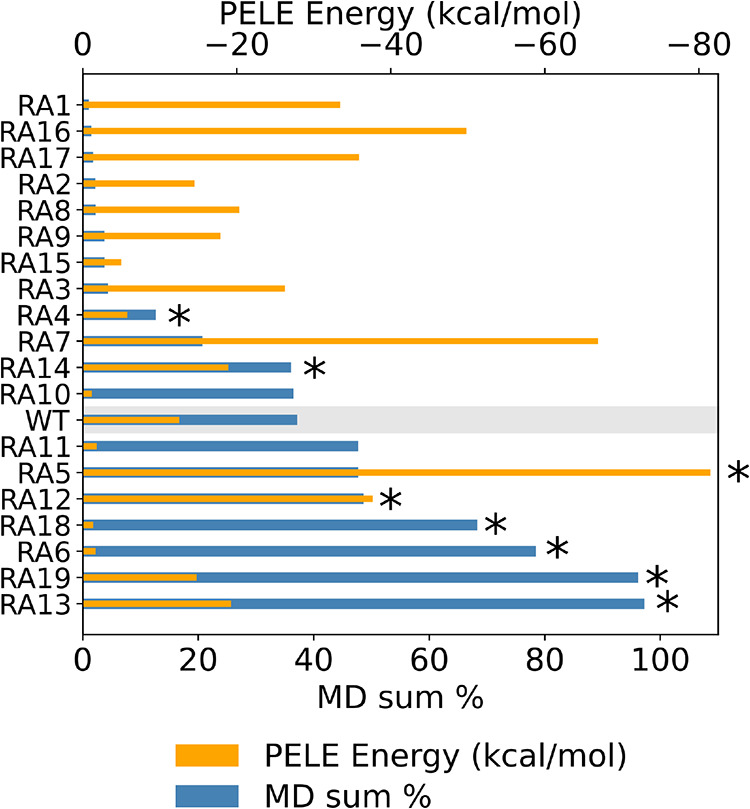
Summary of PELE energy and MD results for Lip_MRD9_ variants rationally generated. The wild-type Lip_MRD9_ is highlighted in gray. Bars represent the MD sampling percentage of productive binding poses (blue) and the Boltzmann PELE interaction energy in kcal·mol^–1^ (orange) for each enzyme variant. The asterisks indicate designs sent to experimental validation. More detailed information is presented in Supporting Table 3.

MD simulations of these last variants indicated that the introduction of tyrosine residues effectively increased the residence time of PET oligomers along the enzyme surface. The Ser-His (blue) and His-acid (orange) distances remain stable within catalytically competent ranges (<4.5 Å), indicating maintenance of the proton relay geometry throughout the trajectory (Figure 4A). When comparing the distances separately, the Ser-Lig distance shows a clear shift toward shorter values in the single and double mutant relative to wild-type. The double mutant RA18 (L108Y; R57Y) shows increased sampling (∼68%) compared to wild-type (∼45%), consistent with improved substrate accommodation, although less than the single mutant RA13 (L108Y) (∼73%) (Figure 4B). For RA18 mutant, whose second mutation is far from the active site, we further simulate PT4 in a starting position far from the active site during 1 μs, the Ser-Lig distance (green) decreases over the first 300 ns, demonstrating substrate diffusion into the active-site cleft (Figure 4C,D) in two out of three replicas (Supporting Figure 11).

**Figure 4:**
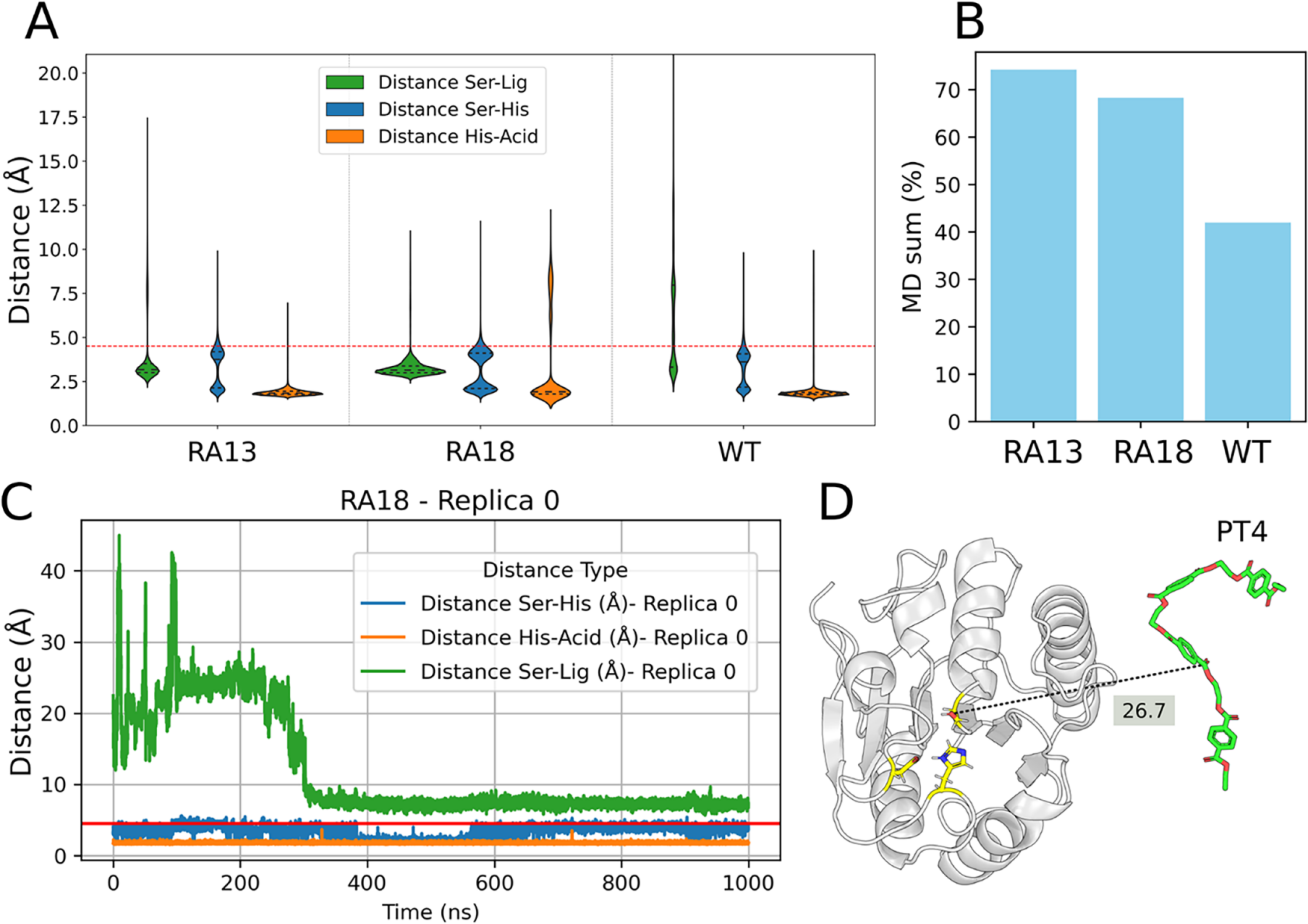
MD simulations and catalytic geometry analysis for wild-type and RA13 and RA18 variants with PT4. (A) Violin plots depicting the distributions of the three key distances (Ser-Lig, Ser-His, His-acid) aggregated over all replicas. The dashed red line marks the 4.5 Å threshold indicative of productive binding configurations. (B) Cumulative sampling percentage of catalytically competent binding poses (MD sum %, all catalytic distances lower than 4.5 Å) across the three 1 μs replicas. (C) Time evolution of key geometric distances during a 1 μs MD trajectory of the double mutant RA18 (L108Y; R57Y) initiated from a configuration with the substrate distal to the active site. (D) Initial position of the substrate for the MD simulation.

To elucidate the impact of surface and subsite mutations on substrate accommodation, we analyzed MD simulations comparing the wild-type enzyme and the disulfide bond-containing mutant RA19. The reduced RMSF observed in the engineered variant supports the stabilizing effect of the disulfide bond and suggests that combined electrostatic and covalent modifications can enhance local structural rigidity while facilitating polymer binding (Supporting Figure 5).

These observations support the hypothesis that strategic surface redesign, targeting both charge modulation and aromatic residue incorporation, can substantially expand the functional binding interface and enhance polymer affinity.

### Homologous Sequences of Lip_MRD9_

Lip_MRD9_ is highly similar to the *Bacillus pumilus* poly­(butylene adipate-*co*-terephthalate) (PBAT) hydrolase (DB Hit ID 00179; DDBJ accession LC189557; 96.7% identity; *E*-value 2.0e–126) and to its closest homologous lipase, LipA, from the *Bacillus* genus (PDB: 7R1K, 95% identity), but shows only 16% sequence identity to benchmark PETases such as *Is*PETase and LCC_*WT*_. We confirmed that both PBAT_*BP*_ and LipA degraded nPET_GFa_, although at much lower levels (66 ± 6 and 44 ± 7 μM degradation products, respectively, after 24 h at 30 °C) than Lip_MRD9_ (1073 ± 5 μM degradation products after 24 h).^7^ Subsequently, we searched for homologous proteins to Lip_MRD9_ using DIAMOND BLAST with default parameters, to evaluate their potential dual lipase and PETase character. Searches were first performed in the Marine Metagenomics databases (MarRef and MarDB), from which Lip_MRD9_ was identified initially,^7^ and were then extended to the NCBI nonredundant protein sequence (nr) and UniProtKB (Swiss-Prot and TrEMBL) databases. Homologues were selected according to stringent cutoff criteria (percent identity >70% and alignment length >190 amino acids out of the 215 in Lip_MRD9_). A total of 70 unique nonredundant sequences were retrieved (Supporting Data 1), and when Lip_MRD9_ was aligned with the remaining members, similarity scores ranged from 71.03% to 95.81%. The mean similarity score was 87.41%, with an interquartile range (IQR) of 3.49%, reflecting an overall high level of similarity within the cluster (Supporting Figure 9). All homologous proteins were assigned to a bacterium of the *Bacillus* genus.

Homologue candidates were first ranked based on substrate-binding energies estimated using PELE, incorporating Boltzmann-weighted probabilities for poses that satisfied structural constraints near the catalytic site. Additionally, MD simulations were employed to evaluate substrate residence times. Based on this computational prescreening, six homologous sequences were selected for experimental validation (Figure 5).

**Figure 5:**
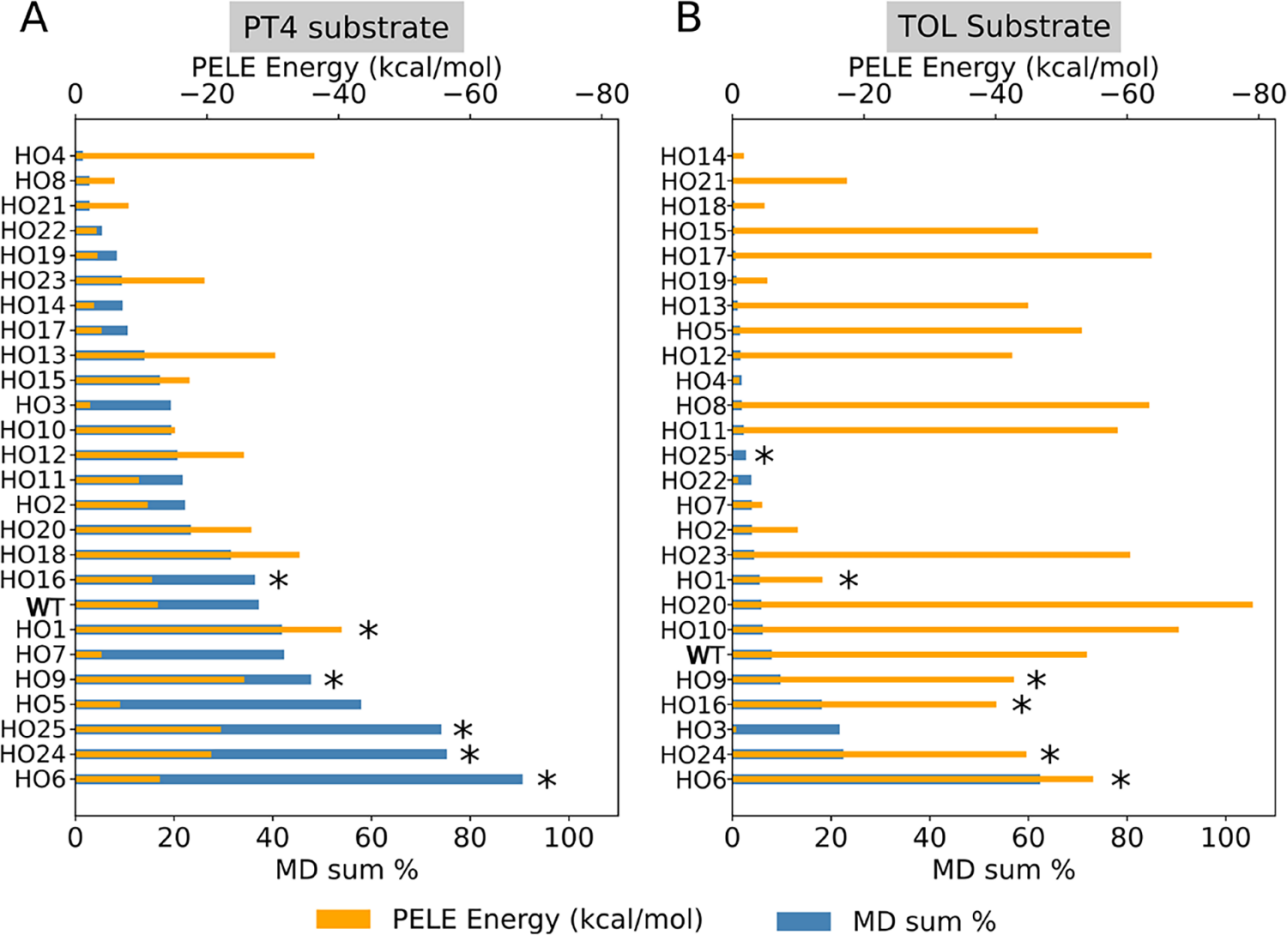
Summary of PELE energy and MD Results for Lip_MRD9_ homologue sequences. The wild-type Lip_MRD9_ is highlighted in gray. Bars represent the MD sampling percentage of productive binding poses (blue) and the Boltzmann PELE interaction energy in kilocalories per mole (orange) for each enzyme variant. The asterisks indicate designs that were sent to experimental validation. (A) PET tetramer substrate, (B) TOL substrate. More detailed information is presented in Supporting Tables 6 and 5, respectively.

Structurally, the six natural variants share a conserved α/β-hydrolase fold; small but potentially functionally relevant differences among the six variants are summarized in Supporting Table 8, with structural locations of divergent residues mapped in Supporting Figure 8.

### Selection and Production of Variants and Homologues

Variants derived from three complementary approaches to enhance dual lipase–PETase activity in Lip_MRD9_, (i) automated *in silico* engineering (AN), (ii) structure-guided rational engineering (RA), and (iii) homology-modeling bioprospecting of novel enzymes (HO), were selected to enable direct comparison of the strategies. The amino acid sequences encoding a total of 23 variants, including 9 AN mutants (AN9, AN14, AN23, AN36, AN38, AN39, AN51, AN55, AN67), 8 RA mutants (RA4, RA5, RA6, RA12, RA13, RA14, RA18, RA19), and 6 homologues (HO1, HO6, HO9, HO16, HO24, HO25), were used as templates for gene synthesis. The sequences were synthesized by GenScript Biotech (Rijswijk, The Netherlands) and codon-optimized to maximize expression in *E. coli*. Each construct was individually inserted into a pET-45b­(+) expression vector carrying an ampicillin resistance marker, with protein expression induced by IPTG. The resulting plasmids were transformed into *E. coli* BL21 (DE3) (ref 200131; Agilent Technologies, CA, USA), and the soluble N-terminal His6-tagged proteins were expressed.

The native Lip_MRD9_ enzyme was produced at 0.656 mg L^–1^, which served as the reference for comparison with mutants and homologues. Most AN and HO variants displayed lower production levels, except for AN36 (0.698 mg L^–1^) and AN67 (0.686 mg L^–1^), which were slightly higher than those of Lip_MRD9_. Among the RA mutants, RA14 stood out with a production of 1.58 mg L^–1^, representing more than a 2-fold increase compared to the native enzyme. All other RA and HO variants showed intermediate to lower yields (0.226–0.542 mg L^–1^). Overall, these results highlight RA14 as the most promising variant in terms of production efficiency, while AN36 and AN67 also achieved values comparable to or slightly higher than those of Lip_MRD9_.

### Experimental Validation of Variants Engineered or Discovered by Different Methods

Once produced and purified, the enzymes (0.08 mg mL^–1^) were characterized for lipase activity using *p*-nitrophenyl decanoate (*p*NPC10, 1.2 mM), a long-chain substrate efficiently hydrolyzed by the enzyme (Table 1), and for PETase activity using nPET_GFa_ (1.7 mg mL^–1^) prepared as described elsewhere,^7^ which was also effectively degraded (30 °C, 984 ± 20 μM after 21.5 h; 40 °C, 2238 ± 40 μM after 21.5 h). Lipase activity was measured colorimetrically, while PETase activity was quantified by HPLC, both at 40 °C and pH 7.0 in 40 mM 4-(2-hydroxyethyl)-1-piperazineethanesulfonic acid (HEPES) buffer containing 150 mM NaCl, as described elsewhere.^7^ For details, see the Materials and Methods section.

**Table 1:** Substrate Specificity of Lip_MRD9_ Hydrolase toward *p*-Nitrophenyl Esters of Different Chain Lengths.

specific activity (units/mg)[Table-fn fn1]						
pNPC2	pNPC3	pNPC4	pNPC8	pNPC10	pNPC12	pNPC16
**7.2** ± **0.17**	**7.0** ± **0.28**	**5.7** ± **1.0**	**1.5** ± **0.09**	**1.4** ± **0.06**	**0.39** ± **0.29**	**0.029** ± **0.019**

1 Reactions were carried out in 96-well microplates at 40 °C by monitoring absorbance changes at 348 nm. Reaction conditions were: 4 μL substrate at 40 mM (1.6 mM final), 2 μL enzyme at 1 mg mL^–1^ (0.02 mg mL^–1^ final), and 100 μL HEPES buffer (40 mM, pH 7.0, 75 mM NaCl), in a total volume of 106 μL. The release of *p*-nitrophenol was calculated using a molar extinction coefficient ε_348_ = 4,147 mM^–1^ cm^–1^ with path length correction applied. Specific activity is expressed as activity units (μmol of product released per minute) per mg of protein.^7^

Experimental data revealed distinct outcomes depending on the engineering strategy employed. Among the predicted mutants generated via AsiteDesign (variants with AN ID), most substitutions had detrimental effects on lipase and PETase activity to different extents (Figure 6; Supporting Table 7). Only a few variants retained activity levels comparable to the wild-type (WT). In particular, AN23 (F20A; W31F) maintained both lipase and PETase activity, with lipase activity slightly reduced (87% relative to WT) but PETase activity modestly increased (113%). AN36 (L108A) preserved lipase activity (96%) while showing a reduced PETase efficiency (11%). The variant AN67 (I102A) stood out for retaining and even improving lipase activity (133% relative to the WT) at the expense of markedly reduced PETase activity (41%). In line with computational simulations using TOL as a model substrate, this effect can be explained by the replacement of a bulky residue in the active-site cavity with a smaller hydrophobic one, thereby expanding the accessible volume and facilitating the accommodation of bulky triglyceride-like substrates. Experimentally, this translated into increased hydrolysis of *p*NPC10, a bulky ester substrate, but contrasted with the reduced activity observed on PET, which requires a different binding mode. That said, the results show that while some AN variants displayed differential trade-offs between lipase and PETase activity, none of the introduced mutations substantially improved both activities simultaneously.

**Figure 6:**
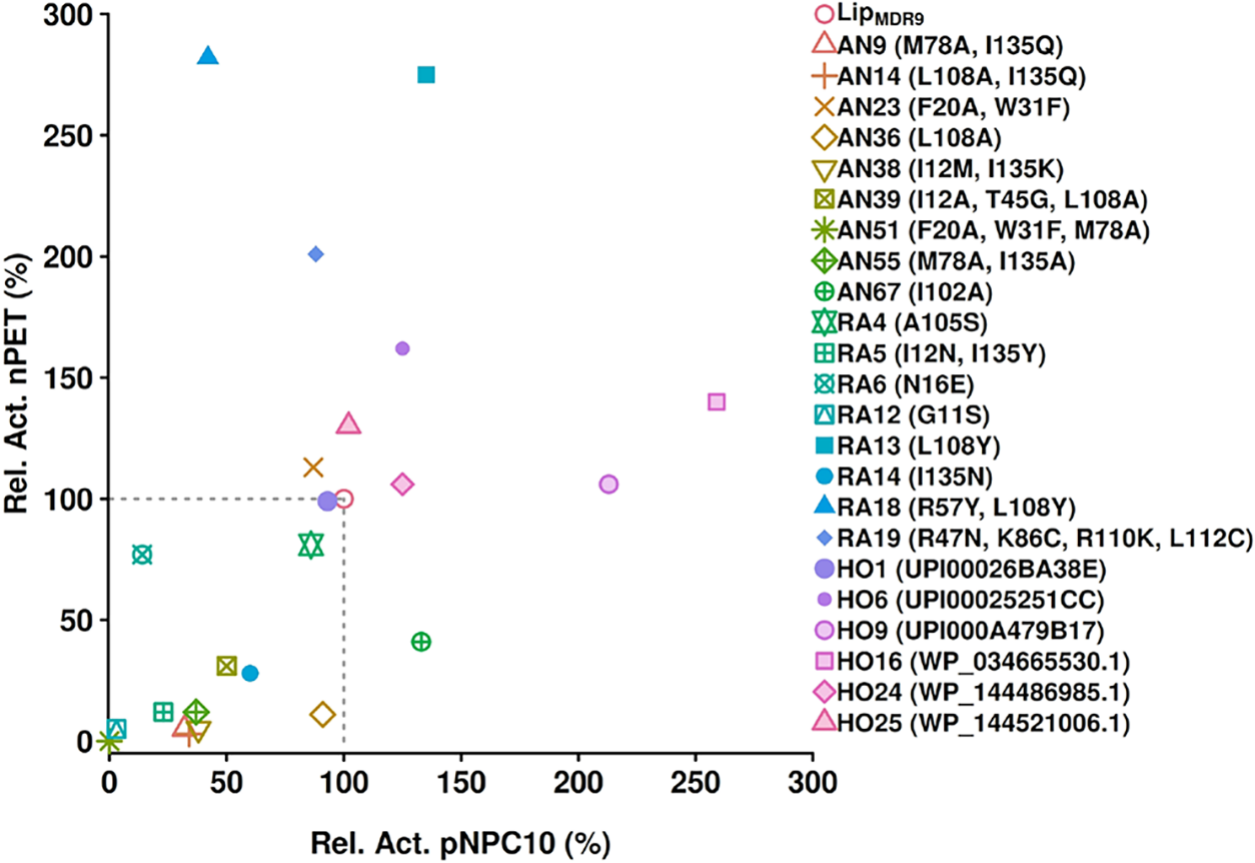
Relative enzymatic activities of Lip_MRD9_ variants and related homologues toward model substrates. Scatter plots comparing the relative hydrolytic activities of Lip_MRD9_, designed mutants, and other homologous enzymes against *p*-nitrophenyl decanoate (pNPC10) (*x*-axis) and nanoPET (nPET_GFa_) (*y*-axis). Activities are expressed as percentages relative to the activity of WT under the same conditions. Each data point represents an individual variant, as indicated by the corresponding colored symbol and label. Several engineered mutants, including RA18 and RA19, display significantly enhanced activity on *p*-nitrophenyl decanoate, suggesting that specific substitutions can selectively improve activity toward PET-related substrates.

Shifting from predictive to structure-guided engineering, rational design targeted residues close to the catalytic pocket (variants with the RA ID). Most variants negatively affected activity on one or both substrates (Figure 6; Supporting Table 7). For example, RA4 (A105S), RA5 (I12N; I135Y), RA6 (N16E), RA12 (G11S), and RA14 (I135N) showed reduced activities on both pNPC10 (14–86%) and PET (5–81%). In contrast, the three variants displayed markedly enhanced PETase activity. RA13 (L108Y) improved both lipase (135%) and PETase (275%) activity compared to the wild-type, preserving dual activity. RA18 (R57Y; L180Y) produced the highest PETase enhancement, with activity 2.8-fold above wild-type (282%), though lipase activity decreased (42%). RA19 (R47N; K86C; R110 K; L112C) also displayed a clear improvement in PETase hydrolysis (201%), while maintaining near-wild-type lipase levels (88%). These three mutants produced a pronounced accumulation of soluble PET oligomers, as confirmed by HPLC, consistent with enhanced catalytic turnover (Figure 6). Overall, rational engineering yielded three promising variants (RA13, RA18, and RA19) with substantially improved PETase activity, while most other designs were detrimental. The structural mapping of mutational effects highlights residues whose substitutions enhanced PET hydrolysis, particularly those located in subsite I (Figure 7).

**Figure 7:**
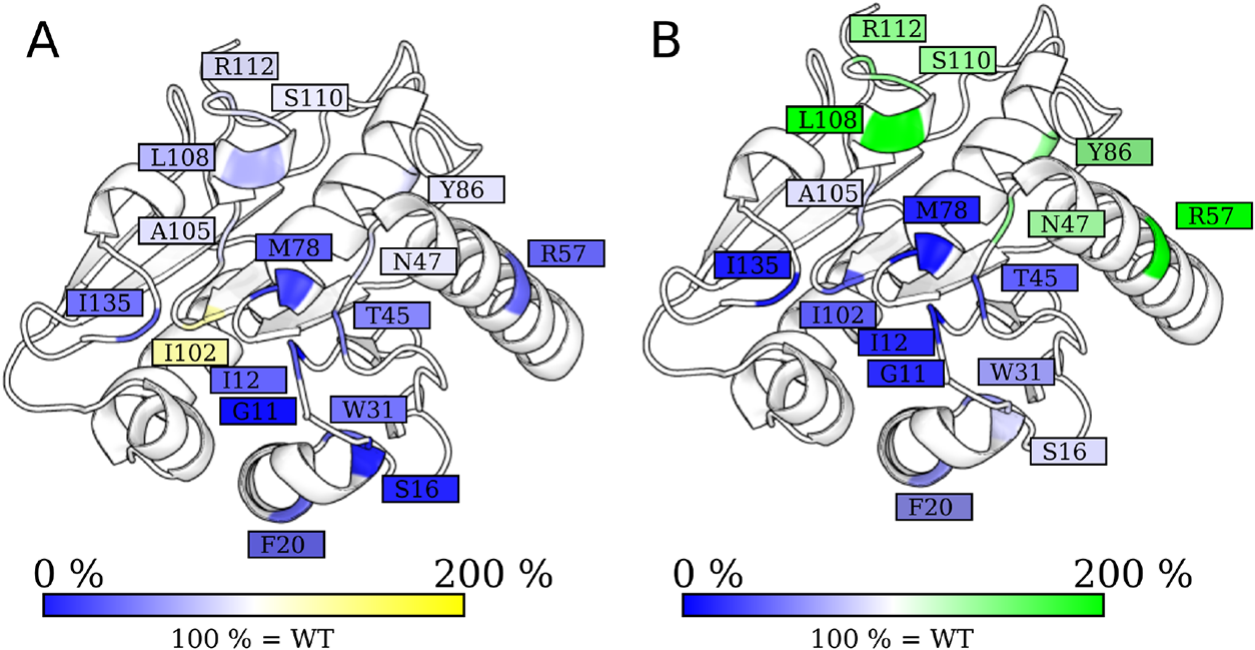
Structural mapping of mutational effects on enzyme activity. (A) Relative activity compared to the wild-type (WT) with the pNPC10 substrate. Mutated residues are colored according to their activity level, with blue indicating reduced activity (<100%) and yellow indicating slightly increased activity (∼100–200%). (B) Structural representation of mutations with improved relative activity with PET compared to WT. Residues are colored in green when showing enhanced activity (>100%). Residues are labeled by their one-letter amino acid code and position.

Finally, bioprospecting through homology modeling identified six natural variants (HO1, HO6, HO9, HO16, HO24, and HO25), which are predicted to overall preserve or enhance enzymatic activity compared to Lip_MRD9_ (Figure 6; Supporting Table 7). The sequence similarity analysis revealed the following scores of homology against Lip_MRD9_: HO9 (99.1%), HO16 (95.4%), HO24 (95.4%), HO25 (95.4%), HO6 (92.1%), and HO1 (72.0%). Supporting Table 8 lists amino acid residues at conserved positions across the aligned sequences, highlighting substitutions that may underlie functional divergence. HO1, HO24, and HO25 exhibited activities comparable to Lip_MRD9_, with lipase activities of 93%, 125%, and 102% and PETase activities of 99, 106, and 130%, respectively. HO6 exhibited a moderate improvement in both activities (125% lipase and 162% PETase). HO9 displayed a marked increase in lipase activity (213%) while maintaining PETase levels similar to Lip_MRD9_ (106%). The most substantial improvements were observed in HO16, which showed the highest lipase activity (259%) together with a substantial increase in PETase activity (140%). Overall, unlike the AN and RA mutants, most homologues maintained or improved dual lipase–PETase activity, with HO16 emerging as the most promising candidate due to its combined enhancement of both functions.

To dissect whether improvements in screening activity reflect changes in turnover, affinity, or both, we determined *k*_cat_, *K*_*m*_, and *k*_cat_/*K*_*m*_ for selected variants on pNPC10 and nPET_GFa_ (Table 2). For pNPC10 hydrolysis, RA19 exhibits the highest catalytic efficiency (2645.5 ± 6.1 min^–1^ mM^–1^). This improvement is primarily affinity-driven, as *K*_*m*_ decreases more than 3-fold compared to Lip_MRD9_ (0.12 vs 0.41 mM), while *k*_cat_ shows only a moderate increase. The substantial reduction in *K*_*m*_ indicates improved substrate binding or positioning within the active site, which translates into the strongest overall performance. AN23 (2453.2 ± 6.0 min^–1^ mM^–1^) follows closely but displays a distinct kinetic profile: the enhancement is mainly turnover-driven, with an approximately 6-fold increase in *k*_cat_ and a slightly reduced *K*_*m*_. This suggests that the mutations in AN23 predominantly optimize the catalytic step rather than the substrate binding. HO9 (2191.4 ± 133.0 min^–1^ mM^–1^) also achieves a marked improvement, largely due to a drastic reduction in *K*_*m*_ (0.06 ± 0.00 mM), despite a slightly lower *k*_cat_ than Lip_MRD9_, reinforcing the importance of improved substrate affinity in boosting lipase efficiency. RA18 (1246.7 ± 5.7 min^–1^ mM^–1^) represents a clear example of *k*_cat_-driven enhancement. Although *K*_*m*_ increases relative to Lip_MRD9_, the exceptionally high turnover number compensates for the reduced affinity, indicating an improved catalytic chemistry or transition-state stabilization. HO16 (1118.2 ± 30.1 min^–1^ mM^–1^) shows a more balanced optimization, with moderate improvements in both *K*_*m*_ and *k*_cat_. HO6 (622.8 ± 16.9 min^–1^ mM^–1^) mainly benefits from improved affinity, whereas RA13 (371.8 ± 9.3 min^–1^ mM^–1^) displays only a marginal increase over Lip_MRD9_, as its higher *k*_cat_ is largely offset by a substantially increased *K*_*m*_.

**Table 2:** Kinetic Parameters of Lip_MRD9_, Its Engineered Variants, and Homologues toward the Lipase Representative Substrate pNPC_10_ and the PETase Representative Substrate nPET_GFa_

	**pNPC10** [Table-fn t2fn1]			**nPET**_GFa_ [Table-fn t2fn2]		
**enzyme**	*K*_*m*_ (mM)	*k*_cat_ (min^–1^)	*k*_cat_/*K*_*m*_ (min^–1^ mM^–1^)	*K*_*m*_ (g L^–1^)	*k*_cat_ (min^–1^)	*k*_cat_/*K*_*m*_ (min^–1^ g^–1^ L)
Lip_MRD9_	0.41 ± 0.01	145.56 ± 1.25	352.9 ± 8.1	0.41 ± 0.01	2.54 ± 0.03	6.2 ± 0.2
AN23	0.36 ± 0.07	874.92 ± 12.58	2453.3 ± 6.0	0.24 ± 0.01	2.18 ± 0.04	9.1 ± 0.5
RA13	0.86 ± 0.02	319.80 ± 3.60	371.8 ± 9.3	0.35 ± 0.01	4.85 ± 0.03	14.0 ± 0.4
RA18	0.99 ± 0.34	1230.32 ± 35.95	1246.7 ± 5.7	0.47 ± 0.01	6.52 ± 0.05	14.0 ± 0.3
RA19	0.12 ± 0.02	308.59 ± 3.62	2645.5 ± 6.1	0.09 ± 0.01	2.36 ± 0.06	24.9 ± 3.2
HO6	0.20 ± 0.01	124.37 ± 0.91	622.8 ± 16.9	0.14 ± 0.00	2.67 ± 0.02	19.5 ± 0.6
HO9	0.06 ± 0.00	123.55 ± 0.84	2191.4 ± 133.0	0.07 ± 0.01	1.63 ± 0.03	24.6 ± 4.4
HO16	0.24 ± 0.01	264.32 ± 2.05	1118.2 ± 30.1	0.38 ± 0.01	2.81 ± 0.04	7.3 ± 0.3

1 Kinetic parameters (*K*_*m*_ and *k*_cat_) for pNPC10 were measured at 40 °C in 40 mM HEPES, 75 mM NaCl, pH 7.0, using 0.02 mg mL^–1^ enzyme and substrate concentrations 0–3 mM in 100 μL. 4-Nitrophenol release was monitored at 348 nm in 96-well plates; initial rates were fitted to the Michaelis–Menten model. All reactions were performed in triplicate (*n* = 3).

2 Kinetic parameters (*K*_*m*_ and *k*_cat_) for PET nanoparticles (nPET_GFa_) were measured at 40 °C in 20 mM HEPES, 75 mM NaCl, pH 7.0, using 0.1 mg mL^–1^ enzyme and 0–0.9 g L^–1^ substrate in 50 μL. Reactions were performed in triplicate (*n* = 3) and hydrolysis products quantified by HPLC; initial rates were fitted to the Michaelis–Menten model.

A similar analysis for nPET_GFa_ hydrolysis reveals that RA19 (24.9 ± 3.2 L min^–1^ g^–1^) and HO9 (24.6 ± 4.4 L min^–1^ g^–1^) are the most efficient variants. In both cases, the improvement is strongly affinity-driven, with *K*_*m*_ values reduced approximately 4- to 6-fold relative to Lip_MDR9_, while *k*_cat_ values remain similar. These results suggest that improved interaction with the PET-like substrate is the main determinant of enhanced PETase activity. HO6 (19.5 ± 0.6 L min^–1^ g^–1^) also follows this trend, showing a substantial reduction in *K*_*m*_ but also an increase in *k*_cat_. In contrast, RA13 (14.0 ± 0.4 L min^–1^ g^–1^) and RA18 (14.0 ± 0.3 L min^–1^ g^–1^) display improvements primarily driven by increased turnover numbers, indicating that acceleration of the catalytic step can also significantly enhance PET hydrolysis even without major gains in substrate affinity in the case of RA18. AN23 and HO16 show more modest improvements (9.1 ± 0.5 and 7.3 ± 0.2 L min^–1^ g^–1^, respectively).

## Discussion

While prior PET depolymerization efforts have often highlighted benchmark enzymes such as *Is*PETase and LCC, it is now evident that the landscape is far broader. Native and mutant PETases are widespread: for example, a recent global assessment identified 1,598 *Is*PETase-like genes in marine environments,^35^ while other large-scale surveys uncovered 75 representative *Is*PETase-like candidates across 83 million gene clusters,^36^ and over 436,000 nonredundant PETase-like sequences with structural similarity to known enzymes.^37^ In parallel, engineered variants have been developed with catalytic performances approaching those required for effective plastic waste biodegradation and recycling, including LCC-ICCG,^11^ HotPETase,^38^ and TurboPETase.^39^ These advances have been facilitated by progress in bioinformatics, machine learning, protein language models, and computational redesign strategies,^36,37,40–42^ as well as mechanistic insights from QM/MM simulations^43^ that guide the design of high-performance variants under conditions relevant for large-scale hydrolysis.^44^ Together, these findings underscore that beyond *Is*PETase and LCC, a vast and diverse repertoire of native and engineered specialized PETases exists.

In this context, our work highlights that lipase-derived scaffolds with inherent PETase activity, such as Lip_MRD9_, can expand this repertoire, supported by their intrinsic catalytic capabilities and by targeted engineering.^15,16,45^ We have attempted three different approaches to improve the hydrolytic performance of a lipase, Lip_MRD9_, which is capable of efficiently hydrolyzing both triglycerides and PET.^7^ Taking Lip_MRD9_ as a reference, we performed automatic *in silico* engineering with AsiteDesign, structure-guided rational engineering, and homology modeling to modulate the inherent lipase and PETase activities of this hydrolase. The rationale behind this approach is that we previously found that a single residue, I12 (according to Lip_MRD9_ numbering), contributes to its PETase activity, suggesting that additional residues may also play a role in influencing the selectivity between lipase and PETase functions. Our results demonstrate that integrating Monte Carlo and MD simulations accounting for NACs provides a good computational framework for the rational preselection of enzymes before experimental testing.

The AsiteDesign-guided mutagenesis of Lip_MRD9_ variants uncovered pronounced mutational hotspots near residues 12, 78, 108, and 157, revealing a high degree of active-site plasticity that can be leveraged to reshape the substrate specificity. This observation aligns with previous reports indicating that small apolar substitutions can alleviate steric constraints by enlarging the substrate cavity.^46^ However, excessive incorporation of alanine or glycine can compromise lipase activity and reduce expression yields, emphasizing the importance of balancing mutational breadth with the preservation of essential structural features. Moreover, the AsiteDesign objective function prioritized local substrate accommodation/catalytic geometry without incorporating (i) evolutionary constraints that reflect what substitutions are tolerated in the Lip_MRD9_ scaffold and (ii) explicit stability and expression-related constraints. Consequently, several designs may satisfy the intended structural criteria *in silico* yet fail experimentally due to impaired folding, reduced soluble expression, or subtle perturbations of the native lipase environment. Future automated campaigns should therefore adopt multiobjective design, combining catalytic/pocket objectives with sequence-plausibility priors (e.g., conservation/coevolution or protein language-model likelihood filters) and stability terms (e.g., *Δ*Δ*G* filtering or Rosetta stability scoring), while explicitly penalizing disruption of the native catalytic machinery.

Among the rationally designed substitutions, mutations targeting subsite -I and the enzyme surface proved to be particularly impactful. For example, the L108Y substitution (in RA13 variant) increased PET hydrolysis 2.75-fold, with MD simulations confirming that the introduced tyrosine engaged in persistent π-π stacking interactions with PET oligomers while maintaining catalytic triad geometry. Notably, this variant preserved high pNPC10 hydrolysis activity, supporting the idea that targeting distal subsites can selectively tune polymer affinity without compromising lipolytic versatility.^47^

Surface remodeling strategies further enhanced the PET depolymerization. Thus, construct RA18 (R57Y; L108Y) created an extended aromatic path along the enzyme surface, improving PET retention and oligomer accumulation. This design approach mirrors structural principles established in high-performing PETases, where distal hydrophobic and aromatic residues increase processivity by stabilizing partially hydrolyzed polymer chains.^48^ HPLC data corroborated these structural insights, revealing higher concentrations of soluble oligomers compared to the wild-type enzyme. On the other hand, the stabilization of subsite-I in RA19 (R47N; K86C; R110K; L112C) also increased the related activity of PET degradation. In these simulations, RA19 displayed reduced RMSF in the engineered loop region, consistent with a decreased local flexibility and with a geometry compatible with the intended disulfide constraint. However, these fine-grained structural interpretations are derived from simulations initiated from a predicted structural model and are therefore presented as simulation-supported hypotheses rather than definitive proof of disulfide formation or loop rigidification in solution. Importantly, our conclusions are driven by the experimentally measured activity profiles, while simulation results are used to rationalize trends and propose mechanistic explanations.

Our analysis revealed no strict correlation between predicted PELE binding energies and the experimental hydrolysis search. Common to all of the approaches, we have filtered potential candidates using substrate binding rates. For example, RA7 (L160A) exhibited low interaction energies (−66.9 kcal·mol^–1^) but minimal activity, while RA13 (L108Y) had moderate interaction energies but high turnover. This observation highlights the limitation of static scoring functions. It suggests that dynamic contributions, such as processivity, induced fit, and long-range electrostatics, play critical roles not captured by binding energy alone. However, for all successful variants, the MD values and perseverance of NACs are present.

Evaluation of six Lip_MRD9_ homologues finally demonstrated that, overall, the natural variants possess both lipolytic and PETase activities structurally encoded within the Lip_MRD9_ family, reinforcing its suitability as a scaffold for further protein engineering toward bifunctional catalysis. Also, in contrast to the AN and RA mutants, most homologues maintained or even improved dual lipase–PETase activity, with HO16 emerging as the most promising candidate due to its combined enhancement of both functions. This evolutionary plasticity reinforces the suitability of lipase scaffolds as adaptable biocatalysts for processing mixed polyester and lipid waste streams (Table 3).

**Table 3:** Rational Design Rationale for Selected Single and Combinatorial Mutations

**variant**	**mutation**	**reason**
RA4	A105S	position A105 is frequently mutated in AsiteDesign to glycine or alanine. Due to its proximity to the active site, alanine was replaced with a serine to enable potential hydrogen bonding with ester groups of the substrate.
RA5	I12N, I135Y	I12N introduces a polar residue at the entrance of the catalytic cleft, potentially enhancing substrate capture and enabling hydrogen bonding with PET. I135Y replaces a hydrophobic residue with an aromatic tyrosine, enabling π–π stacking interactions with the terephthalate rings and improving substrate binding.
RA6	N16E	introduces a negatively charged residue near the surface, which may establish electrostatic interactions or stabilize the orientation of polar residues at the PET-binding interface.
RA12	G11S	replaces a small, flexible glycine with a polar serine that could engage in hydrogen bonding and help organize a polar network near the active site.
RA13	L108Y	L108 is located near the substrate-binding groove. Substitution by tyrosine introduces an aromatic ring that can interact via π-stacking with the aromatic groups of PET, enhancing substrate stabilization.
RA14	I135N	a frequently targeted position; here, isoleucine is replaced by asparagine to introduce a polar side chain capable of hydrogen bonding with ester groups in PET.
RA18	R57Y, L108Y	building upon the L108Y mutation, R57Y introduces an additional aromatic residue. Together with Y85 and Y86, these form an extended aromatic tunnel, potentially stabilizing PET chains beyond the catalytic site via π–π interactions.
RA19	K86C, L112C R47N, R110 K	the K86C–L112C pair introduces a disulfide bridge conserved in other PETases (e.g., MiPAP19), enhancing thermal stability.^49^ R47N and R110 K reduce the positive surface charge, potentially improving interaction with the partially negative PET surface and decreasing electrostatic repulsion.

## Conclusions

Taken together, these findings establish several key design principles for maintaining multifunctional hydrolases. Subsite -I emerges as a region of PET binding that can be selectively expanded via targeted aromatic substitutions. Using complementary and comparative computational strategies: *in silico* mutagenesis, rational design, and homology-based bioprospecting, we identified variants with markedly improved PET hydrolysis while retaining lipase activity. Key design strategies included aromatic substitutions in subsite -I, surface charge modulation, and loop stabilization, which expanded the polymer affinity and enhanced substrate retention. Comparative analysis with natural homologues further highlights the evolutionary plasticity of this enzyme family and its suitability for multifunctional applications. The computational exploration was extensive, including large-scale mutagenesis, MD simulations, and energetic analyses that provided insight into the balance between substrate binding and catalytic turnover. Experimental evaluation of selected variants confirmed the feasibility of the proposed designs and supported the computational observations.

## Supplementary Material



## Data Availability

All input files required to reproduce the simulations, together with the mutant sequences and plotted data sets, will be made publicly available at Zenodo (DOI: 10.5281/zenodo.17550135). Additional data are provided in the Supporting Information.

## Notes

RMS distribution, structural and dynamic comparison, and structural and energetic characterization of selected variants from MD sampling and PELE simulations with TOL (PDF)
