# Supplementary material for: In Silico Optimization of a Bifunctional Lipase–Polyethylene Terephthalate (PET) Hydrolase for Enhanced PET and Lipid Hydrolysis
Source: J Chem Inf Model. 2026 May 29;66(12):7207–20. doi: 10.1021/acs.jcim.6c00609 (PMC13292204; doi:10.1021/acs.jcim.6c00609)
Supplement: Supplementary file 1 [file ci6c00609_si_001.pdf]

In silico optimization of a Bifunctional Lipase–Polyethylene terephthalate (PET) hydrolase for Enhanced PET and Lipid Hydrolysis

Ana Robles-Martín,[†,‡,‖] Paula Vidal,[¶,‖] Rubén Muñoz-Tafalla,[†,‡] Jose L. Gonzalez-Alfonso,[¶] David Almendral,[¶] Francisco J. Plou,[¶] Manuel Ferrer,[*,¶] Laura Fernandez-Lopez,[*,¶] and Víctor Guallar[*,†,§]

†*Barcelona Supercomputing Center (BSC), 08034, Barcelona, Spain*

‡*PhD program in Biotechnology, Faculty of Pharmacy and Food Sciences, University of Barcelona (UB), 08028 Barcelona, Spain*

¶*Instituto de Catalisis y Petroquimica (ICP), CSIC, 28049 Madrid, Spain*

§*Institució Catalana de Recerca i Estudis Avançats (ICREA), Barcelona 08010, Spain*

‖*These authors contributed equally.*

E-mail: mferrer@icp.csic.es; l.fernandez.lopez@csic.es; victor.guallar@bsc.es

*Corresponding authors: Manuel Ferrer, Laura Fernandez-Lopez and Víctor Guallar.

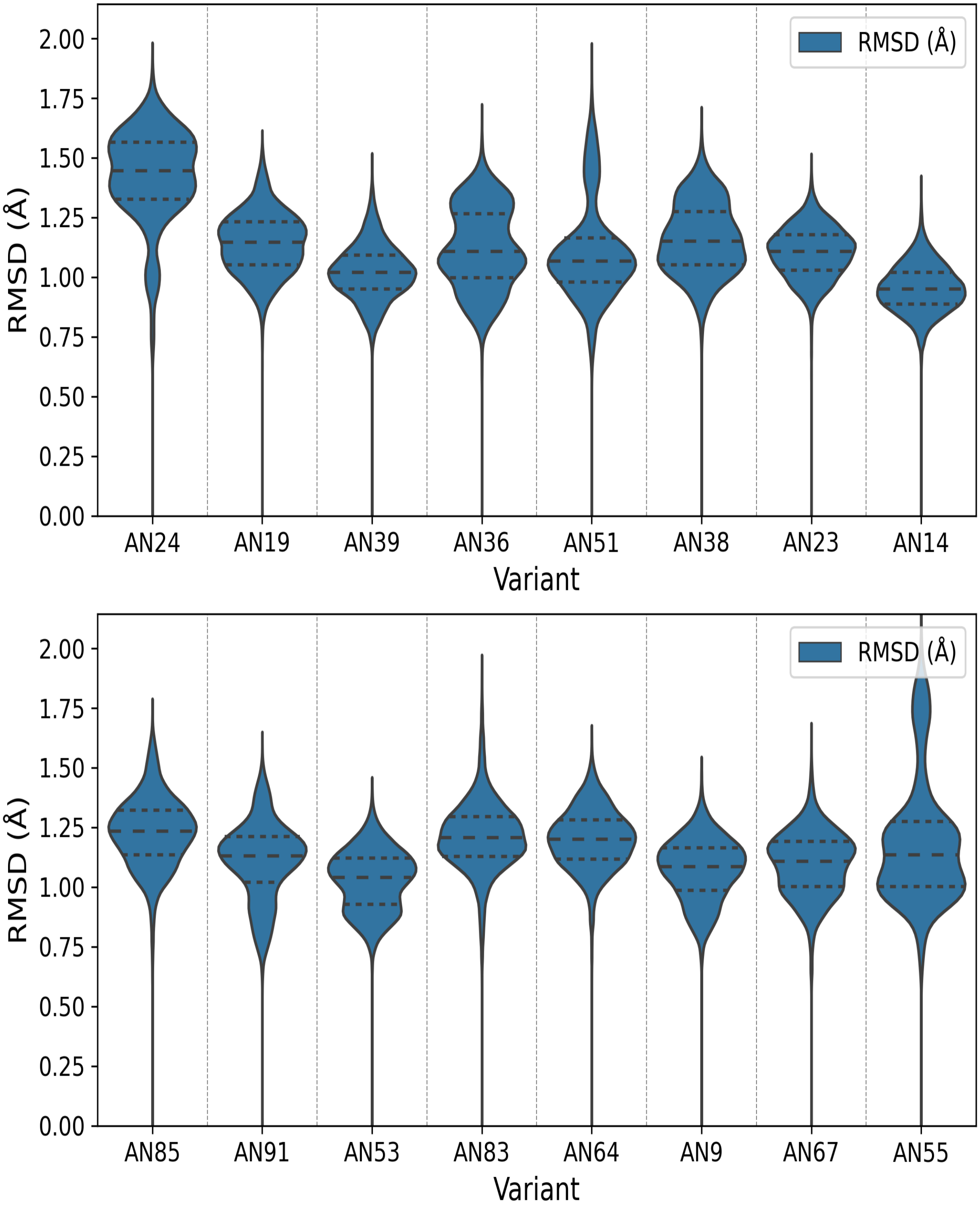

Supplementary Figure 1: Distribution of the root mean square deviation (RMSD) along the 3 replicas of 200 ns of MD simulations for the wildtype and the different Asitedesign variants with PT4 substrate. Boxplots show medians (line), interquartile ranges (boxes), and full ranges (whiskers).

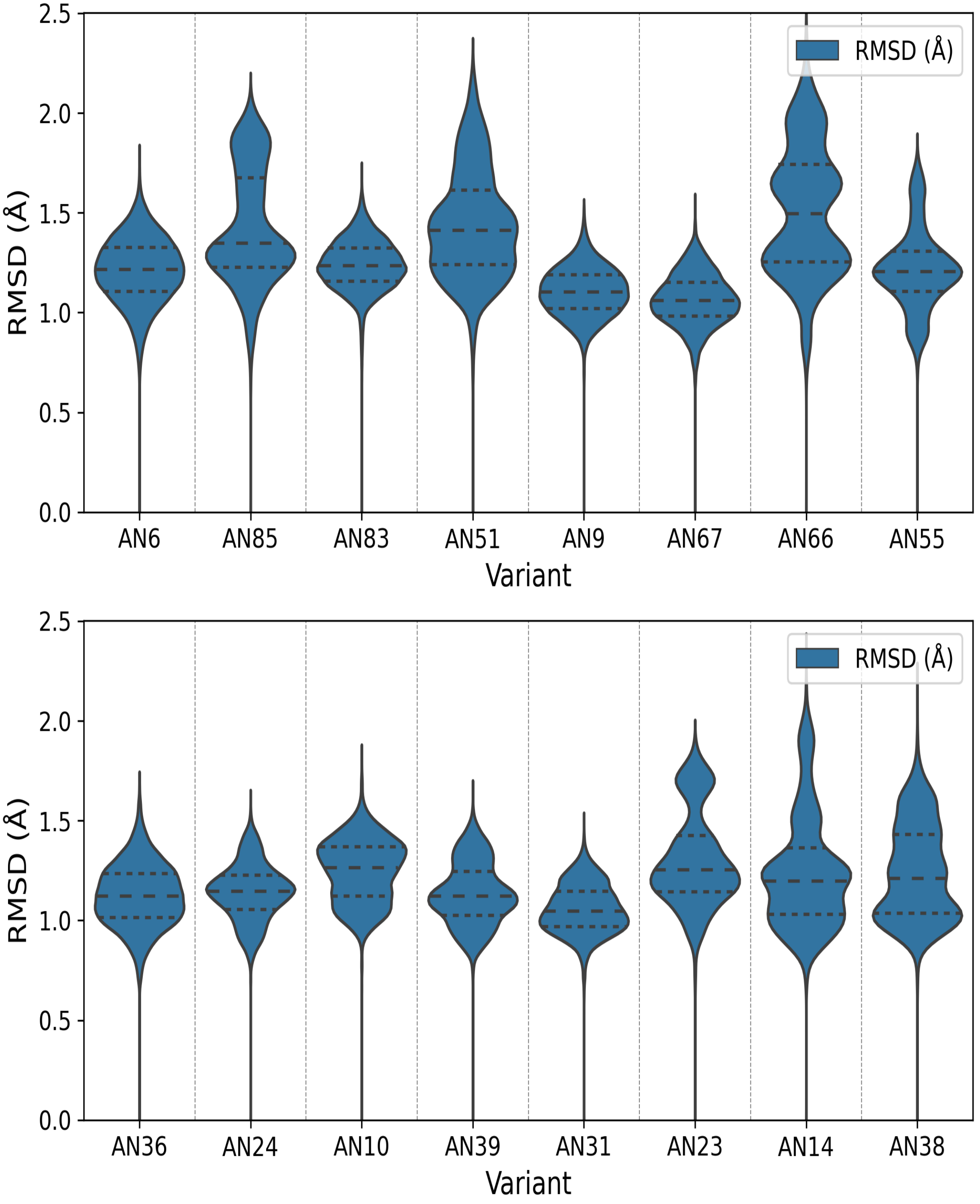

Supplementary Figure 2: Distribution of the root mean square deviation (RMSD) along the 3 replicas of 200 ns of MD simulations for the wildtype and the different Asitedesign variants with TOL substrate. Boxplots show medians (line), interquartile ranges (boxes), and full ranges (whiskers).

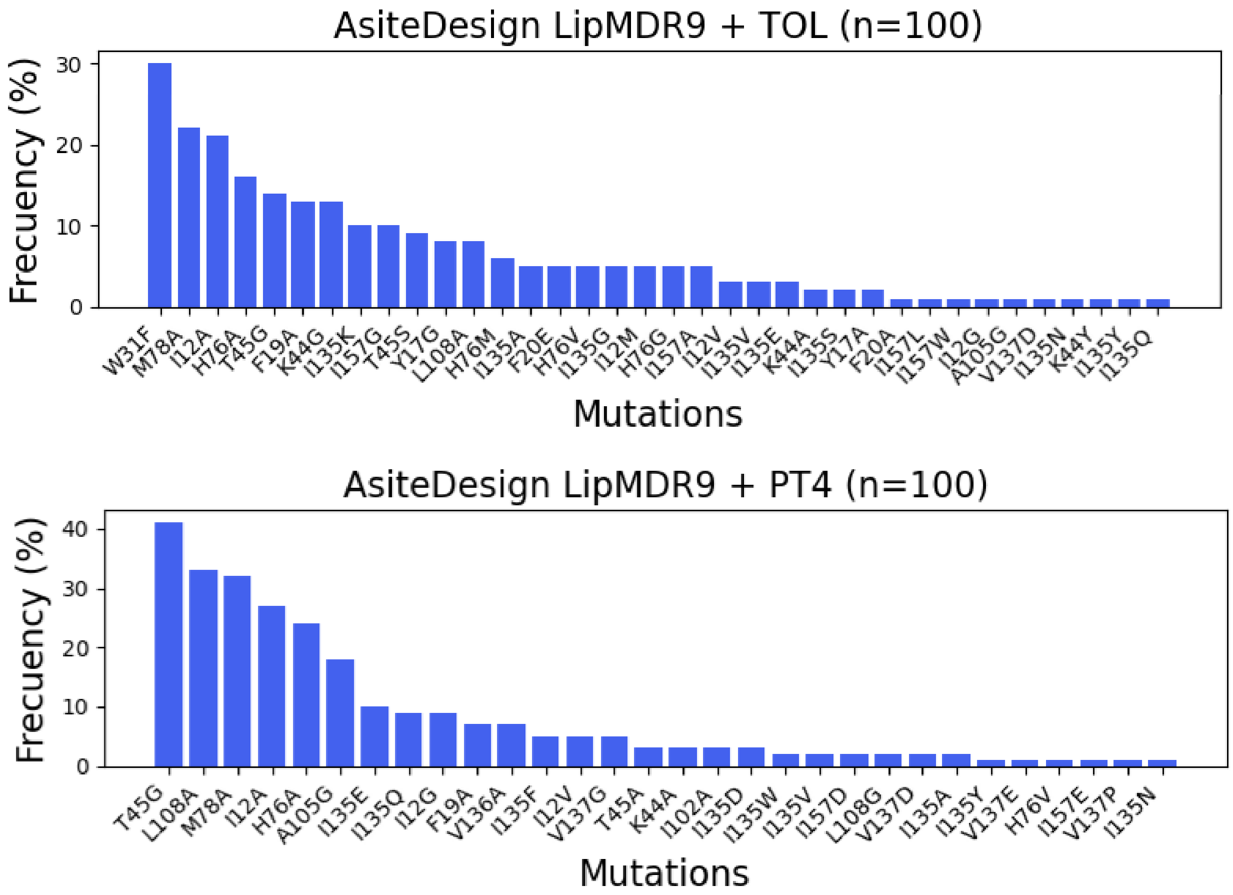

Supplementary Figure 3: Frequency of mutations introduced by *AsiteDesign* in Lip$_{MRD9}$ variants for TOL and PT4 substrates. Bar plots show the distribution of amino acid substitutions observed across 100 designed mutants for each substrate.

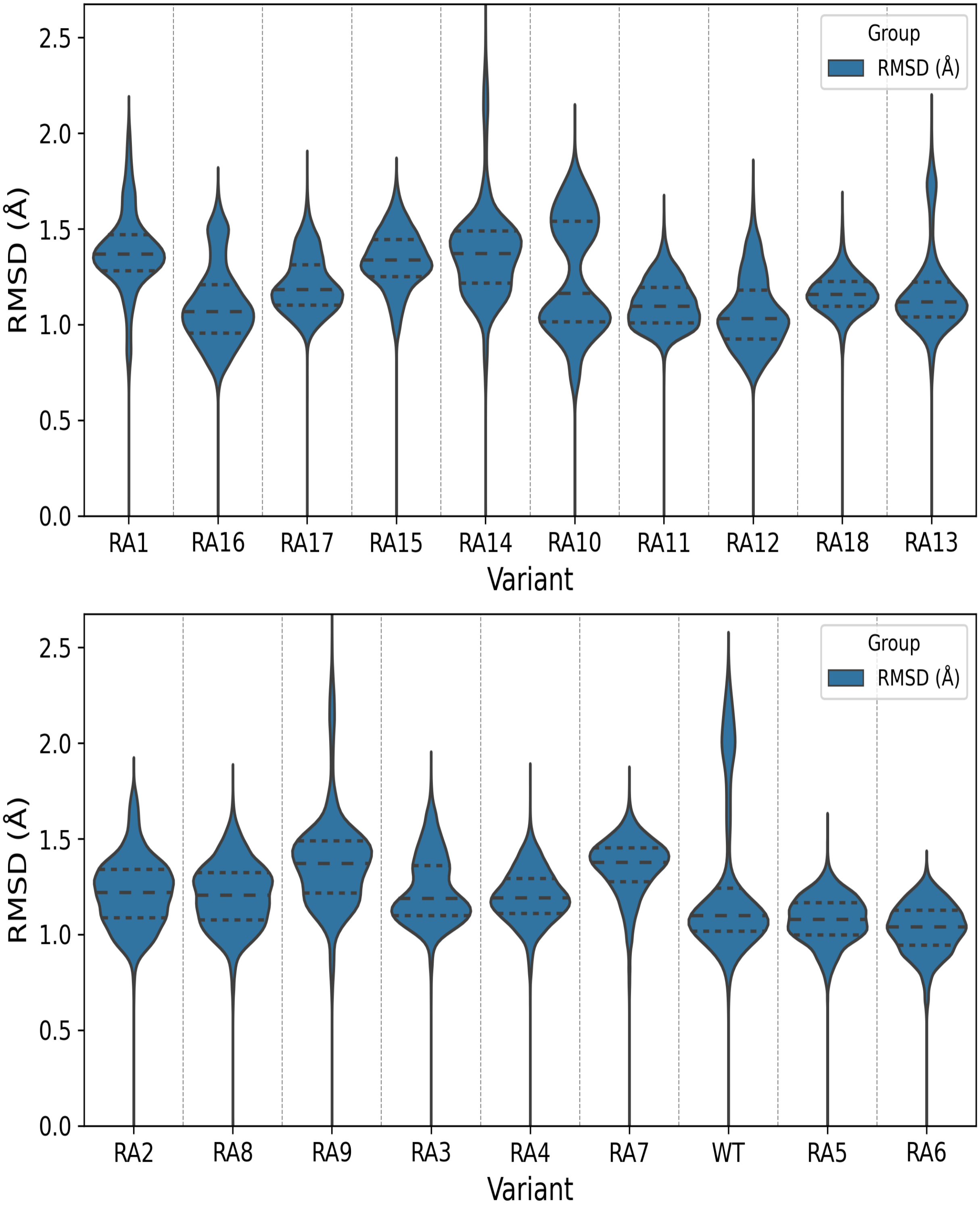

Supplementary Figure 4: Distribution of the root mean square deviation (RMSD) along the 3 replicas of 200 ns of MD simulations for the wildtype and the different rational design variants with PT4 substrate. Boxplots show medians (line), interquartile ranges (boxes), and full ranges (whiskers).

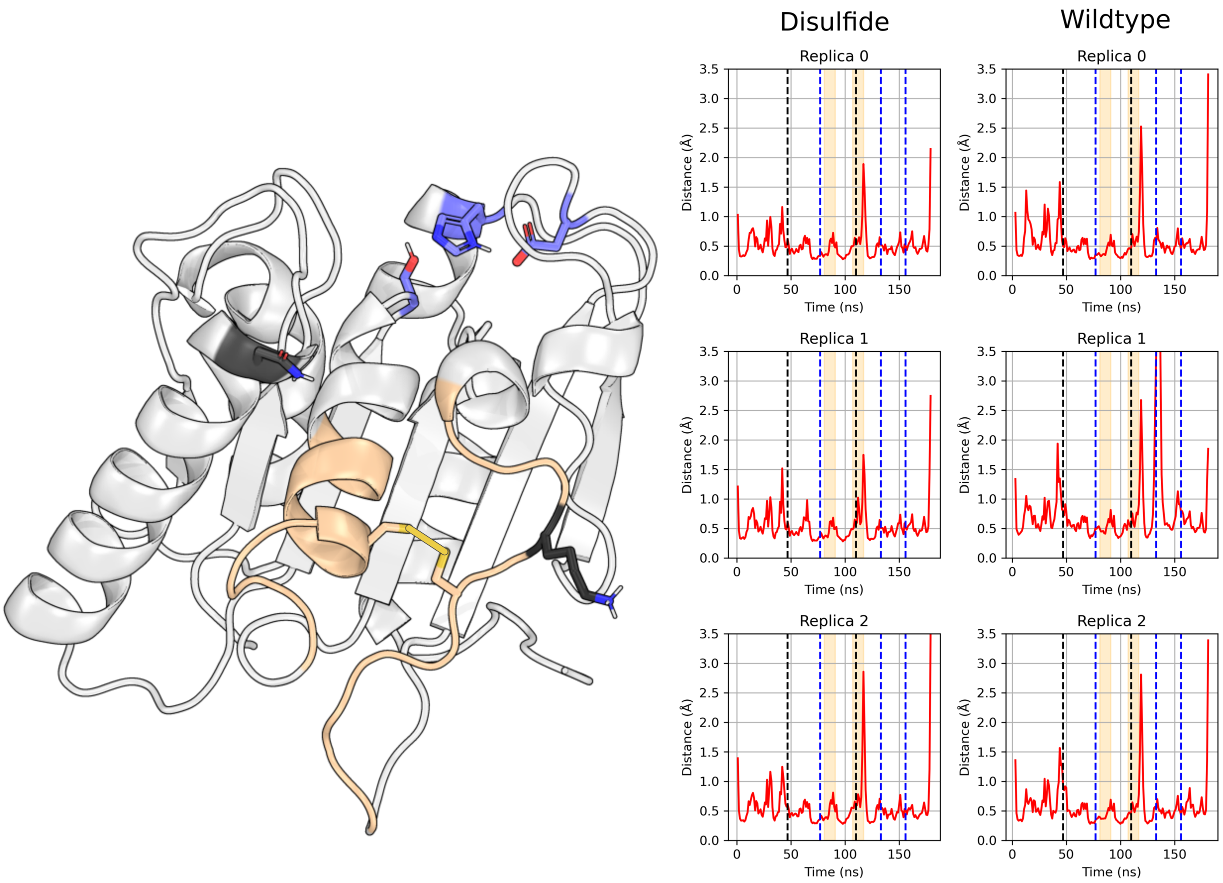

Supplementary Figure 5: Structural and dynamic comparison of the Lip$_{MRD9}$ engineered variant and wildtype. (Left) Representation of the RA19 mutant (R47**N**, K86**C**, R110**K**, L112**C**) highlighting the rationally designed substitutions. Residues R47 and R110, replaced by less positive charged Asn and Lys, respectively (shown in black), were targeted to reduce the electrostatic surface potential and improve substrate accommodation. Cysteine substitutions at positions K86 and L112 (light orange sticks) were introduced to promote disulfide bond formation (yellow linkage) and enhance the rigidity of the loop adjacent to subsite -I. **(Right)** Root-mean-square fluctuation (RMSF) profiles computed from three independent MD replicas for the disulfide-containing mutant (left column) and the wildtype enzyme (right column). The red traces represent per-residue atomic fluctuations over the simulation time (0–200 ns). Vertical dashed lines (black and blue) indicate mutant residues and the active site, respectively, and the shaded beige region denotes the residues window surrounding the disulfide bond analyses.

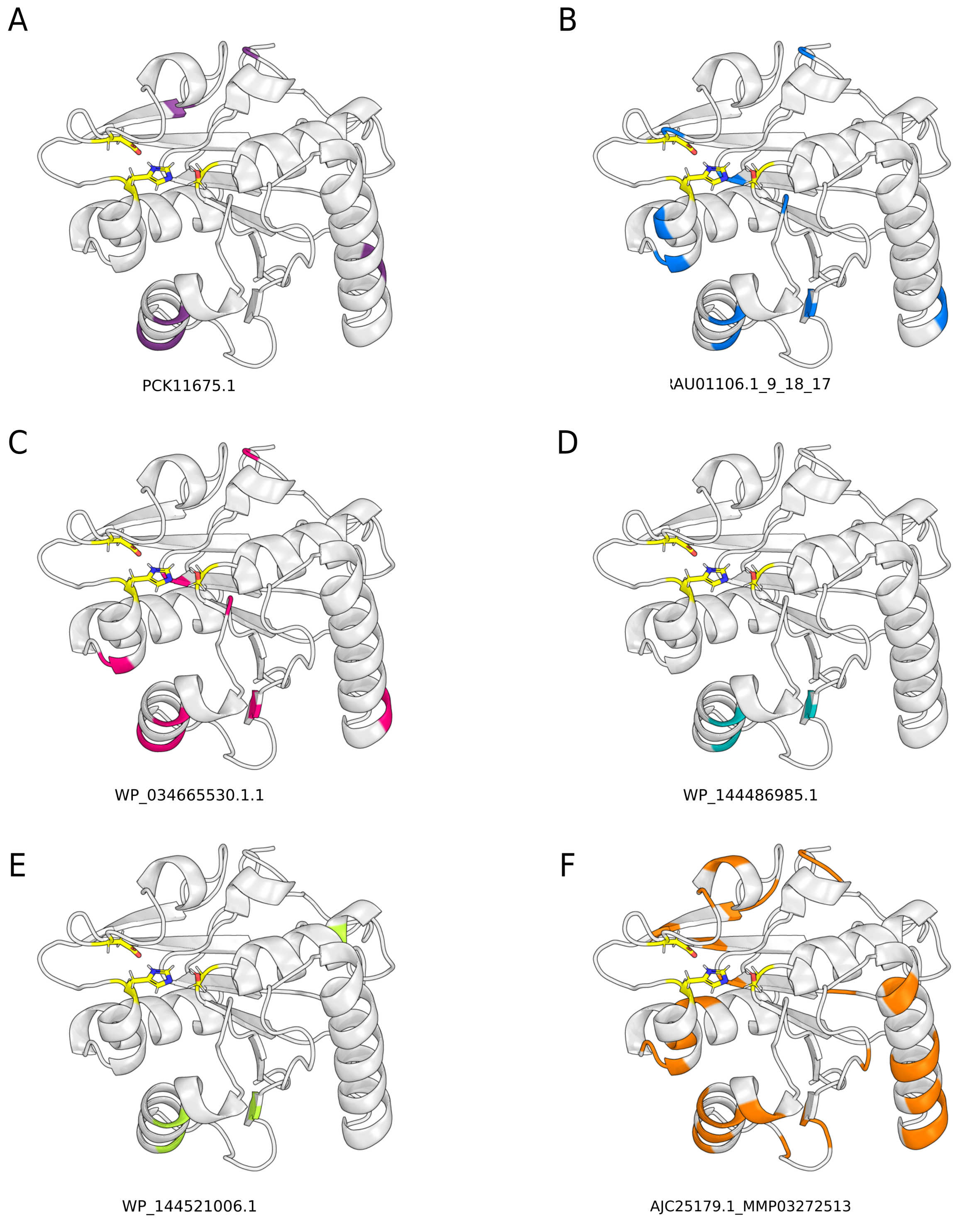

Supplementary Figure 6: Structural models of Lip$_{MRD9}$ homologs highlighting sequence diversity. Panels A–F show six homologous proteins of Lip$_{MRD9}$ with distinct sequence variations mapped onto their 3D structures. Catalytic residues (yellow, sticks) are shown for all structures.

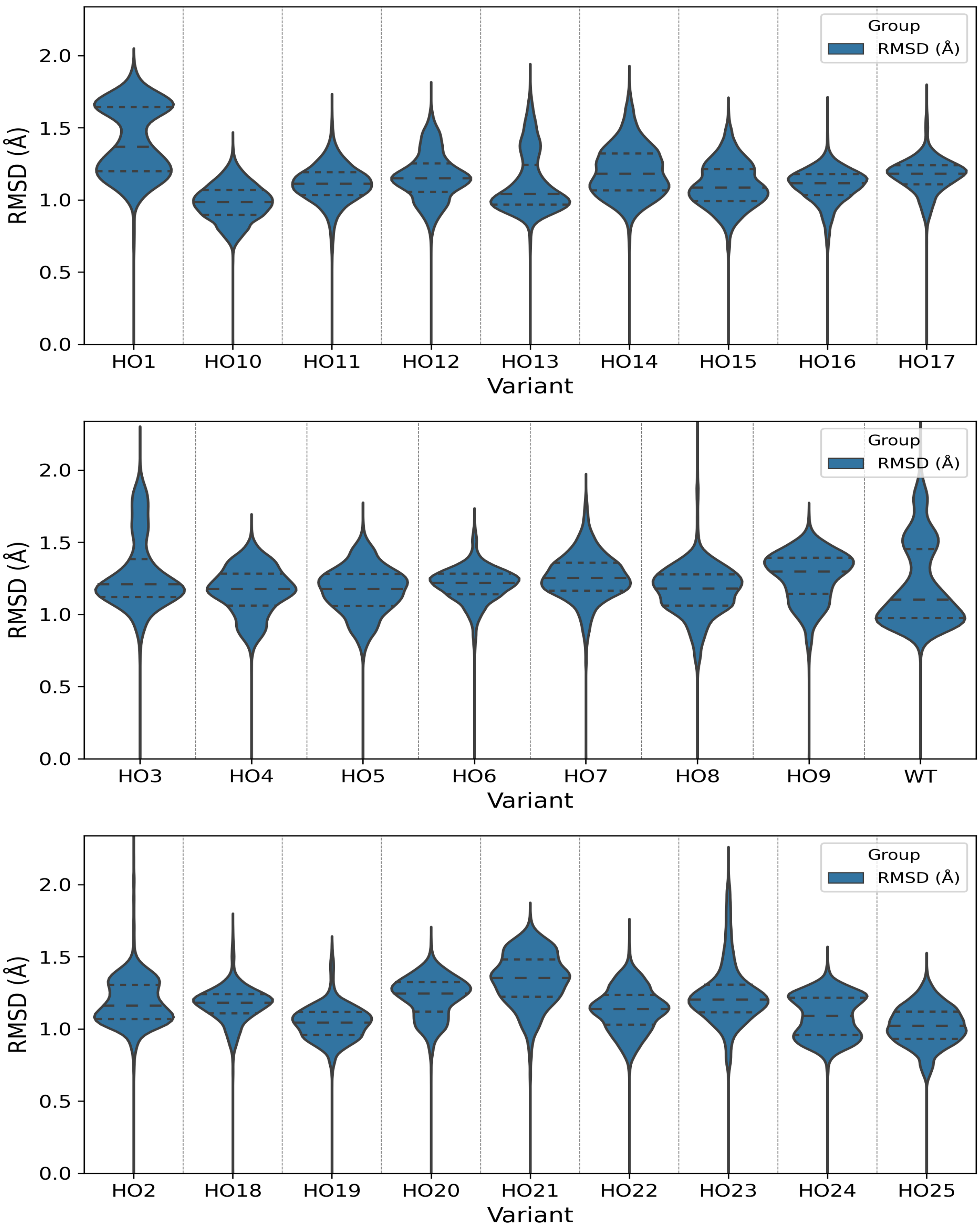

Supplementary Figure 7: Distribution of the root mean square deviation (RMSD) along the 3 replicas of 200 ns of MD simulations for the wildtype and the different homologs variants with TOL substrate. Boxplots show medians (line), interquartile ranges (boxes), and full ranges (whiskers).

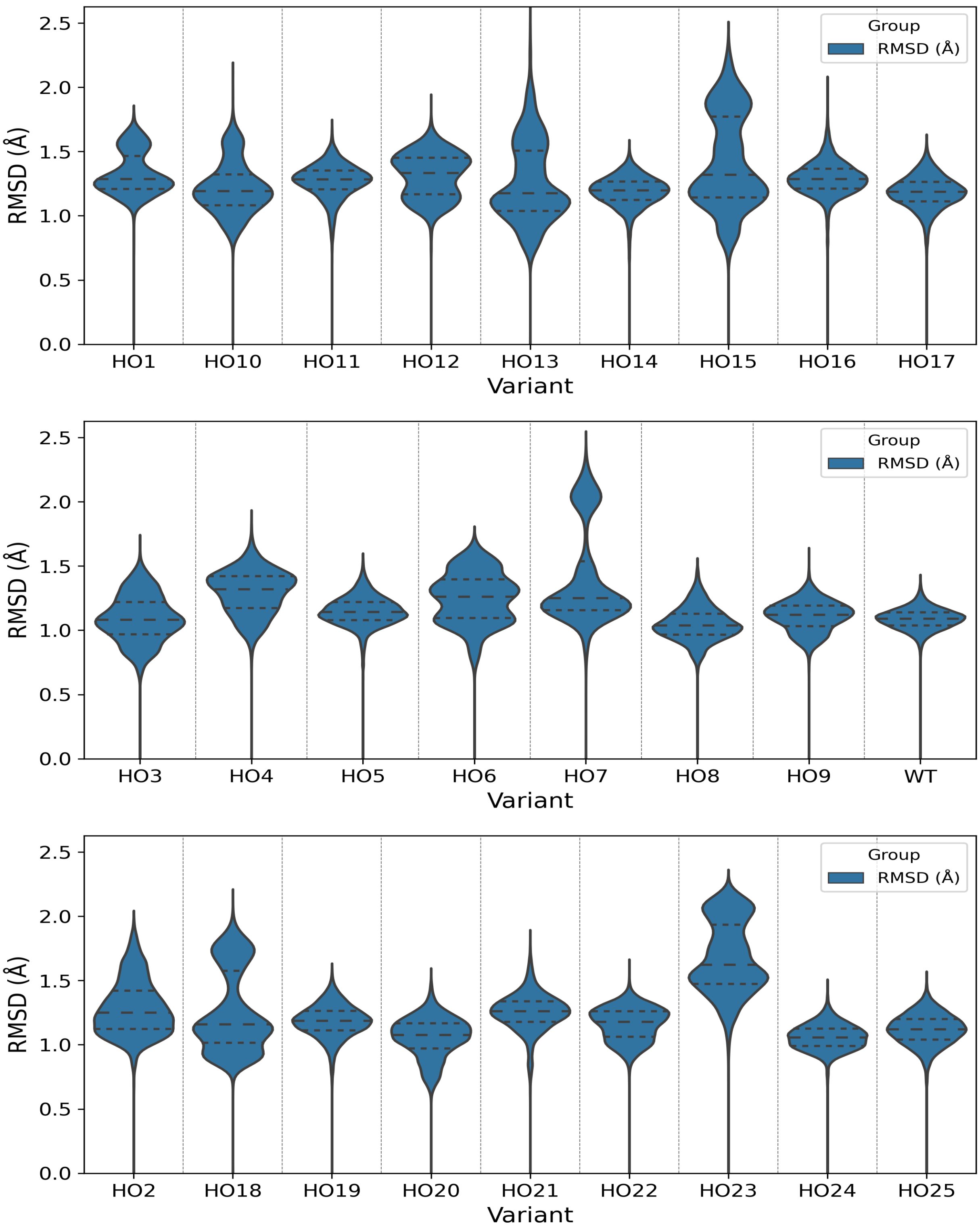

Supplementary Figure 8: Distribution of the root mean square deviation (RMSD) along the 3 replicas of 200 ns of MD simulations for the wildtype and the different homologs with PT4 substrate. Boxplots show medians (line), interquartile ranges (boxes), and full ranges (whiskers).

```
                                                10        20        30        40        50        60        70        80
                                       ....|....| ....|....| ....|....| ....|....| ....|....| ....|....| ....|....| ....|....|
WP_060597018.1_MMP03893210             MKVIRFKKKS LQILVALALV IGSMAFIQPK -EIKAAEHNP VVMVHGMGGA SYNFASIKSY LVSQGWDRNQ LFAIDFIDKT
WP_060597018.1                         MKVIRFKKKS LQILVALALV IGSMAFIQPK -EIKAAEHNP VVMVHGMGGA SYNFASIKSY LVSQGWDRNQ LFAIDFIDKT
tr|A0A7H4U7R2|A0A7H4U7R2_BACPU         MKVIRFKKKS LQILVALALV IGSMAFIQPK -EIKAAEHNP VVMVHGMGGA SYNFASIKSY LVSQGWDRNQ LFAIDFIDKT
tr|A0A2T0CZ91|A0A2T0CZ91_9BACI         MKVIRFKKKS LQILVALALV IGSMAFIQPK -EIKAAEHNP VVMVHGMGGA SYNFASIKSY LVSQGWDRNQ LFAIDFIDKT
WP_066030544.1 (H21)                   MKVIRFKKKS LQILVALALV IGSMAFIQPK -EIEAAEHNP VVMVHGMGGA SYNFASIKSY LVSQGWDRNQ LFAIDFIDKT
WP_034665530.1 (H16)                   MKVIRFKKRS LQILVVLALV MGSMAFIQPK -EIRAAEHNP VVMVHGMGGA SYNFASIKSY LVSQGWDRNQ LFAIDFIDKT
tr|A1E152|A1E152_BACPU                 MKVIRFKKRS LQILVVLALV MGSMAFIQPK -EIRAAEHNP VVMVHGMGGA SYNFASIKSY LVSQGWDRNQ LFAIDFIDKT
WP_095286143.1 (H22)                   MKVIRFKKRS LQILVVLALV MGSMAFIQPK -EIRAAEHNP VVMVHGMGGA SYNFASIKSY LVSQGWDRNQ LFAIDFIDKT
tr|A0A267X6U6|A0A267X6U6_9BACI         MKVIRFKKRS LQILVVLALV MGSMAFIQPK -EIRAAEHNP VVMVHGMGGA SYNFASIKSY LVSQGWDRNQ LFAIDFIDKT
WP_106065766.1                         MKVIRFKKKS LQILVALALV IGSMAFIQPK -EIKAAEHNP VVMVHGMGGA SYNFASIKSY LVTQGWDRNQ LFAIDFIDKT
tr|A0A2T0CG63|A0A2T0CG63_BACPU         MKVIRFKKKS LQILVALALV IGSMAFIQPK -EIKAAEHNP VVMVHGMGGA SYNFASIKSY LVTQGWDRNQ LFAIDFIDKT
RAP25570.1                             --MIRFKKKS LQILVALALV IGSMAFIQPK -EIKAAEHNP VVMVHGMGGA SYNFASIKSY LVTQGWDRNQ LFAIDFIDKT
KIL10233.1 (H4)                        --MICFKKKS LQILVALALV IGSMAFIQPK -EIKAAEHNP VVMVHGMGGA SYNFASIKSY LVTQGWDRNQ LFAIDFIDKT
WP_044142215.1                         MKVICFKKKS LQILVALALV IGSMAFIQPK -EIKAAEHNP VVMVHGMGGA SYNFASIKSY LVTQGWDRNQ LFAIDFIDKT
WP_065098612.1 (H20)                   MKVICFKKKS LQILVALALV IGSMAFIQPK -EIKAAEHNP VVMVHGMGGA SYNFASIKSY LVTQGWDRNQ LFAIDFIDKT
tr|W8FKE7|W8FKE7_BACPU                 MKVICFKKKS LQILVALALV IGSMAFIQPK -EIKAAEHNP VVMVHGMGGA SYNFASIKSY LVTQGWDRNQ LFAIDFIDKT
WP_106066877.1 (H23)                   MKVICFKKKS LQILVALALV IGSMAFIQSK -EIKAAEHNP VVMVHGMGGA SYNFASIKSY LVTQGWDRNQ LFAIDFIDKT
RAU01106.1 (H9)                        --MICFKKKS LQILVALALV IGSMAFIQPK -EIKAAEHNP VVMVHGMGGA SYNFASIKSY LVTQGWDRNQ LFAIDFIDKT
WP_148666205.1                         MKVILFKKRS LQILVAFALV IGSMAFIQPK -EIKAAEHNP VVMVHGMGGA SYNFASIKSY LVSQGWDRNQ LFAIDFIDKT
PRR93548.1 (H8)                        --MILFKKRS LQILVAFALV IGSMAFIQPK -EIKAAEHNP VVMVHGMGGA SYNFASIKSY LVSQGWDRNQ LFAIDFIDKT
WP_144521006.1 (H25)                   MKVIRFKKKS LQILVALALV IGSMAFIQPK -EIKAAEHNP VVMVHGIGGA SYNFASIKSY LVTQGWDRNQ LFAIDFIDKT
WP_144486985.1 (H24)                   MKVIRLKKKS LQILVALALV IGSMAFIQPK -EIKAAEHNP VVMVHGIGGA SYNFASIKSY LVTQGWDRNQ LFAIDFIDKT
WP_050943555.1_MMP03329560 (H1         MKVICFKKKS LQILVALALV IGSMAFIQPK -EIKAAEHNP VVMVHGIGGA SYNFASIKSY LVTQGWDRNK LFAIDFIDKT
WP_050943555.1_MMP08568348 (H1         MKVICFKKKS LQILVALALV IGSMAFIQPK -EIKAAEHNP VVMVHGIGGA SYNFASIKSY LVTQGWDRNK LFAIDFIDKT
WP_050943555.1 (H17)                   MKVICFKKKS LQILVALALV IGSMAFIQPK -EIKAAEHNP VVMVHGIGGA SYNFASIKSY LVTQGWDRNK LFAIDFIDKT
tr|A0A4V5TAE0|A0A4V5TAE0_BACPU         MKVICFKKKS LQILVALALV IGSMAFIQPK -EIKAAEHNP VVMVHGIGGA SYNFASIKSY LVTQGWDRNK LFAIDFIDKT
tr|A0A1E1FNX8|A0A1E1FNX8_BACPU         MKVILFKKRS LQILVALALV IGSMAFIQPK -EVKAAEHNP VVMVHGIGGA SYNFASIKSY LVGQGWDRNQ LYAIDFIDKT
tr|A0A2Y9UMQ0|A0A2Y9UMQ0_9BACI         MKVILFKKRS LQILVALALV IGSMAFIQPK -EVKAAEHNP VVMVHGIGGA SYNFASIKSY LVGQGWDRNQ LYAIDFIDKT
WP_060697639.1_MMP03892176             MKVILFKKRS LQILVALALV IGSMAFIQPK -EVKAAEHNP VVMVHGIGGA SYNFASIKSY LVGQGWDRNQ LYAIDFIDKT
WP_039180664.1_MMP03976074             MKVIFVKKRS LQILVALALV IGSMAFIQPK -EVKAAEHNP VVMVHGIGGA SYNFYSIKSY LVGQGWDRNQ LYAIDFIDKT
PCK11675.1_MMP07270366 (H6)            MKVIFVKKRS LQILVALALV IGSMAFIQPK -EVKAAEHNP VVMVHGIGGA SYNFYSIKSY LVGQGWDRNQ LYAIDFIDKT
tr|Q6RSN0|Q6RSN0_BACPU                 MKVILFKKRS LQILVALALV IGSMAFIQPK -EVKAAEHNP VVMVHGIGGA SYNFASIKSY LVGQGWDRNQ LYAIDFIDKT
WP_034624255.1_MMP02471694             MKVMFVKKRS LQILIALALV IGSMAFIQPK -EVKAAEHNP VVMVHGIGGA SYNFFSIKSY LATQGWDRNQ LYAIDFIDKT
WP_034624255.1_MMP06016472 (Li         MKVMFVKKRS LQILIALALV IGSMAFIQPK -EVKAAEHNP VVMVHGIGGA SYNFFSIKSY LATQGWDRNQ LYAIDFIDKT
OUZ07106.1_MMP05730644 (H5)            MKVSLLMKRS LQILVAFALV IGSMAFIQPK -EVKAAEHNP VVMVHGIGGA SYNFASIKSY LVGQGWDRNQ LFAIDFIDKT
WP_169056738.1                         MKVIRFKKKS LQILVALALV LGSMAFIQPK -EIKAAEHNP VVMVHGMGGA SYNFASIKSY LVTQGWDRNQ LFAIDFIDKT
WP_058015050.1                         MKVIRFKKKS LQILVALALV LGSMAFIQPK -EIKAAEHNP VVMVHGMGGA SYNFASIKSY LVTQGWDRNQ LFAIDFIDKT
```

Supplementary Figure 9: (page 1 of 6) Multiple sequence alignment of Lip$_{MRD9}$ with 69 homologous sequences. The alignment was performed using ClustalW, and the graphical representation was generated with BioEdit 7. Conserved residues are shown in white, whereas non-conserved residues across the sequences are displayed with a black background.

```
WP_106042678.1                       MKVIRFKKKS LQILVALALV MGSMAFIQPK -EIKAAEHNP VVMVHGMGGA SYNFASIKSY LVSQGWDRNQ LFAIDFIDKT
tr|H6U4T6|H6U4T6_9BACI               MKVIRFKKKS LQILVALALV LGSMAFIQPK -EVKAAAHNP VVMVHGMGGA SYNFASIKRY LVSQGWDQNQ LFAIDFIDKT
tr|W8RKH7|W8RKH7_BACPU               MKVIRFKKKS LQILVALALV LGSMAFIQPK -EVKAAAHNP VVMVHGMGGA SYNFASIKRY LVSQGWDQNQ LFAIDFIDKT
WP_025207804.1_MMP05216491           MKVIRFKKKS LQILVALALV LGSMAFIQPK -EVKAAAHNP VVMVHGMGGA SYNFASIKRY LVSQGWDQNQ LFAIDFIDKT
TFW47892.1_MMP10790525 (H11)         MKVIRFKKKS LQILVALALV LGSMAFIQPK -EVKAAAHNP VVMVHGMGGA SYNFASIKRY LVSQGWDQNQ LFAIDFIDKT
tr|A0A0N9DV87|A0A0N9DV87_9BACI       MKVIRFKKKS LQILVALALV LGSMAFIQPK -EVKAAAHNP VVMVHGMGGA SYNFASIKRY LVSQGWDQNQ LFAIDFIDKT
tr|A0A5K1KU97|A0A5K1KU97_9BACI       MKVIRFKKKS LQILVALALV LGSMAFIQPK -EVKAAAHNP VVMVHGMGGA SYNFASIKRY LVTQGWDQNQ LFAIDFIDKT
SPR92696.1_MMP4665500 (H10)          --MIRFKKKS LQILVALALV LGSMAFIQPK -EVKAAAHNP VVMVHGMGGA SYNFASIKRY LVTQGWDQNQ LFAIDFIDKT
AJW76889.1 (H2)                      MKVICFKKKS LQILVALALV IGSMAFIQPK -EVKAAAHNP VVMVHGMGGA SYNFASIKRY LVSQGWDQNQ LFAIDFIDKT
tr|A0A0D5CCJ2|A0A0D5CCJ2_9BACI       MKVICFKKKS LQILVALALV IGSMAFIQPK -EVKAAAHNP VVMVHGMGGA SYNFASIKRY LVSQGWDQNQ LFAIDFIDKT
WP_007501788.1_MMP02471373           MKVIRFKKKS LQILVALALV LGSMAFIQPK -EAKAAAHNP VVMVHGMGGA SYNFASIKRY LVSQGWDQNQ LFAIDFIDKT
tr|M5RBD1|M5RBD1_9BACI               MKVIRFKKKS LQILVALALV LGSMAFIQPK -EAKAAAHNP VVMVHGMGGA SYNFASIKRY LVSQGWDQNQ LFAIDFIDKT
WP_041092577.1_MMP04263913           MKVIRFKKKS LQILVALALV LGSIAFIQPK -EAKAAEHNP VVMVHGMGGA SYNFASIKRY LVSQGWDQNQ LFAIDFIDKT
tr|A0A6G5TBN3|A0A6G5TBN3_9BACI       MKVIRFKKKS LQILVALALV LGSIAFIQPK -EAKAAEHNP VVMVHGMGGA SYNFASIKRY LVSQGWDQNQ LFAIDFIDKT
tr|Q8VU78|Q8VU78_9BACI               MKVIRFKKKS LQILVALALV LGSIAFIQPK -EAKAAEHNP VVMVHGMGGA SYNFASIKRY LVSQGWDQNQ LFAIDFIDKT
OPW99383.1_MMP05726035               MKVIRFKKKS LQILVALALV LGSIAFIQPK -EAKAAEHNP VVMVHGMGGA SYNFASIKRY LVSQGWDQNQ LFAIDFIDKT
tr|K4JG13|K4JG13_BACPU               MKVMLFKKKS LQILVALALV IGSMAFIQPK -EAKAAEHNP VVMVHGMGGA SYNFASIKRY LVSQGWDQNQ LFVIDFIDKT
WP_056705505.1_MMP11621488           --MIVLKKRS LQILIALALV LGSMVFIQPK -EVKAAAHNP VVMVHGMGGA SYNFASIKRY LVTQGWDQNQ LFAIDFIDKT
WP_008356351.1_MMP02471164 (H1       --MIVLKKRS LQILIALALV IGSMAFIQPK -EVKAAEHNP VVMVHGMGGA SYNFASIKRY LVSQGWDQNQ LFAIDFIDKT
tr|U3MG45|U3MG45_BACPU               MKVIRFKKRS LQILIALALV VGSMAFVQPK -EAKAAEHNP VVMVHGMGGA SYNFASIKRY LVSQGWDQNQ LFAIDFIDKT
WP_034322408.1_MMP02870955           MKVKLFIKRS LQILVAFALV VGSMVFIQPK -EVKAAEHNP VVMVHGMGGA SYNFAAIKRY LVSQGWDRNQ LYAIDFIDKT
PCK20493.1_MMP07270068               ---------- --MFVALILV AGSLVFIQPK -KVKAAEHNP VVMVHGMGGA SYNFAYIKRY LVSQGWNQNQ LFAIDFIDKT
WP_007409033.1_MMP04296795 (H1       --MKQIKSKI LAILTVCMLS VISVFAFQPT VSKASSEHNP VVMVHGIGGA SFNFAGIKTY LASQGWSRNE MYAIDFLDKT
PHQ06896.1_MMP07514152 (H7)          --MKQIKSKI LAILTVCMLS VISVFAFQPT VSKASSEHNP VVMVHGIGGA SFNFAGIKTY LASQGWSRNE MYAIDFLDKT
AJC25179.1_MMP03272513 (H1)          --MKQIKSKI LAILTVCMLS VISVFAFQPT VSKASSEHNP VVMVHGIGGA SFNFAGIKTY LASQGWSRNE MYAIDFLDKT
WP_007409033.1_MMP09428134 (H1       --MKQIKSKI LAILTVCMLS VISVFAFQPT VSKASSEHNP VVMVHGIGGA SFNFAGIKTY LASQGWSRNE MYAIDFLDKT
WP_017419343.1_MMP05517979           --MKHIKSKI LVILTVCMLS VISVFAFQPT VSKASSGHNP VVMVHGIGGA SFNFAGIKTY LASQGWSRKE MYAIDFLDKT
WP_003327989.1_MMP02470933 (H1       --MNAVKSKL LAVLMICMLS VVSVFTIQPN VSKAASEHNP VVMVHGIGGA SFNFAGIKSY LVSQGWSSKD LYAIDFLDKT
sp|P37957|ESTA_BACSU                 --MKFVKRRI IALVTILMLS VTSLFALQP- -SAKAAEHNP VVMVHGIGGA SFNFAGIKSY LVSQGWSRDK LYAVDFWDKT
QBJ80898.1_MMP09273200               --MKFVKRRI ITLVTILMLS VTSLFALQP- -SAKAAEHNP VVMVHGIGGA SFNFAGIKSY LVSQGWSRDK LYAVDFWDKT
AXC51599.1_MMP09500751 (H3)          --MKFVKRRI VALVTILMLS VTSLFALQP- -SAKAAEHNP VVMVHGIGGA SFNFAGIKSY LVSQGWSRDK LYAVDFWDKT
WP_086343408.1_MMP11519450           --MKFVKRRI IALVTILMLS VTSLFALQP- -SAKAAEHNP VVMVHGIGGA SFNFAGIKSY LVSQGWSRDK LYAVDFWDKT
WP_052471036.1_MMP13053465           ---------- -------MLS VTSLFALQP- -SAKAAEHNP VVMVHGIGGA SFNFAGIKSY LVSQGWSRDK LYAVDFWDKT

                                             90        100        110        120        130        140        150        160
                                     ....|....| ....|....| ....|....| ....|....| ....|....| ....|....| ....|....| ....|....|
WP_060597018.1_MMP03893210           GNNRNNGPRL SRFVKDVLDK TGAKKVDIVA HSMGGANTLY YIKNLDGGDK IENVVTLGGA NGLVSLRALP GTDPNQKILY
WP_060597018.1                       GNNRNNGPRL SRFVKDVLDK TGAKKVDIVA HSMGGANTLY YIKNLDGGDK IENVVTLGGA NGLVSLRALP GTDPNQKILY
tr|A0A7H4U7R2|A0A7H4U7R2_BACPU       GNNRNNGPRL SRFVKDVLDK TGAKKVDIVA HSMGGANTLY YIKNLDGGDK IENVVTLGGA NGLVSLRALP GTDPNQKILY
```

(page 2 of 6) Continued from Supplementary Figure 9

**tr|A0A2T0CZ91|A0A2T0CZ91_9BACI**	GNNRNNGPRL SRFVKDVLDK TGAKKVDIVA HSMGGANTLY YIKNLDGGDK IENVVTLGGA NGLVSLRALP GTDPNQKILY
**WP_066030544.1 (H21)**	GNNRNNGPRL SRFVKDVLDK TGAKKVDIVA HSMGGANTLY YIKNLDGGDK IENVVTLGGA NGLVSLRALP GTDPNQKILY
**WP_034665530.1 (H16)**	GNNRNNGPRL SRFVKDVLAK TGAKKVDIVA HSMGGANTLY YIKNLDGGDK IENVVTLGGA NGLVSLRALP GTDPNQKILY
**tr|A1E152|A1E152_BACPU**	GNNRNNGPRL SRFVKDVLAK TGAKKVDIVA HSMGGANTLY YIKNLDGGDK IENVVTLGGA NGLVSLRALP GTDPNQKILY
**WP_095286143.1 (H22)**	GNNRNNGPRL SRFVKDVLAK TGAKKVDIVA HSMGGANTLY YIKNLDGGDK IANVVTLGGA NGLVSLRALP GTDPNQKILY
**tr|A0A267X6U6|A0A267X6U6_9BACI**	GNNRNNGPRL SRFVKDVLAK TGAKKVDIVA HSMGGANTLY YIKNLDGGDK IANVVTLGGA NGLVSLRALP GTDPNQKILY
**WP_106065766.1**	GNNRNNGPRL SRFVKDVLGK TGAKKVDIVA HSMGGANTLY YIKNLDGGDK IENVVTLGGA NGLVSLRALP GTDPNQKILY
**tr|A0A2T0CG63|A0A2T0CG63_BACPU**	GNNRNNGPRL SRFVKDVLGK TGAKKVDIVA HSMGGANTLY YIKNLDGGDK IENVVTLGGA NGLVSLRALP GTDPNQKILY
**RAP25570.1**	GNNRNNGPRL SRFVKDVLGK TGAKKVDIVA HSMGGANTLY YIKNLDGGDK IENVVTLGGA NGLVSLRALP GTDPNQKILY
**KIL10233.1 (H4)**	GNNRNNGPRL SRFVKDVLGK TGAKKVDIVA HSMGGANTLY YIKNLDGGDK IENVVTLGGA NGLVSLRALP GTDPNQKILY
**WP_044142215.1**	GNNRNNGPRL SRFVKDVLGK TGAKKVDIVA HSMGGANTLY YIKNLDGGDK IENVVTLGGA NGLVSLRALP GTDPNQKILY
**WP_065098612.1 (H20)**	GNNRNNGPRL SRFVKDVLGK TGAKKVDIVA HSMGGANTLY YIKNLDGGDK IENVVTLGGA NGLVSLRALP GTDPNQKILY
**tr|W8FKE7|W8FKE7_BACPU**	GNNRNNGPRL SRFVKDVLGK TGAKKVDIVA HSMGGANTLY YIKNLDGGDK IENVVTLGGA NGLVSLRALP GTDPNQKILY
**WP_106066877.1 (H23)**	GNNRNNGPRL SRFVKDVLGK TGAKKVDIVA HSMGGANTLY YIKNLDGGDK IENVVTLGGA NGLVSLRALP GTDPNQKILY
**RAU01106.1 (H9)**	GNNRNNGPRL SRFVKDVLGK TGAKKVDIVA HSMGGANTLY YIKNLDGGDK IENVVTLGGA NGLVSLRALP GTDPNQKILY
**WP_148666205.1**	GNNLNNGPRL SRFVKDVLGK TGAKKVDIVA HSMGGANTLY YIKNLDGGDK IENVVTLGGA NGLVSLRALP GTDPNQKILY
**PRR93548.1 (H8)**	GNNLNNGPRL SRFVKDVLGK TGAKKVDIVA HSMGGANTLY YIKNLDGGDK IENVVTLGGA NGLVSLRALP GTDPNQKILY
**WP_144521006.1 (H25)**	GNNRNNGPRL SRFVKDVLDK TGAKKVDIVA HSMGGANTLY YVKNLDGGDK IENVVTIGGA NGLVSSRALP GTDPNQKILY
**WP_144486985.1 (H24)**	GNNRNNGPRL SRFVKDVLDK TGAKKVDIVA HSMGGANTLY YIKNLDGGDK IENVVTIGGA NGLVSSRALP GTDPNQKILY
**WP_050943555.1_MMP03329560 (H1**	GNNRNNGPRL SRFVKDVLDK TGAKKVDIVG HSMGGANTLY YIKNLDGGDK IENVVTIGGA NGLVSSRALP GTDPNQKILY
**WP_050943555.1_MMP08568348 (H1**	GNNRNNGPRL SRFVKDVLDK TGAKKVDIVG HSMGGANTLY YIKNLDGGDK IENVVTIGGA NGLVSSRALP GTDPNQKILY
**WP_050943555.1 (H17)**	GNNRNNGPRL SRFVKDVLDK TGAKKVDIVG HSMGGANTLY YIKNLDGGDK IENVVTIGGA NGLVSSRALP GTDPNQKILY
**tr|A0A4V5TAE0|A0A4V5TAE0_BACPU**	GNNRNNGPRL SRFVKDVLDK TGAKKVDIVG HSMGGANTLY YIKNLDGGDK IENVVTIGGA NGLVSSRALP GTDPNQKILY
**tr|A0A1E1FNX8|A0A1E1FNX8_BACPU**	GNNRNNGPRL SKFVQDVLDK TGAKKVDIVA HSMGGANTLY YIKNLDGGDK IENVVTIGGA NGLVSSRALP GTDPNQKILY
**tr|A0A2Y9UMQ0|A0A2Y9UMQ0_9BACI**	GNNRNNGPRL SKFVQDVLDK TGAKKVDIVA HSMGGANTLY YIKNLDGGDK IENVVTIGGA NGLVSSRALP GTDPNQKILY
**WP_060697639.1_MMP03892176**	GNNRNNGPRL SKFVQDVLNK TGAKKVDIVA HSMGGANTLY YIKNLDGGDK IENVVTIGGA NGLVSSRALP GTDPNQKILY
**WP_039180664.1_MMP03976074**	GNNRNNGPRL SRFVQDVLDK TGAKKVDIVA HSMGGANTLY YIKNLDGGDK IENVVTIGGA NGLVSNRALP GTDPNQKILY
**PCK11675.1_MMP07270366 (H6)**	GNNRNNGPRL SRFVQDVLDK TGAKKVDIVA HSMGGANTLY YIKNLDGGDK IENVVTIGGA NGLVSNRALP GTDPNQKILY
**tr|Q6RSN0|Q6RSN0_BACPU**	GNNRNNGPRL SRFVKDVLDK TGAKKVDIVA HSMGGANTLY YIKNLDGGDK IENVVTIGGA NGLVSSRALP GTDPNQKILY
**WP_034624255.1_MMP02471694**	GNNRNNGPRL SRFVKDVLDK TGAKKVDIVA HSMGGANTLY YIKNLDGGDK IENVVTIGGA NGLVSSRALP GTDPNQKILY
**WP_034624255.1_MMP06016472 (Li**	GNNRNNGPRL SRFVKDVLDK TGAKKVDIVA HSMGGANTLY YIKNLDGGDK IENVVTIGGA NGLVSSRALP GTDPNQKILY
**OUZ07106.1_MMP05730644 (H5)**	GNNRNNGPRL SRFVKDVLDK TGAKKVDIVA HSMGGANTLY YIKNLDGGDK IENVVTIGGA NGLVSSRALP GTDPNQKILY
**WP_169056738.1**	GNNRNNGPRL SRFVKDVLDK TGAKKVDIVA HSMGGANTLY YIKNLDGGDK IENVVTLGGA NGLVSLRALP GTDPNQKILY
**WP_058015050.1**	GNNRNNGPRL SRFVKDVLDK TGAKKVDIVA HSMGGANTLY YIKNLDGGDK IENVVTLGGA NGLVSLRALP GTDPNQKILY
**WP_106042678.1**	GNNRNNGPRL SRFVKDVLAK TGAKKVDIVA HSMGGANTLY YIKNLDGGDK IENVVTLGGA NGLVSLRALP GTDPNQKILY
**tr|H6U4T6|H6U4T6_9BACI**	GNNLNNGPRL SRFVKDVLAK TGAKKVDIVA HSMGGANTLY YIKNLDGGDK IENVVTLGGA NGLVSLRALP GTDPNQKILY
**tr|W8RKH7|W8RKH7_BACPU**	GNNLNNGPRL SRFVKDVLAK TGAKKVDIVA HSMGGANTLY YIKNLDGGDK IENVVTLGGA NGLVSLRALP GTDPNQKILY
**WP_025207804.1_MMP05216491**	GNNLNNGPRL SRFVKDVLAK TGAKKVDIVA HSMGGANTLY YIKNLDGGDK IENVVTLGGA NGLVSLRALP GTDPNQKILY
**TFW47892.1_MMP10790525 (H11)**	GNNLNNGPRL SRFVKDVLAK TGAKKVDIVA HSMGGANTLY YIKNLDGGDK IENVVTLGGA NGLVSLRALP GTDPNQKILY

(page 3 of 6) Continued from Supplementary Figure 9

```
tr|A0A0N9DV87|A0A0N9DV87_9BACI GNNLNNGPRL SRFVKDVLAK TGAKKVDIVA HSMGGANTLY YIKNLDGGDK IENVVTLGGA NGLVSLRALP GTDPNQKILY
tr|A0A5K1KU97|A0A5K1KU97_9BACI GNNLNNGPRL SRFVKDVLAK TGAKKVDIVA HSMGGANTLY YIKNLDGGDK IENVVTLGGA NGLVSLRALP GTDPNQKILY
SPR92696.1_MMP4665500 (H10)    GNNLNNGPRL SRFVKDVLAK TGAKKVDIVA HSMGGANTLY YIKNLDGGDK IENVVTLGGA NGLVSLRALP GTDPNQKILY
AJW76889.1 (H2)                GNNLNNGPRL SRFVKDVLAK TGAKKVDIVA HSMGGANTLY YIKNLDGGDK IENVVTLGGA NGLVSLRALP GTDPNQKILY
tr|A0A0D5CCJ2|A0A0D5CCJ2_9BACI GNNLNNGPRL SRFVKDVLAK TGAKKVDIVA HSMGGANTLY YIKNLDGGDK IENVVTLGGA NGLVSLRALP GTDPNQKILY
WP_007501788.1_MMP02471373     GNNLNNGPRL SRFVKDVLAK TGAKKVDIVA HSMGGANTLY YIKNLDGGDK IENVVTLGGA NGLVSLRALP GTDPNQKILY
tr|M5RBD1|M5RBD1_9BACI         GNNLNNGPRL SRFVKDVLAK TGAKKVDIVA HSMGGANTLY YIKNLDGGDK IENVVTLGGA NGLVSLRALP GTDPNQKILY
WP_041092577.1_MMP04263913     GNNLNNGPRL SRFVKDVLAK TGAKKVDIVA HSMGGANTLY YIKNLDGGDK IENVVTLGGA NGLVSLRALP GTDPNQKILY
tr|A0A6G5TBN3|A0A6G5TBN3_9BACI GNNLNNGPRL SRFVKDVLAK TGAKKVDIVA HSMGGANTLY YIKNLDGGDK IENVVTLGGA NGLVSLRALP GTDPNQKILY
tr|Q8VU78|Q8VU78_9BACI         GNNLNNGPRL SRFVKDVLAK TGAKKVDIVA HSMGGANTLY YIKNLDGGDK IENVVTLGGA NGLVSLRALP GTDPNQKILY
OPW99383.1_MMP05726035         GNNLNNGPRL SRFVKDVLDK TGAKKVDIVA HSMGGANTLY YIKNLDGGDK IENVVTLGGA NGLVSLRALP GTDPNQKILY
tr|K4JG13|K4JG13_BACPU         GNNLNNGPRL SRFVKDVLAK TGAKKVDIVA HSMGGANTLY YIKNLDGGDK IENVVTLGGA NGLVSLRALP GTDPNQKILY
WP_056705505.1_MMP11621488     GNNLNNGPRL SRFVKDVLAK TGAKKVDIVA HSMGGANTLY YIKNLDGGDK IENVVTLGGA NGLVSLRALP GTDPNQKILY
WP_008356351.1_MMP02471164 (H1 GNNLNNGPRL SRYVKDVLAK TGAKKVDIVA HSMGGANTLY YIKNLDGGDK IENVVTLGGA NGLVSLRALP GTDPNQKILY
tr|U3MG45|U3MG45_BACPU         GNNLNNGPRL SRFVKDVLAK TGAKKVDIVA HSMGGANTLY YIKNLDGGDK IENVVTLGGA NGLVSLRALP GTDPNQKILY
WP_034322408.1_MMP02870955     GNNLNNGPRL SRYVKDVLAK TGAKKVDIVA HSMGGANTLY FIKNLDGGDK IENVVTLGGA NGLVSLRALP GTDPNQKILY
PCK20493.1_MMP07270068         GNNLNNGPRL SRYVKDVLVK TGAKKVDIVA HSMGGANTLY YIKNLDGGNK IANVVTLGGA NGLVSFKALP GTDPNQKILY
WP_007409033.1_MMP04296795 (H1 GNNRHNAPRL SNYVKKVLSE TGAKKVDIVA HSMGGANTLY YIKNLDGGDK IANVVTLGGA NGLVTNRALP GTDPNQKILY
PHQ06896.1_MMP07514152 (H7)    GNNRHNAPRL SNYVKKVLSE TGAKKVDIVA HSMGGANTLY YIKNLDGGDK IANVVTLGGA NGLVTNRALP GTDPNQKILY
AJC25179.1_MMP03272513 (H1)    GNNRHNAPRL SNYVKKVLSE TGAKKVDIVA HSMGGANTLY YIKNLDGGDK IANVVTLGGA NGLVTNRALP GTDPNQKILY
WP_007409033.1_MMP09428134 (H1 GNNRHNAPRL SNYVKKVLSE TGAKKVDIVA HSMGGANTLY YIKNLDGGDK IANVVTLGGA NGLVTNRALP GTDPNQKILY
WP_017419343.1_MMP05517979     GNNRHNAPRL SNYVKKVLSE TGASKVDIVA HSMGGANTLY YIKNLDGGDK IANVVTLGGA NGLVTNRALP GTDPNQKILY
WP_003327989.1_MMP02470933 (H1 GNNRNNAPRL SSYVKKVLSE TGASKVDIVA HSMGGANTLY YIKNLDGGDK IANVVTLGGA NGLVTNRALP GTDPNQKILY
sp|P37957|ESTA_BACSU           GTNYNNGPVL SRFVQKVLDE TGAKKVDIVA HSMGGANTLY YIKNLDGGNK VANVVTLGGA NRLTTGKALP GTDPNQKILY
QBJ80898.1_MMP09273200         GTNYNNGPVL SRFVQKVLDE TGAKKVDIVA HSMGGANTLY YIKNLDGGNK VANVVTLGGA NRLTTGKALP GTDPNQKILY
AXC51599.1_MMP09500751 (H3)    GTNYNNGPVL SRFVQKVLDE TGAKKVDIVA HSMGGANTLY YIKNLDGGNK VANVVTLGGA NRLTTGKALP GTDPNQKILY
WP_086343408.1_MMP11519450     GTNYNNGPVL SRFVQKVLDE TGAKKVDIVA HSMGGANTLY YIKNLDGGNK IANVVTLGGA NRLTTGKALP GTDPNQKILY
WP_052471036.1_MMP13053465     GTNYNNGPVL SRFVQKVLDE TGAKKVDIVA HSMGGANTLY YIKNLDGGNK VANVVTLGGA NRLTTGKALP GTDPNQKILY

                                      170        180        190        200        210
                               ....|....| ....|....| ....|....| ....|....| ....|....| ....|.
WP_060597018.1_MMP03893210     TSVYSSADLI VVNSLSRLIG ARNVLIHGVG HIGLLASSQV KGYIKEGLNG GGQNTN
WP_060597018.1                 TSVYSSADLI VVNSLSRLIG ARNVLIHGVG HIGLLASSQV KGYIKEGLNG GGQNTN
tr|A0A7H4U7R2|A0A7H4U7R2_BACPU TSVYSSADLI VVNSLSRLIG ARNVLIHGVG HIGLLASSQV KGYIKEGLNG GGQNTN
tr|A0A2T0CZ91|A0A2T0CZ91_9BACI TSVYSSADLI VVNSLSRLIG ARNVLIHGVG HIGLLASSQV KGYIKEGLNG GGQNTN
WP_066030544.1 (H21)           TSVYSSADLI VVNSLSRLIG ARNVLIHGVG HIGLLASSQV KGYIKEGLNG GGQNTN
WP_034665530.1 (H16)           TSVYSSADLI VVNSLSRLIG ARNVLIHGVG HIGLLASSQV KGYIKEGLNG GGQNTN
tr|A1E152|A1E152_BACPU         TSVYSSADLI VVNSLSRLIG ARNVLIHGVG HIGLLASSQV KGYIKEGLNG VGQNTN
WP_095286143.1 (H22)           TSVYSSADLI VVNSLSRLIG ARNVLIHGVG HIGLLASSQV KGYIKEGLNG GGQNTN
```

(page 4 of 6) Continued from Supplementary Figure 9

```
tr|A0A267X6U6|A0A267X6U6_9BACI  TSVYSSADLI VVNSLSRLIG ARNVLIHGVG HIGLLASSQV KGYIKEGLNG GGQNTN
WP_106065766.1                  TSVYSSADMI VVNSLSRLIG ARNVLIHGVG HIGLLTSSQV KGYVKEGLNG GGQNTN
tr|A0A2T0CG63|A0A2T0CG63_BACPU  TSVYSSADMI VVNSLSRLIG ARNVLIHGVG HIGLLTSSQV KGYVKEGLNG GGQNTN
RAP25570.1                      TSVYSSADMI VVNSLSRLIG ARNVLIHGVG HIGLLTSSQV KGYVKEGLNG GGQNTN
KIL10233.1 (H4)                 TSVYSSADMI VVNSLSRLIG ARNVLIHGVG HIGLLTSSQV KGYVKEGLNG GGQNTN
WP_044142215.1                  TSVYSSADMI VVNSLSRLIG ARNVLIHGVG HIGLLTSSQV KGYVKEGLNG GGQNTN
WP_065098612.1 (H20)            TSVYSSADMI VVNSLSRLIG ARNVLIHGVG HISLLASSQV KGYIKEGLNG GGQNTN
tr|W8FKE7|W8FKE7_BACPU          TSVYSSADMI VVNSLSRLIG ARNVLIHGVG HISLLASSQV KGYIKEGLNG GGQNTN
WP_106066877.1 (H23)            TSVYSSADMI VVNSLSRLIG ARNVLIHGVG HISLLASSQV KGYIKEGLNG GGQNTN
RAU01106.1 (H9)                 TSVYSSADMI VVNSLSRLIG ARNVLIHGVG HISLLASSQV KGYIKEGLNG GGQNTN
WP_148666205.1                  TSVYSSADMI VVNSLSRLIG ARNVLIHGVG HIGLLASSQV KGYIKEGLNG GGQNTN
PRR93548.1 (H8)                 TSVYSSADMI VVNSLSRLIG ARNVLIHGVG HIGLLASSQV KGYIKEGLNG GGQNTN
WP_144521006.1 (H25)            TSVYSSADLI VVNSLSRLIG ARNVLIHGVG HIGLLTSSQV KGYIKEGLNG GGQNTN
WP_144486985.1 (H24)            TSVYSSADLI VVNSLSRLIG ARNVLIHGVG HIGLLTSSQV KGYIKEGLNG GGQNTN
WP_050943555.1_MMP03329560 (H1  TSVYSSADMI VVNSLSRLIG AKNVLIHGVG HIGLLTSSQV KGYIKEGLNG GGQNTN
WP_050943555.1_MMP08568348 (H1  TSVYSSADMI VVNSLSRLIG AKNVLIHGVG HIGLLTSSQV KGYIKEGLNG GGQNTN
WP_050943555.1 (H17)            TSVYSSADMI VVNSLSRLIG AKNVLIHGVG HIGLLTSSQV KGYIKEGLNG GGQNTN
tr|A0A4V5TAE0|A0A4V5TAE0_BACPU  TSVYSSADMI VVNSLSRLIG AKNVLIHGVG HIGLLTSSQV KGYIKEGLNG GGQNTN
tr|A0A1E1FNX8|A0A1E1FNX8_BACPU  TSVYSSADLI VVNSLSRLIG AKNVLIHGVG HIGLLTSSQV KGYIKEGLNG GGQNTN
tr|A0A2Y9UMQ0|A0A2Y9UMQ0_9BACI  TSVYSSADLI VVNSLSRLIG AKNVLIHGVG HIGLLTSSQV KGYIKEGLNG GGQNTN
WP_060697639.1_MMP03892176      TSVYSSADLI VVNSLSRLIG AKNVQIHGVG HIGLLTSSQV KGYIKEGLNG GGQNTN
WP_039180664.1_MMP03976074      TSVYSSADLI VVNSLSRLIG AKNVLIHGVG HIGLLTSSQV KGYIKEGLNG GGQNTN
PCK11675.1_MMP07270366 (H6)     TSVYSSADLI VVNSLSRLIG AKNVLIHGVG HIGLLTSSQV KGYIKEGLNG GGQNTN
tr|Q6RSN0|Q6RSN0_BACPU          TSVYSSADLI VVNSLSRLIG AKNVLIHGVG HIGLLTSSQV KGYIKEGLNG GGQNTN
WP_034624255.1_MMP02471694      TSVYSSADLI VVNSLSRLIG ARNVLIHGVG HIGLLTSSQV KGYIKEGLNG GGQNTN
WP_034624255.1_MMP06016472 (Li  TSVYSSADLI VVNSLSRLIG ARNVLIHGVG HIGLLTSSQV KGYIKEGLNG GGQNTN
OUZ07106.1_MMP05730644 (H5)     TSVYSSADLI VVNSLSRLIG ARNVLIHGVG HIGLLTSSQV KGYIKEGLNG GGQNTN
WP_169056738.1                  TSVYSSADLI VVNSLSRLIG ARNVLIHGVG HIGLLTSSQV KGYVKEGLNG GGQNTN
WP_058015050.1                  TSVYSSADMI VVNSLSRLIG ARNVLIHGVG HIGLLTSSQV KGYVKEGLNG GGQNTN
WP_106042678.1                  TSVYSSADMI VVNSLSRLIG ARNVLIHGVG HIGLLTSSQV KGYVKEGLNG GGQNTN
tr|H6U4T6|H6U4T6_9BACI          TSVYSSADLI VVNSLSRLIG ARNVLIHGVG HIGLLTSSQV KGYVKEGLNG GGQNTN
tr|W8RKH7|W8RKH7_BACPU          TSVYSSADLI VVNSLSRLIG ARNVLIHGVG HIGLLTSSQV KGYVKEGLNG GGQNTN
WP_025207804.1_MMP05216491      TSVYSSADLI VVNSLSRLIG ARNVLIHGVG HIGLLTSSQV KGYVKEGLNG GGQNTN
TFW47892.1_MMP10790525 (H11)    TSVYSSADLI VVNSLSRLIG ARNVLIHGVG HIGLLTSSQV KGYVKEGLNG GGQNTN
tr|A0A0N9DV87|A0A0N9DV87_9BACI  TSVYSSADLI VVNSLSRLIG ARNVLIHGVG HIGLLTSSQV KGYVKEGLNG GGQNTN
tr|A0A5K1KU97|A0A5K1KU97_9BACI  TSVYSSADLI VVNSLSRLIG ARNVLIHGVG HIGLLTSSQV KGYVKEGLNG GGQNTN
SPR92696.1_MMP4665500 (H10)     TSVYSSADLI VVNSLSRLIG ARNVLIHGVG HIGLLTSSQV KGYVKEGLNG GGQNTN
AJW76889.1 (H2)                 TSVYSSADLI VVNSLSRLIG ARNVLIHGVG HIGLLTSSQV KGYVKEGLNG GGQNTN
tr|A0A0D5CCJ2|A0A0D5CCJ2_9BACI  TSVYSSADLI VVNSLSRLIG ARNVLIHGVG HIGLLTSSQV KGYVKEGLNG GGQNTN
```

(page 5 of 6) Continued from Supplementary Figure 9

```
WP_007501788.1_MMP02471373          TSVYSSADLI VVNSLSRLIG ARNVLIHGVG HIGLLTSSQV KGYVKEGLNG GGQNTN
tr|M5RBD1|M5RBD1_9BACI              TSVYSSADLI VVNSLSRLIG ARNVLIHGVG HIGLLTSSQV KGYVKEGLNG GGQNTN
WP_041092577.1_MMP04263913          TSVYSSADLI VVNSLSRLIG ARNVLIHGVG HIGLLTSSQV KGYVKEGLNG GGQNTN
tr|A0A6G5TBN3|A0A6G5TBN3_9BACI      TSVYSSADLI VVNSLSRLIG ARNVLIHGVG HIGLLTSSQV KGYVKEGLNG GGQNTN
tr|Q8VU78|Q8VU78_9BACI              TSVYSSADLI VVNSLSRLIG ARNVLIHGVG HIGLLTSSQV KGYVKEGLNG GGQNTN
OPW99383.1_MMP05726035              TSVYSSADLI VVNSLSRLIG ARNVLIHGVG HIGLLTSSQV KGYVKEGLNG GGQNTN
tr|K4JG13|K4JG13_BACPU              TSVYSSADLI VVNSLSRLIG ARNVLIHGVG HIGLLTSSQV KGYVKEGLNG GGQNTN
WP_056705505.1_MMP11621488          TSVYSSADLI VVNSLSRLIG ARNVLIHGVG HIGLLTSSQV KGYVKEGLNG GGQNTN
WP_008356351.1_MMP02471164 (H1      TSIYSSADLI VVNSLSHLIG ARNVRIHGVG HIGLLTSSQV KGYVKEGLNG GGQNTN
tr|U3MG45|U3MG45_BACPU              TSVYSSADLI VVNSLSRLIG ARNVLIHGVG HIGLLTSSQV KGYVKEGLNG GGQNTN
WP_034322408.1_MMP02870955          TSVYSSADLI VVNSLSRLIG ARNVLIHGVG HIGLLASSQV NGYIKEGLNG GGQNTN
PCK20493.1_MMP07270068              TSVYSSADLI VVNSLSRLIG ARNVLIHGVG HIGLLASSQV NGYVKEGLNG VGQNTN
WP_007409033.1_MMP04296795 (H1      TSIYSSADLI VLNPLSRLIG GKNVQIHGVG HIGLLMNSQV NGLIKEGLNG GGQNTN
PHQ06896.1_MMP07514152 (H7)         TSIYSSADLI VLNPLSRLIG GKNVQIHGVG HIGLLMNSQV NGLIKEGLNG GGQNTN
AJC25179.1_MMP03272513 (H1)         TSIYSSADLI VLNPLSRLIG GKNVQIHGVG HIGLLMNSQV NGLIKEGLNG GGQNTN
WP_007409033.1_MMP09428134 (H1      TSIYSSADLI VLNPLSRLIG GKNVQIHGVG HIGLLMNSQV NGLIKEGLNG GGQNTN
WP_017419343.1_MMP05517979          TSIYSSADLI VMNPLSRLIG GKNVQIHGVG HIGLLMNSQV NGLIKEGLNG GGQNTN
WP_003327989.1_MMP02470933 (H1      TSVYSSADMI VMNPLSRLIG AKNVQIHGVG HIGLLMNSKV NGLIKEGLNG GGENTN
sp|P37957|ESTA_BACSU                TSIYSSADMI VMNYLSRLDG ARNVQIHGVG HIGLLYSSQV NSLIKEGLNG GGQNTN
QBJ80898.1_MMP09273200              TSIYSSADMI VMNYLSRLDG ARNVQIHGVG HIGLLYSSQV NSLIKEGLNG GGQNTN
AXC51599.1_MMP09500751 (H3)         TSIYSSADMI VMNYLSRLDG ARNVQIHGVG HIGLLYSSQV NSLIKEGLNG GGQNTN
WP_086343408.1_MMP11519450          TSIYSSADMI VMNYLSRLDG ARNVQIHGVG HIGLLMSSQV NSLIKEGLNG GGQNTN
WP_052471036.1_MMP13053465          TSIYSSADMI VMNYLSRLDG ARNVQIHGVG HIGLLYSSQV NSLIKEGLNG GGQNTN
```

(page 6 of 6) Continued from Supplementary Figure 9

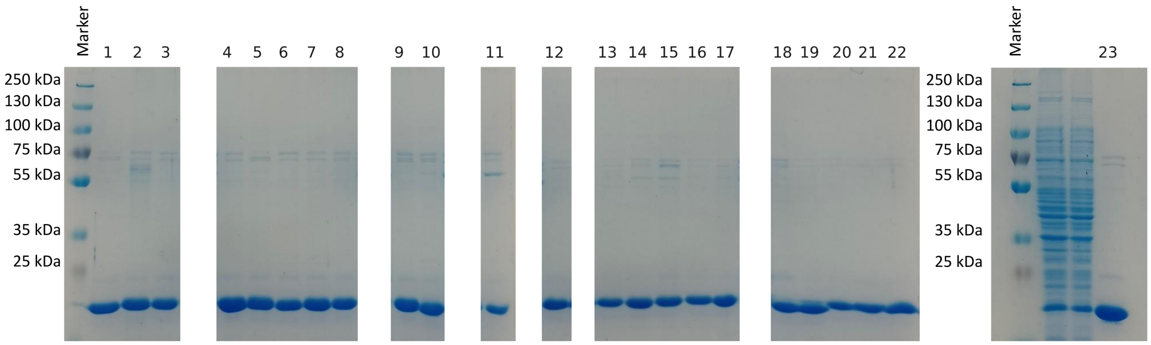

Supplementary Figure 10: SDS–PAGE analysis of *E. coli*–expressed Lip$_{MRD9}$ wildtype (WT) and its 29 variants (denoted with the subscript m) using a 12% Tris–glycine SDS–PAGE gel. Each gel includes a molecular weight marker (leftmost lane). As shown, expression of the N-terminal hexahistidine-tagged synthetic proteins confirmed that all variants were produced in soluble form. Variants (10 μg) analyzed include Lip$_{MRD9}$ WT; 1, AN39; 2, RA5; 3, AN23; 4, RA12; 5, AN38; 6, AN55; 7, AN9; 8, AN51; 9, AN14; 10, RA19; 11, RA4; 12, RA14; 13, RA13; 14, RA6; 15, AN67; 16, RA7; 17, RA18; 18, UPI000A479B17; 19, UPI00026BA38E; 20, WP_144521006.1; 21, UPI00025251CC; 22, WP_144486985.1; 23, WP_034665530.1 (soluble fraction); and 24, WP_034665530.1.

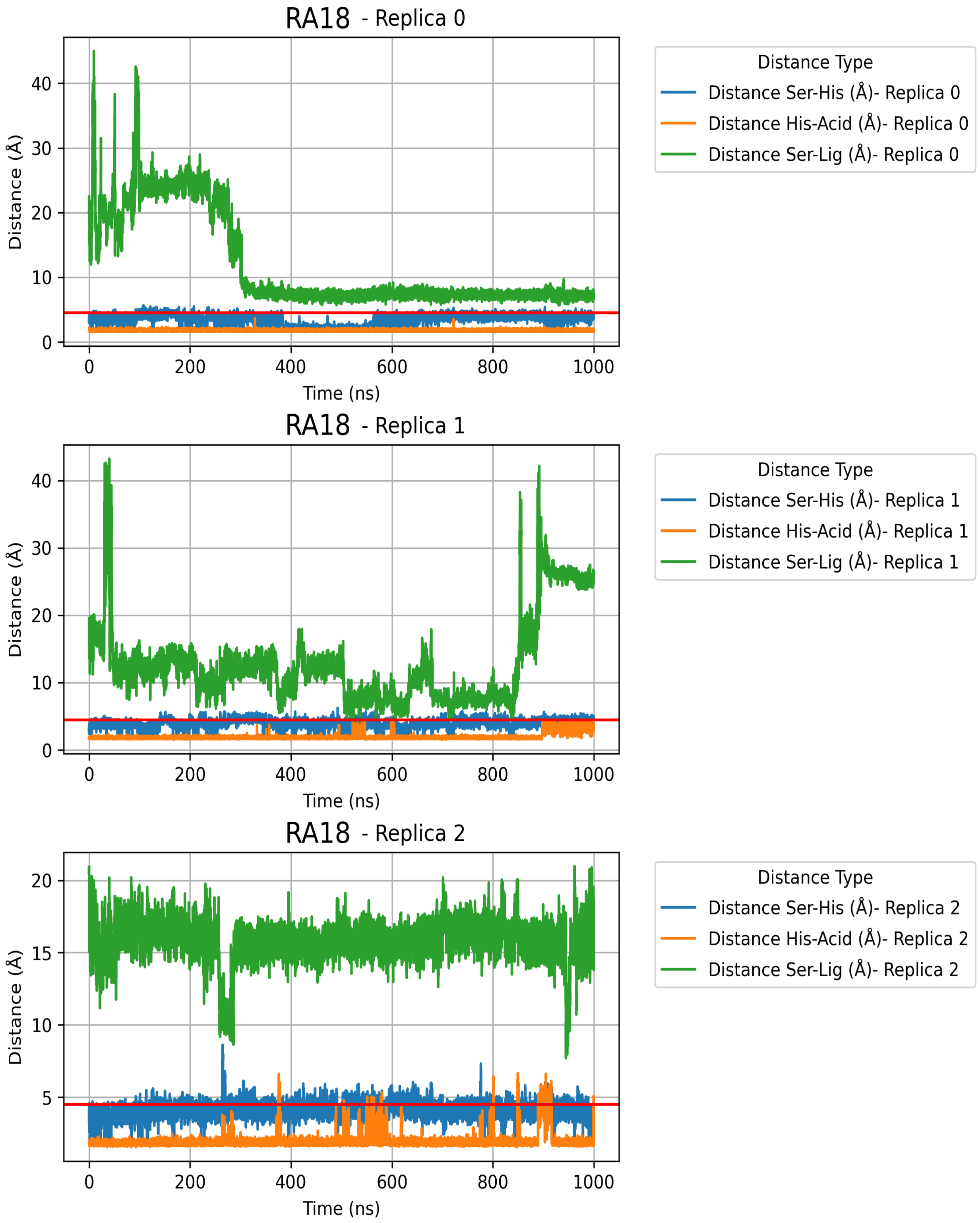

Supplementary Figure 11: Time evolution of catalytic-triad and ligand distances along three MD replicas for RA18 starting from a far-from-active-site pose. For each 1 µs replica, we monitored the Ser–His, His–acid (Asp/Glu), and Ser–ligand heavy-atom distances (in Å) as proxies for catalytic-triad integrity and substrate approach. The horizontal red line marks the 4.5 Å distance threshold used to define productive poses.

Supplementary Table 1: Structural and energetic characterization of selected Asitedesign variants from MD sampling and PELE simulations with TOL. For each enzyme design, the cumulative sampling percentage of productive binding poses (MD sum %) and the minimum Boltzmann PELE interaction energy in kcal/mol are reported, along with the mean ± standard deviation of three geometric parameters critical for catalysis: (i) RMSD relative to the reference active conformation (Å), (ii) distance between the catalytic serine Oy and the substrate carbonyl carbon (Ser–Lig Dist.), (iii) distance between the serine hydroxyl hydrogen (HG) and the histidine Nε2 nitrogen (Ser–His Dist.), and (iv) distance between the histidine proton and the aspartate carboxylate oxygen (His–Asp Dist.). The distributions of RMSD metric is shown as violin plots in Supplementary Fig. 2.

**Enzyme AsiteDesign_#**	**MD sum %**	**PELE (kcal/mol)**	**RMSD (Å)**	**Ser–Lig Dist. (Å)**	**Ser–His Dist. (Å)**	**His–Asp Dist. (Å)**
AN6	5.2320	-0.3044	1.21 ± 0.16	10.42 ± 5.00	5.09 ± 1.45	2.54 ± 1.37
AN36	2.9159	-0.3739	1.13 ± 0.16	11.33 ± 6.46	4.61 ± 2.29	2.72 ± 1.67
AN24	2.9995	-0.4661	1.14 ± 0.14	8.06 ± 3.32	3.88 ± 0.84	2.59 ± 1.47
AN85	0.2666	-1.0722	1.43 ± 0.29	9.11 ± 6.33	6.71 ± 2.40	5.30 ± 4.76
AN10	13.4049	-1.0776	1.25 ± 0.14	8.36 ± 3.66	3.66 ± 0.84	3.50 ± 1.88
AN39	15.8627	-1.5714	1.14 ± 0.14	7.87 ± 3.72	4.36 ± 1.42	4.19 ± 2.78
AN83	3.6741	-2.1783	1.24 ± 0.13	7.74 ± 3.51	5.13 ± 1.35	4.00 ± 1.61
AN51	19.4285	-2.4926	1.44 ± 0.27	5.63 ± 2.32	4.32 ± 1.48	2.62 ± 1.50
AN31	1.7329	-3.2149	1.06 ± 0.14	8.28 ± 2.20	4.93 ± 2.00	2.00 ± 0.52
AN9	13.5133	-3.8416	1.10 ± 0.12	7.85 ± 3.49	3.70 ± 0.63	1.97 ± 0.58
AN67	25.7352	-4.1140	1.07 ± 0.18	6.47 ± 2.81	3.18 ± 0.94	1.83 ± 0.15
AN66	0.3333	-4.7079	1.57 ± 0.33	9.79 ± 5.69	5.69 ± 2.72	6.63 ± 4.55
AN55	1.6912	-5.1759	1.22 ± 0.20	8.81 ± 5.57	5.57 ± 1.93	3.82 ± 1.76
AN23	4.1906	-7.8909	1.30 ± 0.24	7.57 ± 2.64	4.23 ± 1.01	5.78 ± 2.48
AN14	3.8241	-24.5579	1.24 ± 0.28	9.40 ± 4.26	4.28 ± 1.18	2.38 ± 1.32
AN38	0.4166	-31.8697	1.24 ± 0.24	10.78 ± 5.64	4.74 ± 1.27	4.47 ± 3.28
WildType LMRD9	**7.9897**	**-53.9063**	**1.21 ± 0.30**	**8.92 ± 3.22**	**3.36 ± 0.96**	**1.90 ± 0.30**

Supplementary Table 2: Structural and energetic characterization of selected AsiteDesign variants from MD sampling and PELE simulations with PT4. The column legends are described in Supplementary Table 1. The distributions of RMSD metric is shown as violin plots in Supplementary Fig. 1

**Enzyme AsiteDesign_#**	**MD sum %**	**PELE (kcal/mol)**	**RMSD (Å)**	**Ser–Lig Dist. (Å)**	**Ser–His Dist. (Å)**	**His–Asp Dist. (Å)**
AN85	0.0833	-11.4989	1.22 ± 0.12	7.96 ± 3.60	6.97 ± 1.72	3.28 ± 2.86
AN91	0.7748	-8.5222	1.11 ± 0.16	7.17 ± 1.65	3.80 ± 1.66	1.86 ± 0.21
AN53	1.7414	-6.6904	1.03 ± 0.13	9.78 ± 7.29	3.42 ± 0.98	1.84 ± 0.18
AN24	1.4121	-7.3564	1.42 ± 0.46	10.33 ± 4.60	4.94 ± 1.95	5.73 ± 2.50
AN19	2.0412	-0.5887	1.14 ± 0.13	6.46 ± 1.27	4.00 ± 1.38	3.18 ± 1.67
AN85	9.1061	-0.1613	1.24 ± 0.17	5.73 ± 2.02	4.67 ± 1.78	3.18 ± 2.06
AN83	10.5140	-6.1398	1.21 ± 0.15	6.34 ± 2.09	4.42 ± 1.38	3.77 ± 1.95
AN64	12.1553	-2.8286	1.20 ± 0.13	6.35 ± 2.35	4.69 ± 1.96	5.96 ± 2.90
AN9	17.6289	-5.6361	1.07 ± 0.13	5.32 ± 0.99	3.55 ± 0.64	1.87 ± 0.22
AN67	18.9119	-10.9002	1.10 ± 0.14	6.20 ± 1.67	3.34 ± 0.93	1.86 ± 0.14
AN55	21.7612	-9.4865	1.18 ± 0.26	4.89 ± 0.66	2.88 ± 1.18	1.98 ± 0.24
AN39	30.2841	-6.4206	1.02 ± 0.12	5.64 ± 2.04	3.44 ± 2.28	2.28 ± 0.22
AN36	36.1743	-44.3007	1.12 ± 0.17	5.95 ± 2.71	4.09 ± 0.80	2.96 ± 0.28
WildType Lip_{MRD9}	**37.1657**	**-12.5370**	**1.25 ± 0.40**	**4.42 ± 1.84**	**3.38 ± 1.06**	**4.92 ± 5.19**
AN51	42.1811	-27.6098	1.10 ± 0.20	6.22 ± 3.45	3.15 ± 0.94	1.84 ± 0.21
AN38	49.6792	-34.2084	1.16 ± 0.15	5.01 ± 1.97	2.96 ± 1.03	1.82 ± 0.17
AN23	60.0183	-8.7942	1.10 ± 0.11	4.15 ± 0.91	3.14 ± 1.07	2.08 ± 0.64
AN14	99.1669	-18.0724	0.95 ± 0.10	3.76 ± 0.25	3.53 ± 0.48	1.62 ± 0.10

Supplementary Table 3: Structural and energetic characterization of Rational Design variants from MD sampling and PELE simulations with PT4. The column legends are described in Supplementary Table 1. The distributions of RMSD metric is shown as violin plots in Supplementary Fig. 4

**Enzyme RA# (Mutations)**	**MD sum %**	**PELE (kcal/mol)**	**RMSD (Å)**	**Ser–Lig Dist. (Å)**	**Ser–His Dist. (Å)**	**His–Asp Dist. (Å)**
RA1 (H76S)	1.0664	-33.4387	1.38 ± 0.21	5.96 ± 2.39	8.91 ± 4.57	3.54 ± 3.54
RA16 (V136F)	1.4746	-49.8137	1.10 ± 0.21	8.00 ± 2.09	4.07 ± 0.81	2.26 ± 1.48
RA17 (L160F)	1.8079	-35.8804	1.21 ± 0.15	6.75 ± 1.92	4.98 ± 2.16	3.88 ± 3.37
RA2 (V137S)	2.1911	-14.5142	1.40 ± 0.31	5.01 ± 1.20	7.49 ± 2.84	5.31 ± 3.51
RA8 (H76N)	2.2661	-20.3371	1.20 ± 0.17	5.27 ± 1.35	6.42 ± 2.01	3.11 ± 1.80
RA9 (I12Y;I135N)	3.7657	-17.8906	1.38 ± 0.26	4.69 ± 0.96	7.46 ± 3.43	5.59 ± 3.21
RA15 (I12Y)	3.7824	-4.9947	1.38 ± 0.26	4.69 ± 0.96	7.46 ± 3.43	5.59 ± 3.21
RA3 (I135Y)	4.3656	-26.2556	1.23 ± 0.18	5.16 ± 1.64	6.82 ± 3.00	6.08 ± 4.31
RA4 (A105S)	12.6552	-5.7921	1.20 ± 0.14	4.91 ± 1.29	6.51 ± 2.49	3.82 ± 2.03
RA7 (L160A)	20.7032	-66.9005	1.35 ± 0.15	4.70 ± 1.34	5.14 ± 1.76	3.83 ± 2.35
RA14 (I135N)	36.0493	-18.9046	1.14 ± 0.12	5.06 ± 1.27	3.81 ± 1.98	2.10 ± 0.56
WildType Lip$_{MRD9}$	**37.1657**	**-12.5370**	**1.25 ± 0.40**	**4.42 ± 1.84**	**3.38 ± 1.06**	**4.92 ± 5.19**
RA11 (I12N)	47.6964	-1.8148	1.08 ± 0.12	4.33 ± 1.07	3.93 ± 1.47	4.34 ± 4.90
RA5 (I12N;I135Y)	47.6964	-81.5115	1.08 ± 0.12	4.33 ± 1.07	3.93 ± 1.07	4.34 ± 4.09
RA12 (G11S)	48.6378	-37.6698	1.06 ± 0.19	4.66 ± 1.11	3.52 ± 1.53	2.12 ± 0.77
RA13 (L108Y)	97.2590	-19.2666	1.17 ± 0.22	3.09 ± 0.22	2.33 ± 0.72	1.81 ± 0.12
RA18 (R57Y;L108Y)	68.3049	-2.0473	1.16 ± 0.11	3.29 ± 0.66	3.18 ± 1.12	3.37 ± 2.59
RA19 (R47N;K86C; R110K;L112C)	96.1343	-1.4778	0.96 ± 0.12	3.46 ± 0.49	3.17 ± 0.92	1.81 ± 0.10

Supplementary Table 4: Structural and energetic characterization of Rational Design variants from long MD sampling PT4. The column legends are described in Supplementary Table 1

Enzyme	***MD sum %***	***RMSD (Å)***	***Ser-Lig Dist. (Å)***	***Ser-His Dist. (Å)***	***His-Asp Dist. (Å)***
Wildtype	37.165	1.25 ± 0.40	4.42 ± 1.84	3.38 ± 1.06	4.92 ± 5.19
RA13 (L108Y)	97.2590	1.17 ± 0.22	3.09 ± 0.22	2.33 ± 0.72	1.81 ± 0.12
RA18 (R57Y;L108Y)	68.304	1.16 ± 0.11	3.29 ± 0.66	3.18 ± 1.11	3.37 ± 2.59

Supplementary Table 5: Structural and energetic characterization of homologs variants from MD sampling and PELE simulations with TOL.. For each enzyme design, the cumulative sampling percentage of productive binding poses (MD sum %) and the minimum Boltzmann PELE interaction energy in kcal/mol are reported, along with the mean ± standard deviation of three geometric parameters critical for catalysis: (i) RMSD relative to the reference active conformation (Å), (ii) distance between the catalytic serine Oy and the substrate carbonyl carbon (Ser–Lig Dist.), (iii) distance between the serine hydroxyl hydrogen (HG) and the histidine Nε2 nitrogen (Ser–His Dist.), and (iv) distance between the histidine proton and the aspartate carboxylate oxygen (His–Asp Dist.). The distributions of RMSD metric is shown as violin plots in Supplementary Fig. 7

Enzyme	***MD sum %***	***PELE Energy (kcal/mol)***	***RMSD (Å)***	***Ser-Lig Dist. (Å)***	***Ser-His Dist. (Å)***	***His-Asp Dist. (Å)***
HO21	0.049987	-17.445	1.34 ± 0.18	14.12 ± 7.60	4.65 ± 1.74	2.65 ± 1.37
HO14	0.04998	-1.7481	1.20 ± 0.18	17.40 ± 3.28	3.61 ± 0.57	2.21 ± 0.98
HO18	0.47488	-4.9034	1.17 ± 0.12	9.62 ± 2.22	3.72 ± 1.23	2.04 ± 0.49
HO15	0.48321	-46.410	1.10 ± 0.16	7.12 ± 2.56	5.15 ± 2.60	2.17 ± 0.75
HO17	0.6331	-63.721	1.04 ± 0.12	10.27 ± 2.62	3.31 ± 0.83	1.85 ± 0.18
HO19	0.8497	-5.3009	1.04 ± 0.12	10.27 ± 2.62	3.31 ± 0.83	1.85 ± 0.18
HO13	1.0997	-44.956	1.11 ± 0.21	12.13 ± 4.85	4.14 ± 0.88	4.56 ± 1.56
HO5	1.5579	-53.111	1.17 ± 0.17	8.47 ± 2.28	3.98 ± 1.11	3.93 ± 1.95
HO12	1.6495	-42.540	1.16 ± 0.17	8.33 ± 2.91	4.62 ± 1.75	3.93 ± 3.17
HO4	1.8911	-1.0385	1.16 ± 0.16	8.48 ± 3.66	4.13 ± 0.60	3.02 ± 1.54
HO8	1.9495	-63.370	1.16 ± 0.18	8.38 ± 2.46	3.84 ± 0.87	4.08 ± 2.02
HO11	2.2994	-58.580	1.11 ± 0.13	7.60 ± 2.37	4.44 ± 1.92	8.69 ± 2.36
HO25	2.8159	-0.0119	1.02 ± 0.14	8.13 ± 2.35	3.32 ± 0.85	1.84 ± 0.15
HO22	3.8323	-0.8997	1.13 ± 0.15	8.80 ± 3.12	3.69 ± 0.84	2.64 ± 1.47
HO7	3.9406	-4.5268	1.26 ± 0.16	8.71 ± 3.18	4.02 ± 1.63	4.78 ± 2.43
HO2	3.9740	-9.9271	1.19 ± 0.16	7.80 ± 3.41	4.35 ± 1.09	3.95 ± 2.05
HO23	4.3739	-60.484	1.23 ± 0.21	9.55 ± 3.71	5.56 ± 2.56	3.62 ± 2.38
HO1	5.5486	-13.697	1.41 ± 0.25	8.78 ± 2.67	3.76 ± 1.00	2.06 ± 0.71
HO20	5.9068	-79.090	1.22 ± 0.14	10.20 ± 4.10	4.20 ± 0.58	3.12 ± 1.85
HO10	6.1484	-67.832	0.98 ± 0.13	7.52 ± 2.31	3.32 ± 0.92	1.91 ± 0.29
WildType Lip_{MRD9}	7.9896	-53.906	1.21 ± 0.30	8.92 ± 3.22	3.36 ± 0.96	1.90 ± 0.30
HO9	9.7808	-42.791	1.26 ± 0.17	7.94 ± 3.61	3.47 ± 1.38	3.34 ± 2.74
HO16	18.128	-40.117	1.10 ± 0.12	7.19 ± 2.70	3.24 ± 1.02	1.83 ± 0.13
HO3	21.719	-0.5722	1.28 ± 0.25	8.89 ± 3.84	6.76 ± 3.57	3.47 ± 2.25
HO24	22.502	-44.677	1.09 ± 0.15	7.40 ± 3.46	3.84 ± 0.51	1.82 ± 0.10
HO6	62.392	-54.825	1.21 ± 0.12	4.40 ± 1.32	4.09 ± 0.70	1.83 ± 0.12

Supplementary Table 6: Structural and energetic characterization of homologs variants from MD sampling and PELE simulations with PT4.. For each enzyme design, the cumulative sampling percentage of productive binding poses (MD sum %) and the minimum Boltzmann PELE interaction energy in kcal/mol are reported, along with the mean ± standard deviation of three geometric parameters critical for catalysis: (i) RMSD relative to the reference active conformation (Å), (ii) distance between the catalytic serine Oγ and the substrate carbonyl carbon (Ser–Lig Dist.), (iii) distance between the serine hydroxyl hydrogen (HG) and the histidine Nε2 nitrogen (Ser–His Dist.), and (iv) distance between the histidine proton and the aspartate carboxylate oxygen (His–Asp Dist.). The distributions of RMSD metric is shown as violin plots in Supplementary Fig. 8

Enzyme	***MD sum %***	***PELE Energy (kcal/mol)***	***RMSD (Å)***	***Ser-Lig Dist. (Å)***	***Ser-His Dist. (Å)***	***His-Asp Dist. (Å)***
HO4	1.4996	-36.340	1.29 ± 0.17	4.68 ± 1.02	4.08 ± 0.84	6.08 ± 2.14
HO8	2.8742	-5.9386	1.05 ± 0.13	6.17 ± 1.16	4.20 ± 1.63	2.22 ± 0.98
HO21	2.8909	-8.1020	1.25 ± 0.15	5.73 ± 1.01	4.51 ± 1.30	2.94 ± 1.98
HO22	5.4153	-3.2340	1.16 ± 0.13	5.27 ± 1.12	4.83 ± 0.98	2.20 ± 0.90
HO19	8.4395	-3.3850	1.18 ± 0.13	7.04 ± 2.25	3.30 ± 0.88	2.27 ± 1.18
HO23	9.3976	-19.619	1.68 ± 0.28	6.24 ± 1.66	3.45 ± 1.46	2.52 ± 1.36
HO14	9.5392	-2.8689	1.19 ± 0.12	4.30 ± 1.24	4.01 ± 0.61	6.31 ± 2.61
HO17	10.614	-4.0108	1.26 ± 0.32	5.00 ± 1.41	4.88 ± 1.69	1.95 ± 0.43
HO13	14.013	-30.380	1.26 ± 0.32	4.97 ± 1.98	4.73 ± 1.18	3.31 ± 1.69
HO15	17.170	-17.346	1.42 ± 0.37	6.30 ± 2.59	4.46 ± 2.08	3.47 ± 2.11
HO3	19.370	-2.2816	1.09 ± 0.18	6.48 ± 3.03	3.51 ± 0.89	1.85 ± 0.18
HO10	19.495	-15.160	1.22 ± 0.20	4.57 ± 0.95	5.14 ± 1.69	2.91 ± 1.96
HO12	20.686	-25.613	1.31 ± 0.18	5.60 ± 1.83	3.91 ± 0.58	4.00 ± 1.55
HO11	21.762	-9.6605	1.27 ± 0.13	7.72 ± 3.15	3.53 ± 0.98	3.81 ± 2.06
HO2	22.236	-11.018	1.29 ± 0.22	7.57 ± 2.92	3.56 ± 0.91	3.20 ± 1.72
HO20	23.435	-26.751	1.06 ± 0.16	6.28 ± 2.34	3.32 ± 0.88	2.61 ± 1.47
HO18	31.508	-34.099	1.26 ± 0.32	5.00 ± 1.41	4.88 ± 1.69	1.95 ± 0.43
HO16	36.399	-11.681	1.29 ± 0.13	5.03 ± 1.53	4.73 ± 2.15	2.70 ± 1.72
WildType Lip_{MRD9}	37.165	-12.537	1.25 ± 0.40	4.42 ± 1.84	3.38 ± 1.06	4.92 ± 5.19
HO1	41.856	-40.469	1.33 ± 0.17	4.37 ± 1.11	3.70 ± 1.27	2.11 ± 0.76
HO7	42.339	-4.0025	1.41 ± 0.38	4.98 ± 1.86	4.02 ± 0.83	2.00 ± 0.22
HO9	47.796	-25.672	1.11 ± 0.12	4.61 ± 1.67	4.24 ± 0.68	1.97 ± 0.35
HO5	57.948	-6.809	1.15 ± 0.12	4.39 ± 0.88	3.87 ± 0.58	1.95 ± 0.58
HO25	74.173	-22.133	1.12 ± 0.12	3.91 ± 1.39	2.86 ± 0.91	1.81 ± 0.11
HO25	75.289	-20.645	1.06 ± 0.10	3.77 ± 0.88	3.12 ± 0.99	2.09 ± 0.88
HO6	90.619	-12.861	1.25 ± 0.20	3.10 ± 0.22	2.53 ± 0.88	2.14 ± 0.75

Supplementary Table 7: Experimental values for production and relative enzymatic activities against Lip_{MRD9} wildtype for all expressed variants. Production yield (0.226 – 1.58 mg/L) and relative activities (R.A.) towards *p*-nitrophenyl caprylate (pNPC10) and $nPET_{GFa}$ substrates (normalized to the wildtype Lip_{MRD9}, set to 100%) are presented for selected engineered variants.

Variant	Production (mg/L)	R.A. pNPC10 (%)1	R.A. $nPET_{GFa}$ (%)2	Mutations
Lip_{MRD9}	0,656	100	100	-
AN9	0,259	32.10 ± 2.61	4.74 ± 8.21	M78A;I135Q
AN14	0,395	33.91 ± 12.32	1.15 ± 2.00	L108A;I135Q
AN23	0,365	87.11 ± 3.96	118.46 ± 4.81	F20A;W31F
AN36	0,698	91.09 ± 36.92	12.83 ± 2.08	L108A
AN38	0,223	38.46 ± 5.48	5.36 ± 0.46	I12M;I135K
AN39	0,277	50.16 ± 15.28	30.13 ± 0.63	I12A;T45G;L108A
AN51	0,048	0	0	F20A;W31F;M78A
AN55	0,378	37.22 ± 8.17	10.67 ± 1.31	M78A;I135A
AN67	0,686	133.49 ± 13.81	37.82 ± 2.61	I102A
RA4	0,37	85.99 ± 15.42	82.00 ± 1.74	A105S
RA5	0,226	23.01 ± 4.66	11.45 ± 0.56	I12N;I135Y
RA6	0,506	13.97 ± 2.63	85.31 ± 7.08	N16E
RA12	0,394	2.91 ± 0.79	1.68 ± 2.91	G11S
RA13	0,35	134.73 ± 29.38	279.56 ± 5.27	L108Y
RA14	1,58	59.69 ± 25.54	9.26 ± 16.05	I135N
RA18	0,515	42.00 ± 12.99	285.00 ± 6.61	R57Y;L108Y
RA19	0,269	87.58 ± 14.24	142.65 ± 49.74	R47N,K86C,R110K,L112C
HO1	0,361	92.88 ± 6.97	101.01 ± 2.54	UPI00026BA38E
HO6	0,244	125.00 ± 17.42	163.26 ± 2.45	UPI00025251CC
HO9	0,344	213.12 ± 34.31	106.21 ± 1.36	UPI000A479B17
HO16	0,542	259.23 ± 16.05	139.73 ± 9.18	WP_034665530.1
HO24	0,42	124.68 ± 11.06	105.88 ± 4.54	WP_144486985.1
HO25	0,417	101.74 ± 8.10	132.13 ± 3.13	WP_144521006.1

[1]Hydrolysis of pNPC10 was assayed with 0.02 mg/mL enzyme and 1.2 mM substrate (from a 40 mM stock in acetonitrile) in 100 μL of 40 mM HEPES, 75 mM NaCl, pH 7.0, at 40 °C for 10 min. In this assay, 100% activity corresponds to that of wildtype Lip_{MRD9} under the same conditions: 0.015 units/mg.

[2]PET hydrolysis ($nPET_{GFa}$) was performed with 4 μg enzyme and 1.7 mg/mL substrate in a total volume of 50 μL containing 20 mM HEPES, 75 mM NaCl, pH 7.0, at 40 °C for 4 h with shaking at 950 rpm. In this assay, 100% activity corresponds to that of wildtype Lip_{MRD9} under the same conditions: 1092 ± 6 μM degradation products.

Supplementary Table 8: Conserved and divergent amino acid residues across homologous sequences. The table lists amino acid residues at conserved positions across the aligned sequences, highlighting substitutions that may underlie functional divergence. Rows correspond to structurally or functionally relevant residue positions (e.g., those near the catalytic triad, binding cleft, or flexible loops). Columns list the amino acid present at each position in a given homolog. Color coding indicates sequence divergence: Blue: Residues conserved across most variants. Pink/Red: Non-conservative substitutions potentially linked to altered substrate specificity or thermostability. Purple: Conservative substitutions. Orange: Rare variants or those of uncertain effect.

**Lip$_{MRD9}$**		HO1		HO6		HO9		HO36		HO24		HO25	
**Pos.**	**A.**	Pos.	A.	Pos.	A.	Pos.	A.	Pos.	A.	Pos.	A.	Pos.	A.
**12**	**I**	12	I	12	I	12	M	12	M	12	I	12	I
**17**	**Y**	17	F	17	Y	17	Y	17	Y	17	Y	17	Y
**20**	**F**	20	A	20	Y	20	A	20	A	20	A	20	A
**21**	**S**	21	G	21	S	21	S	21	S	21	S	21	S
**24**	**S**	24	T	24	S	24	S	24	S	24	S	24	S
**27**	**A**	27	A	27	V	27	V	27	V	27	V	27	V
**28**	**T**	28	S	28	G	28	T	28	S	28	T	28	T
**32**	**D**	32	S	32	D	32	D	32	D	32	D	32	D
**35**	**Q**	35	E	35	Q	35	Q	35	Q	35	Q	35	Q
**36**	**L**	36	M	36	L	36	L	36	L	36	L	36	L
**37**	**Y**	37	Y	37	Y	37	F	37	F	37	F	37	F
**42**	**I**	42	L	42	I	42	I	42	I	42	I	42	I
**50**	**N**	50	H	50	N	50	N	50	N	50	N	50	N
**52**	**G**	52	A	52	G	52	G	52	G	52	G	52	G
**57**	**R**	57	N	57	R	57	R	57	R	57	R	57	R
**58**	**F**	58	Y	58	F	58	F	58	F	58	F	58	F
**60**	**K**	60	K	60	Q	60	K	60	K	60	K	60	K
**61**	**D**	61	K	61	D	61	D	61	D	61	D	61	D
**64**	**D**	64	S	64	D	64	G	64	A	64	D	64	D
**65**	**K**	65	E	65	K	65	K	65	K	65	K	65	K
**87**	**I**	87	I	87	I	87	I	87	I	87	I	87	V
**97**	**E**	97	A	97	E	97	E	97	E	97	E	97	E
**102**	**I**	102	L	102	I	102	L	102	L	102	I	102	I
**110**	**S**	110	T	110	S	110	S	110	S	110	S	110	S
**111**	**S**	111	N	111	N	111	L	111	L	111	S	111	S
**128**	**V**	128	I	128	V	128	V	128	V	128	V	128	V
**134**	**L**	134	L	134	L	134	M	134	L	134	L	134	L
**137**	**V**	137	L	137	V	137	V	137	V	137	V	137	V
**139**	**S**	139	P	139	S	139	S	139	S	139	S	139	S
**146**	**A**	146	G	146	A	146	A	146	A	146	A	146	A
**147**	**R**	147	K	147	K	147	R	147	R	147	R	147	R
**150**	**L**	150	Q	150	L	150	L	150	L	150	L	150	L
**158**	**G**	158	G	158	G	158	S	158	G	158	G	158	G
**161**	**T**	161	M	161	T	161	A	161	A	161	T	161	T
**162**	**S**	162	N	162	S	162	S	162	S	162	S	162	S
**166**	**K**	166	N	166	K	166	K	166	K	166	K	166	K
**168**	**Y**	168	L	168	Y	168	Y	168	Y	168	Y	168	Y

Supplementary Table 9: Correspondence between sequence identifiers and the abbreviated labels (HO1–HO25) used throughout the manuscript. Each entry lists the full sequence accession number and its corresponding shorthand used in figures and main text.

**Sequence ID**	**Reference**
AJC25179.1_MMP03272513	HO1
AJW76889.1	HO2
AXC51599.1_MMP09500751	HO3
KIL0233.1	HO4
OUZ07106.1_MMP05730644	HO5
PCK11675.1_MMP07270366	HO6
PHQ08696.1_MMP07514152	HO7
PRR93548.1	HO8
RAU01106.1	HO9
SPR92696.1_MMP4665500	HO10
TFW47892.1_MMP10790525	HO11
WP_003327989.1_MMP02470933	HO12
WP_007409033.1_MMP04296795	HO13
WP_007409033.1_MMP09428134	HO14
WP_008356351.1_MMP02471164	HO15
WP_034665530.1	HO16
WP_050943555.1	HO17
WP_050943555.1_MMP03329560	HO18
WP_050943555.1_MMP08586348	HO19
WP_065098612.1	HO20
WP_066030544.1	HO21
WP_095286143.1	HO22
WP_100606877.1	HO23
WP_144486985.1	HO24
WP_144521006.1	HO25

Supplementary Table 10: Correspondence between sequence identifiers and the abbreviated labels (RA1–RA19) used throughout the manuscript. Each entry lists the rational mutations and its corresponding shorthand used in figures and main text.

**Reference**	**Mutations**
RA1	H76S
RA2	V137S
RA3	I135Y
RA4	A105S
RA5	I12N;I135Y
RA6	N16E
RA7	L160A
RA8	H76N
RA9	I12Y;I135N
RA10	L158N
RA11	I12N
RA12	G11S
RA13	L108Y
RA14	I135N
RA15	I12Y
RA16	V136F
RA17	L160F
RA18	R57Y;L108Y
RA19	R47N;K86C;R110K;L112C

Supplementary Table 11: PROPKA-predicted pKa values for catalytic histidine and acidic triad residues

Enzyme	pka_his	pka_acid
AN14	6.3	3.08
AN23	6.48	3.07
AN36	6.37	3.28
AN38	6.43	3.46
AN39	6.53	3.25
AN51	6.41	3.27
AN55	6.93	3.04
AN67	6.23	3.2
AN9	6.3	3.43
HO1	6.48	3.72
HO16	6.12	3.8
HO24	6.28	3.08
HO25	5.94	2.02
HO6	6.23	3.7
HO9	6.11	2.32
RA12	6.28	2.76
RA13	6.7	3.39
RA14	6.35	2.71
RA18	6.7	3.39
RA19	6.35	2.69
RA4	6.3	2.75
RA5	6.27	2.81
RA6	6.38	2.7
Lip_{MRD9}	6.31	2.74

Supplementary Table 12: Per-replica summary of the 1 μs MD simulations with substrate placement (docked vs far-from-active-site)

Enzyme	Initial configuration	Reps	Time (μs)	Distance (Å)
WT	Docked (active site)	3	1.0	Close
RA13 (L108Y)	Docked (active site)	3	1.0	Close
RA18 (R57Y;L108Y)	Docked (active site)	3	1.0	Close
RA18 (R57Y;L108Y)	Far-from-active-site	3	1.0	∼25.0

Supplementary Table 13: Per-atom partial charges for the TOL substrate. Charges were obtained by Jaguar electrostatic-potential (ESP) fitting from a DFT(B3LYP-D3)/cc-pVTZ single-point calculation with implicit solvation (Jaguar SOLV) and are reported exactly as stored in the Maestro file (`r_j_ESP_Charges` exported as `r_m_charge1`). Atom indices and names follow the Maestro/PDB naming.

Substrate	Atom index	Atom name	Element	Partial charge
TOL	1	O1	O	-0.43748
TOL	2	O2	O	-0.32101
TOL	3	O3	O	-0.57093
TOL	4	O4	O	-0.61751
TOL	5	O5	O	-0.63169
TOL	6	O6	O	-0.64218
TOL	7	C1	C	-0.10053
TOL	8	C2	C	0.05181
TOL	9	C3	C	-0.01243
TOL	10	C4	C	-0.18919
TOL	11	C5	C	0.15551
TOL	12	C6	C	0.08817
TOL	13	C7	C	-0.04827
TOL	14	C8	C	0.09751
TOL	15	C9	C	0.25319
TOL	16	C10	C	-0.03948
TOL	17	C11	C	0.16021
TOL	18	C12	C	0.31063
TOL	19	C13	C	-0.10236
TOL	20	C14	C	9.00E-05
TOL	21	C15	C	0.00786

TOL	22	C16	C	0.04591
TOL	23	C17	C	-0.16007
TOL	24	C18	C	0.03136
TOL	25	C19	C	-0.25677
TOL	26	C20	C	-0.0525
TOL	27	C21	C	-0.06329
TOL	28	C22	C	-0.02967
TOL	29	C23	C	0.11231
TOL	30	C24	C	-0.01282
TOL	31	C25	C	-0.05211
TOL	32	C26	C	0.28697
TOL	33	C27	C	0.08873
TOL	34	C28	C	0.0694
TOL	35	C29	C	0.55217
TOL	36	C30	C	0.02028
TOL	37	C31	C	0.45531
TOL	38	C32	C	0.05545
TOL	39	C33	C	0.00133
TOL	40	C34	C	-0.09277
TOL	41	C35	C	-0.04619
TOL	42	C36	C	0.3871
TOL	43	C37	C	-0.183
TOL	44	C38	C	-0.45654
TOL	45	C39	C	-0.26205
TOL	46	C40	C	-0.23296
TOL	47	C41	C	-0.41985

TOL	48	C42	C	0.32699
TOL	49	C43	C	0.29654
TOL	50	C44	C	0.26957
TOL	51	C45	C	-0.46617
TOL	52	C46	C	-0.18701
TOL	53	C47	C	-0.20107
TOL	54	C48	C	-0.44032
TOL	55	C49	C	0.17976
TOL	56	C50	C	0.05699
TOL	57	C51	C	0.3513
TOL	58	C52	C	0.70678
TOL	59	C53	C	0.77793
TOL	60	C54	C	0.83063
TOL	61	C55	C	-0.26632
TOL	62	C56	C	-0.28423
TOL	63	C57	C	-0.31929
TOL	64	H1	H	0.01565
TOL	65	H2	H	0.02325
TOL	66	H3	H	0.01373
TOL	67	H4	H	-0.05317
TOL	68	H5	H	-0.00013
TOL	69	H6	H	0.01758
TOL	70	H7	H	0.06226
TOL	71	H8	H	0.04466
TOL	72	H9	H	-0.05424
TOL	73	H10	H	-0.01954

TOL	74	H11	H	-0.04544
TOL	75	H12	H	-0.01016
TOL	76	H13	H	0.03765
TOL	77	H14	H	0.05525
TOL	78	H15	H	-0.0469
TOL	79	H16	H	-0.04077
TOL	80	H17	H	-0.06842
TOL	81	H18	H	-0.07329
TOL	82	H19	H	0.00794
TOL	83	H20	H	0.01967
TOL	84	H21	H	-0.04006
TOL	85	H22	H	-0.02933
TOL	86	H23	H	-0.08905
TOL	87	H24	H	-0.08036
TOL	88	H25	H	0.0266
TOL	89	H26	H	0.00965
TOL	90	H27	H	-0.03947
TOL	91	H28	H	-0.01233
TOL	92	H29	H	-0.00367
TOL	93	H30	H	-0.00849
TOL	94	H31	H	0.01749
TOL	95	H32	H	0.00368
TOL	96	H33	H	0.01628
TOL	97	H34	H	0.00633
TOL	98	H35	H	-0.01085
TOL	99	H36	H	0.01741

TOL	100	H37	H	0.03797
TOL	101	H38	H	0.03524
TOL	102	H39	H	-0.0191
TOL	103	H40	H	-0.02191
TOL	104	H41	H	-0.01676
TOL	105	H42	H	-0.00202
TOL	106	H43	H	-0.0419
TOL	107	H44	H	-0.01775
TOL	108	H45	H	-0.0363
TOL	109	H46	H	-0.00785
TOL	110	H47	H	-0.01888
TOL	111	H48	H	-0.01876
TOL	112	H49	H	0.04784
TOL	113	H50	H	0.02284
TOL	114	H51	H	-0.00462
TOL	115	H52	H	-0.14095
TOL	116	H53	H	0.00336
TOL	117	H54	H	-0.01455
TOL	118	H55	H	0.02662
TOL	119	H56	H	0.01267
TOL	120	H57	H	-0.0699
TOL	121	H58	H	-0.08903
TOL	122	H59	H	0.0669
TOL	123	H60	H	0.01046
TOL	124	H61	H	0.00941
TOL	125	H62	H	-0.04385

TOL	126	H63	H	-0.03148
TOL	127	H64	H	-0.03874
TOL	128	H65	H	-0.01489
TOL	129	H66	H	-0.00715
TOL	130	H67	H	0.0057
TOL	131	H68	H	0.01754
TOL	132	H69	H	0.06505
TOL	133	H70	H	0.03875
TOL	134	H71	H	-0.03683
TOL	135	H72	H	-0.0971
TOL	136	H73	H	0.08511
TOL	137	H74	H	0.06898
TOL	138	H75	H	0.09805
TOL	139	H76	H	0.14576
TOL	140	H77	H	0.09694
TOL	141	H78	H	0.08391
TOL	142	H79	H	0.13598
TOL	143	H80	H	0.14574
TOL	144	H81	H	-0.07297
TOL	145	H82	H	-0.07935
TOL	146	H83	H	-0.06178
TOL	147	H84	H	-0.05513
TOL	148	H85	H	-0.01502
TOL	149	H86	H	-0.02857
TOL	150	H87	H	0.15043
TOL	151	H88	H	0.12352

TOL	152	H89	H	0.13029
TOL	153	H90	H	0.15812
TOL	154	H91	H	0.08758
TOL	155	H92	H	0.06899
TOL	156	H93	H	0.09187
TOL	157	H94	H	0.06509
TOL	158	H95	H	0.01136
TOL	159	H96	H	0.04397
TOL	160	H97	H	0.04187
TOL	161	H98	H	0.04428
TOL	162	H99	H	0.04835
TOL	163	0H10	H	0.04161
TOL	164	1H10	H	0.05932
TOL	165	2H10	H	0.06066
TOL	166	3H10	H	0.05521
TOL	167	4H10	H	0.06072

Supplementary Table 14: Per-atom partial charges for the PT4 substrate. Charges were obtained by Jaguar electrostatic-potential (ESP) fitting from a DFT(B3LYP-D3)/cc-pVTZ single-point calculation with implicit solvation (Jaguar SOLV) and are reported exactly as stored in the Maestro file (`r_j_ESP_Charges` exported as `r_m_charge1`). Atom indices and names follow the Maestro/PDB naming.

Substrate	Atom index	Atom name	Element	Partial charge
PT4	1	C29	C	0.10405
PT4	2	C28	C	0.09121
PT4	3	O9	O	-0.35468
PT4	4	C20	C	0.65884

PT4	5	O8	O	-0.57477
PT4	6	C21	C	-0.05163
PT4	7	C27	C	-0.0931
PT4	8	C26	C	-0.07814
PT4	9	C24	C	-0.13203
PT4	10	C25	C	0.72959
PT4	11	O10	O	-0.59773
PT4	12	O11	O	-0.38161
PT4	13	C39	C	0.15695
PT4	14	C38	C	0.03177
PT4	15	O14	O	-0.31941
PT4	16	C30	C	0.65552
PT4	17	O13	O	-0.57078
PT4	18	C31	C	-0.07574
PT4	19	C37	C	-0.08816
PT4	20	C36	C	-0.03698
PT4	21	C34	C	-0.12633
PT4	22	C35	C	0.74
PT4	23	O15	O	-0.62816
PT4	24	O16	O	-0.4032
PT4	25	C40	C	0.34312
PT4	26	C41	C	-0.31225
PT4	27	C33	C	-0.11087
PT4	28	C32	C	-0.07804
PT4	29	C23	C	-0.0396
PT4	30	C22	C	-0.12915

PT4	31	O12	O	-0.36365
PT4	32	C15	C	0.65766
PT4	33	O7	O	-0.57243
PT4	34	C14	C	-0.06003
PT4	35	C16	C	-0.12338
PT4	36	C17	C	-0.05433
PT4	37	C11	C	-0.14412
PT4	38	C12	C	-0.09152
PT4	39	C13	C	-0.07747
PT4	40	C10	C	0.72657
PT4	41	O5	O	-0.59354
PT4	42	O6	O	-0.34161
PT4	43	C18	C	0.05476
PT4	44	C19	C	0.10406
PT4	45	O3	O	-0.42059
PT4	46	C5	C	0.83209
PT4	47	O2	O	-0.62707
PT4	48	C4	C	-0.17561
PT4	49	C6	C	-0.0722
PT4	50	C7	C	-0.10266
PT4	51	C1	C	-0.08609
PT4	52	C2	C	-0.0833
PT4	53	C3	C	-0.09894
PT4	54	C	C	0.72411
PT4	55	O1	O	-0.41817
PT4	56	C8	C	0.37791

PT4	57	C9	C	-0.34509
PT4	58	O	O	-0.62774
PT4	59	H23	H	0.0961
PT4	60	H24	H	0.07831
PT4	61	H21	H	0.07831
PT4	62	H22	H	0.10738
PT4	63	H20	H	0.12957
PT4	64	H19	H	0.12372
PT4	65	H31	H	0.05555
PT4	66	H32	H	0.08595
PT4	67	H30	H	0.08211
PT4	68	H29	H	0.11154
PT4	69	H28	H	0.10202
PT4	70	H27	H	0.11785
PT4	71	H33	H	0.01504
PT4	72	H34	H	0.00794
PT4	73	HC41	H	0.08746
PT4	74	H35	H	0.08939
PT4	75	H36	H	0.09729
PT4	76	H26	H	0.13402
PT4	77	H25	H	0.12618
PT4	78	H18	H	0.10606
PT4	79	H17	H	0.11845
PT4	80	H11	H	0.13727
PT4	81	H12	H	0.11998
PT4	82	H9	H	0.13592

PT4	83	H10	H	0.11916
PT4	84	H13	H	0.08356
PT4	85	H14	H	0.09589
PT4	86	H16	H	0.11125
PT4	87	H15	H	0.07856
PT4	88	H2	H	0.13397
PT4	89	H3	H	0.12967
PT4	90	H	H	0.12942
PT4	91	H1	H	0.14109
PT4	92	H4	H	0.00545
PT4	93	H5	H	0.00513
PT4	94	HC9	H	0.1017
PT4	95	H7	H	0.0954
PT4	96	H6	H	0.10004

References

.
